# Supplementary material for: PICNIC accurately predicts condensate-forming proteins regardless of their structural disorder across organisms
Source: Nat Commun. 2024 Dec 11;15:10668. doi: 10.1038/s41467-024-55089-x (PMC11634905; doi:10.1038/s41467-024-55089-x)
Supplement: Supplementary file 1 — Supplementary Information [file 41467_2024_55089_MOESM1_ESM.pdf]

Supplementary Information

PICNIC accurately predicts condensate-forming proteins regardless of their structural disorder across organisms

Anna Hadarovich[1,2#], Hari Raj Singh[1#], Soumyadeep Ghosh[1,2], Maxim Scheremetjew[1,2], Nadia Rostam[1,2,3], Anthony A. Hyman[1,2,3], Agnes Toth-Petroczy[1,2,4]*

[1]Max Planck Institute of Molecular Cell Biology and Genetics, Dresden 01307, Germany

[2]Center for Systems Biology Dresden, Dresden, 01307 Germany

[3]Current address: Department of Biology, College of Science, University of Sulaimani, Iraq

[4]Cluster of Excellence Physics of Life, TU Dresden, 01062 Dresden, Germany

*Corresponding author: Agnes Toth-Petroczy, toth-petroczy@mpi-cbg.de

#Equal contribution

This PDF file includes:

Supplementary Figures S1 to S17
Supplementary Table S1
Supplementary References

Supplementary Figures

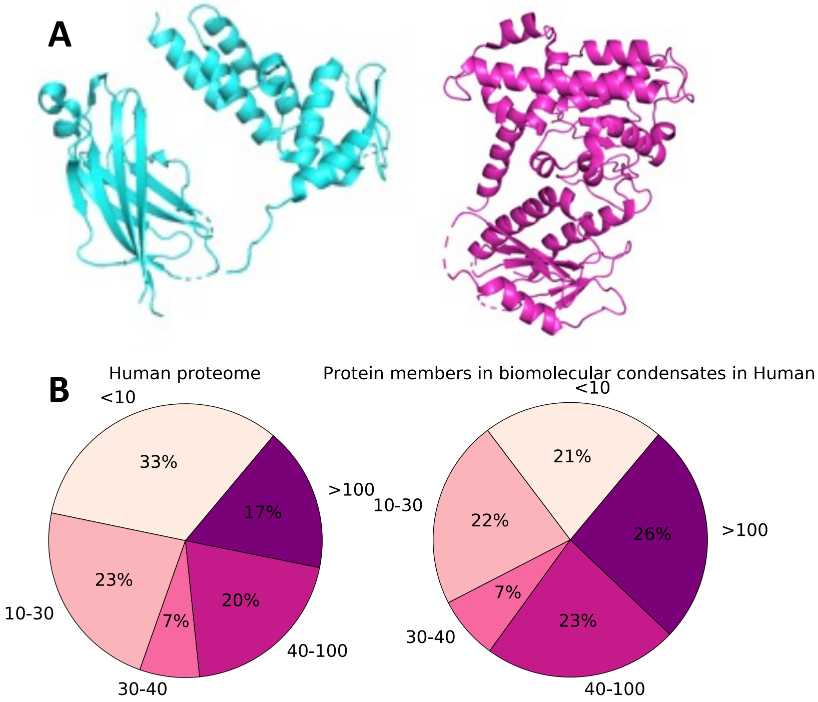

Figure S1. Examples of driver proteins in biomolecular condensates without long disordered regions.

A) Experimentally determined structure of Speckle-type POZ protein which is a driver of nuclear speckle formation and SPOP/DAXX body condensates in human (PDB accession number 3HU6, residues 28-329 were identified with 2.70 Å resolution by X-ray crystallography) (left panel). Experimentally determined structure of protein Guanine nucleotide exchange C9orf72, which is a driver protein in stress granules in human (PDB accession number 6LT0 chain C, residues 1-481 were identified with 3.20 Å resolution by electron microscopy) (right panel). **B)** Fraction of proteins with different length of disordered regions identified by IUPred[1] (with threshold 0.5) in the human proteome (left panel) and in the subset of proteins in humans which are members of biomolecular condensates according to CD-CODE (right panel). Source data are provided as a Source Data file.

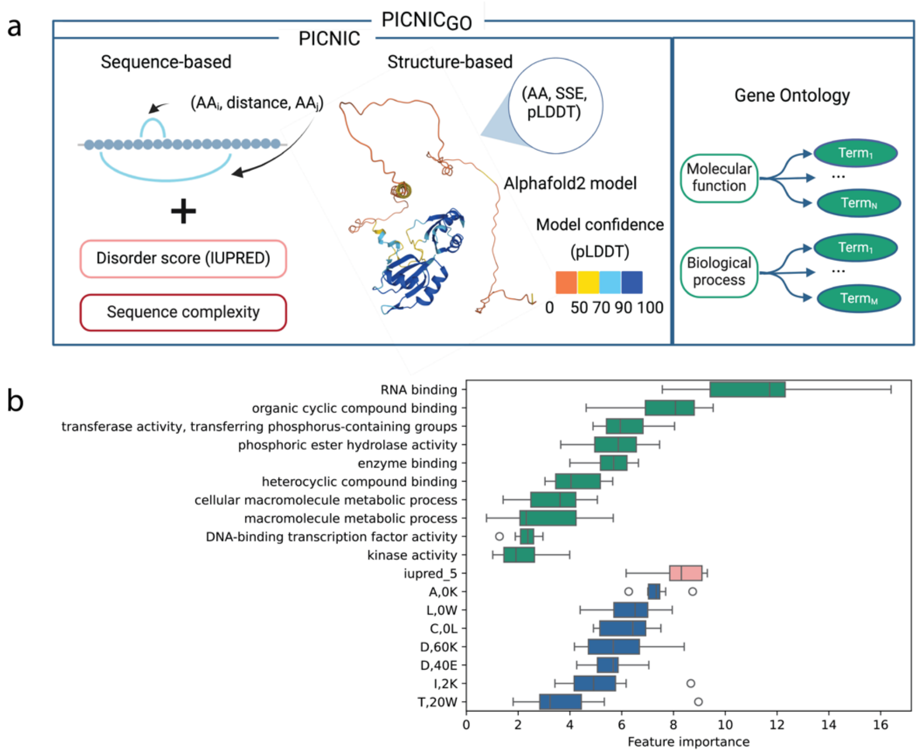

Figure S2. Development of PICNIC$_{GO}$ (Proteins Involved in CoNdensates In Cells) algorithm.

a) Sequence, structure and function-based features of PICNIC$_{GO}$. The extended version of PICNIC, named PICNIC$_{GO}$, includes gene ontology annotation features. Sequence-based features included sequence complexity, disorder score (IUPred), and features based on amino acid co-occurrences. Structure-based features based on AlphaFold2 models included the pLDDT score, a per-residue measure of local confidence on a scale from 0 – 100 (colored on the structure). We annotated the secondary structure (SSE) based on 3D protein structures using STRIDE and all possible triads in the form (AA, SSE, pLDDT) were calculated. **b)** The model including GO terms (PICNIC$_{GO}$) used only 18 features, including three types of features: 1) based on gene ontology (in green), 2) disorder (pink), 3) distance-based (blue). The most important features included RNA-binding, disorder and co-occurrences of charged and hydrophobic residues. The feature importance is consistent across different folds of cross-validation (values across 10 folds are shown, the box shows the quartiles of the dataset, *where first black* horizontal line of the rectangle shape is first quartile or 25% the *second black* horizontal line of the rectangle shape is second quartile or 50% or median, the *third black* horizontal line of the rectangle shape is third quartile or 75%. The whiskers extend to points that lie within 1.5 IQRs (interquartile range) of the lower and upper quartile, and then observations that fall outside this range are outliers and displayed as circles). Source data are provided as a Source Data file.

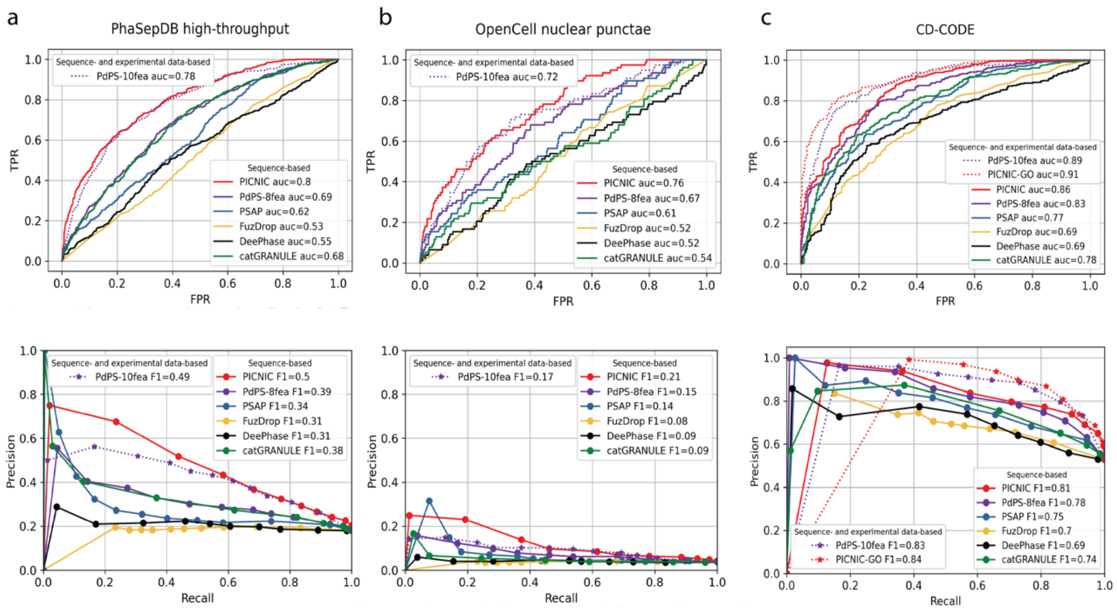

Figure S3. PICNIC models have the best performance in predicting condensate forming proteins.

Comparison of sequence-based predictors (lines, PICNIC, PdPS-8fea[2], PSAP[3], FuzDrop[4], DeePhase[5] and catGRANULE[6]) and predictors using experimental data (dotted lines) as features to predict protein condensates. Specifically, PdPS-10-fea uses phosphorylation sites and immunofluorescent microscopy images of the proteins, $\text{PICNIC}_{\text{GO}}$ uses GO-terms as features. True positive rates (TPR) and false positive rates (FPR) are shown on top panels and precision-recall curves are shown at the bottom panels. **a)** Test dataset from PhaSepDB high-throughput retrieved from[2] (441 positive and 1998 negative examples, excluding proteins that were part of the PICNIC training set), **b)** test dataset from OpenCell[7] (78 positive and 1998 negative examples excluding proteins that were part of the PICNIC training set), **c)** test dataset from the current study based on CD-CODE[8] (338 positive and 299 negative examples that were not part of the training set). PICNIC outperforms sequence-based predictors even on the test set that includes training data of previously published predictors, that may inflate their performance. Source data are provided as a Source Data file.

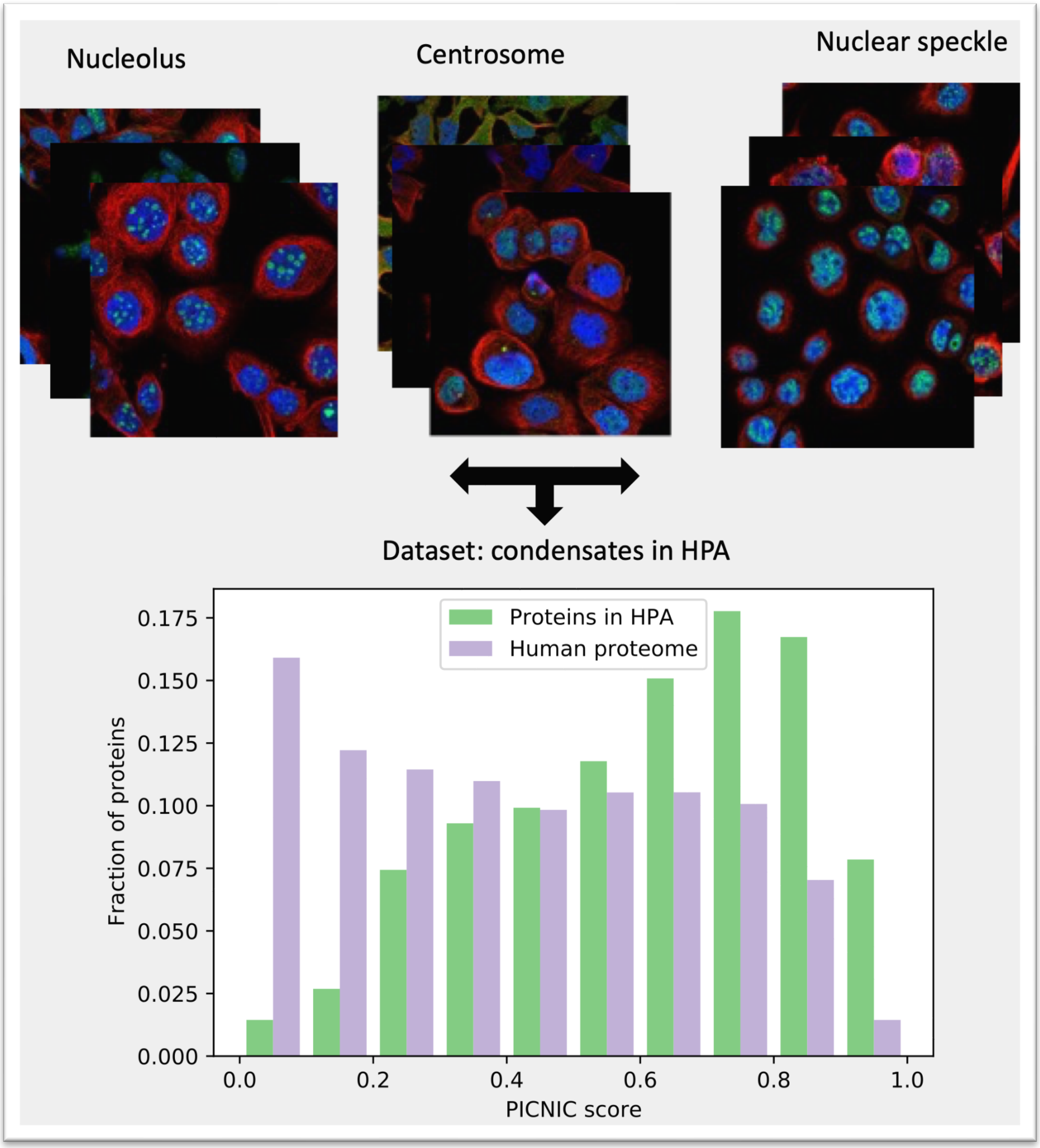

Figure S4. Distribution of PICNIC scores for proteins in three different condensates based on Human Protein Atlas (HPA, proteinatlas.org) (N=484, showed in green) and canonical proteins in human (N= 17361, showed in violet)[9]. Images and annotations for proteins were retrieved for three types of cellular localization: Nucleolus, Centrosome and Nuclear speckles. Only the proteins supported by experimental evidence were taken into account. All proteins from our training dataset were removed from this analysis. Source data are provided as a Source Data file.

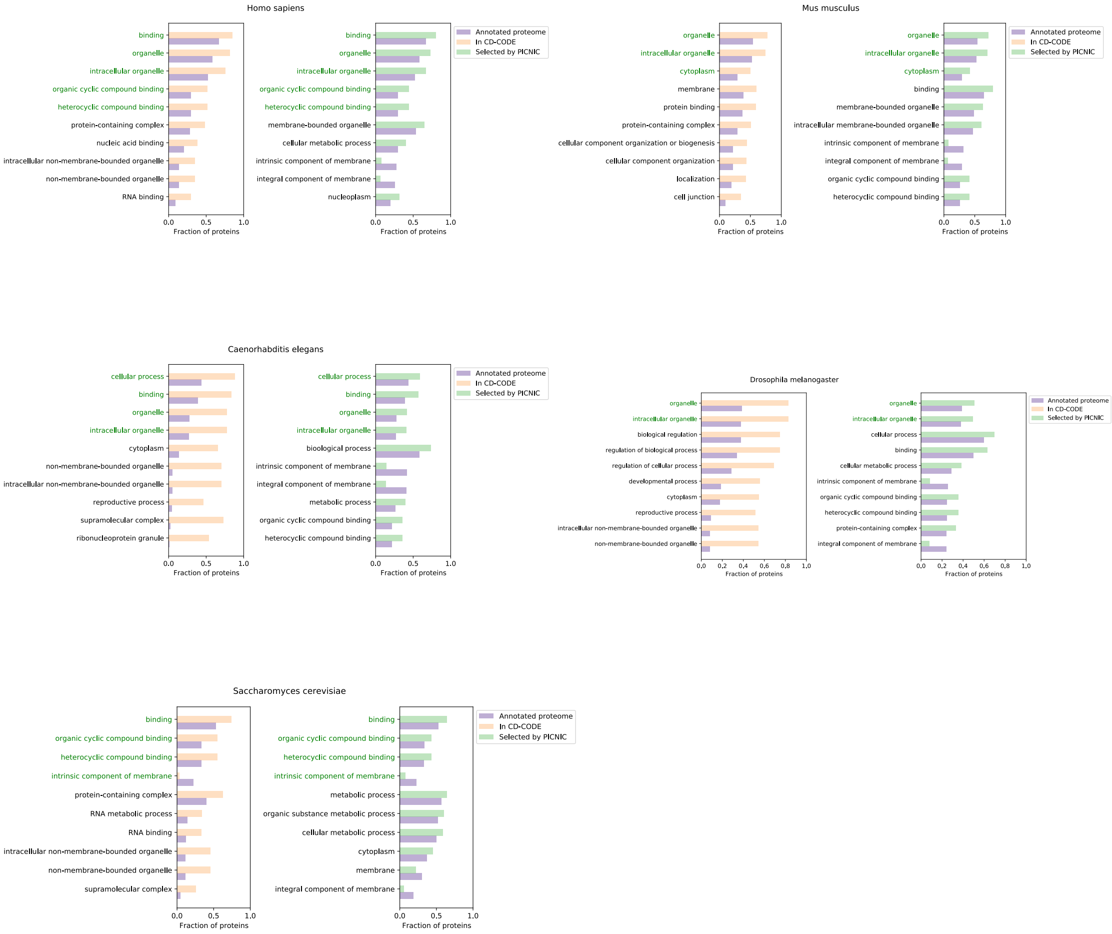

Figure S5. PICNIC detects properties of proteins in biomolecular condensates captured by Gene Ontology (GO) annotations for different species. Top 10 Gene Ontology features are displayed for each specie sorted by absolute value of difference in corresponding distributions (all annotated proteome vs. proteins in CD-CODE[8] on the left panel, all annotated proteome vs. proteins selected as positive by PICNIC on the right panel). Common features (with most divergent distributions between all proteins and proteins in biomolecular condensates, captured by PICNIC model) are shown in green color. Only species with sufficient number of proteins detected in biomolecular condensates (presented in CD-CODE) were chosen for the analysis. Source data are provided as a Source Data file.

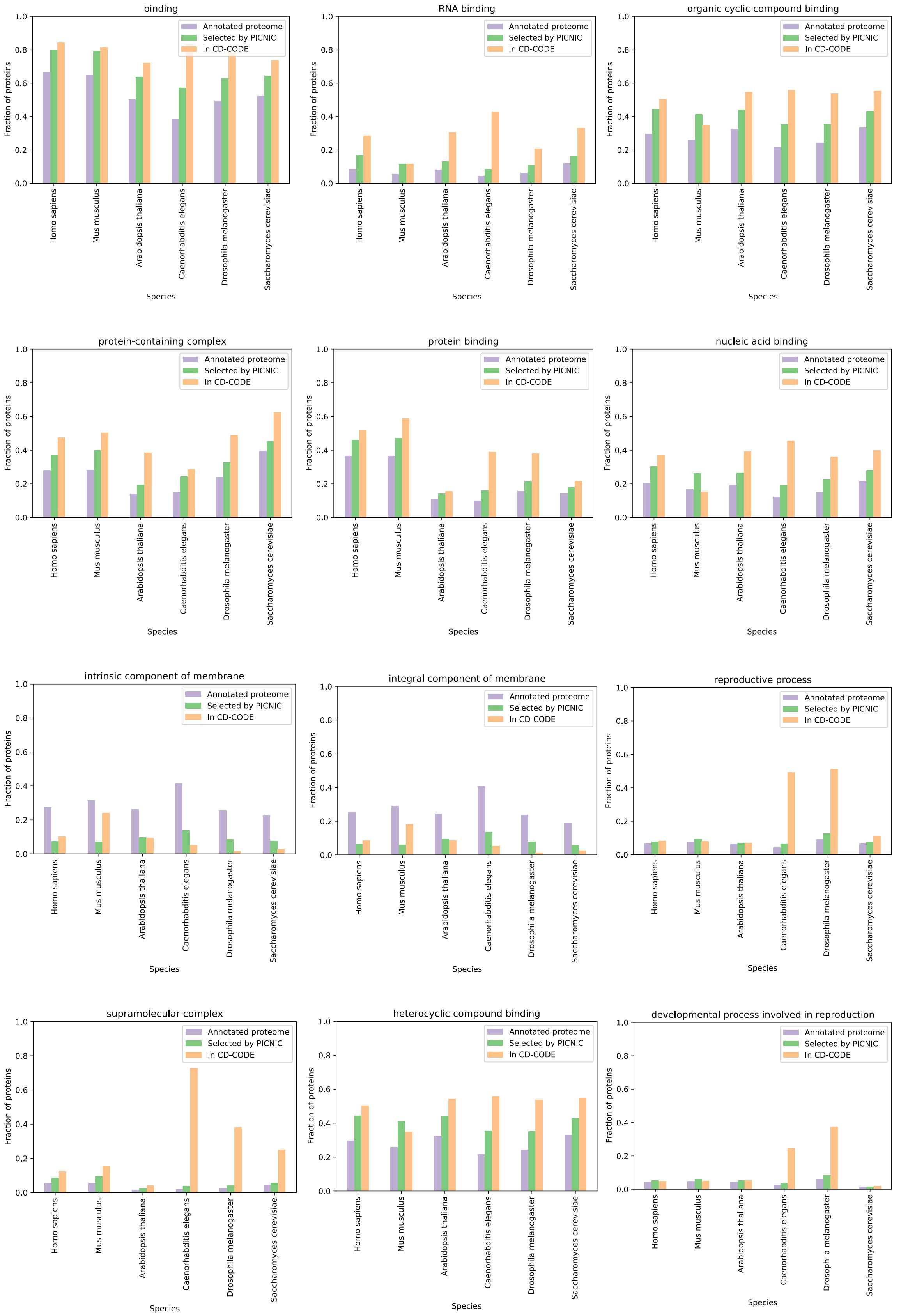
binding
RNA binding
organic cyclic compound binding
protein-containing complex
protein binding
nucleic acid binding
intrinsic component of membrane
integral component of membrane
reproductive process
supramolecular complex
heterocyclic compound binding
developmental process involved in reproduction
Annotated proteome
Selected by PICNIC
In CD-CODE
Fraction of proteins
Species
Homo sapiens
Mus musculus
Arabidopsis thaliana
Caenorhabditis elegans
Drosophila melanogaster
Saccharomyces cerevisiae
0.0
0.2
0.4
0.6
0.8
1.0

Figure S6. Difference in GO annotation distributions for proteins in biomolecular condensates and all proteins (grouped by GO features and compared to proteins detected by PICNIC). Source data are provided as a Source Data file.

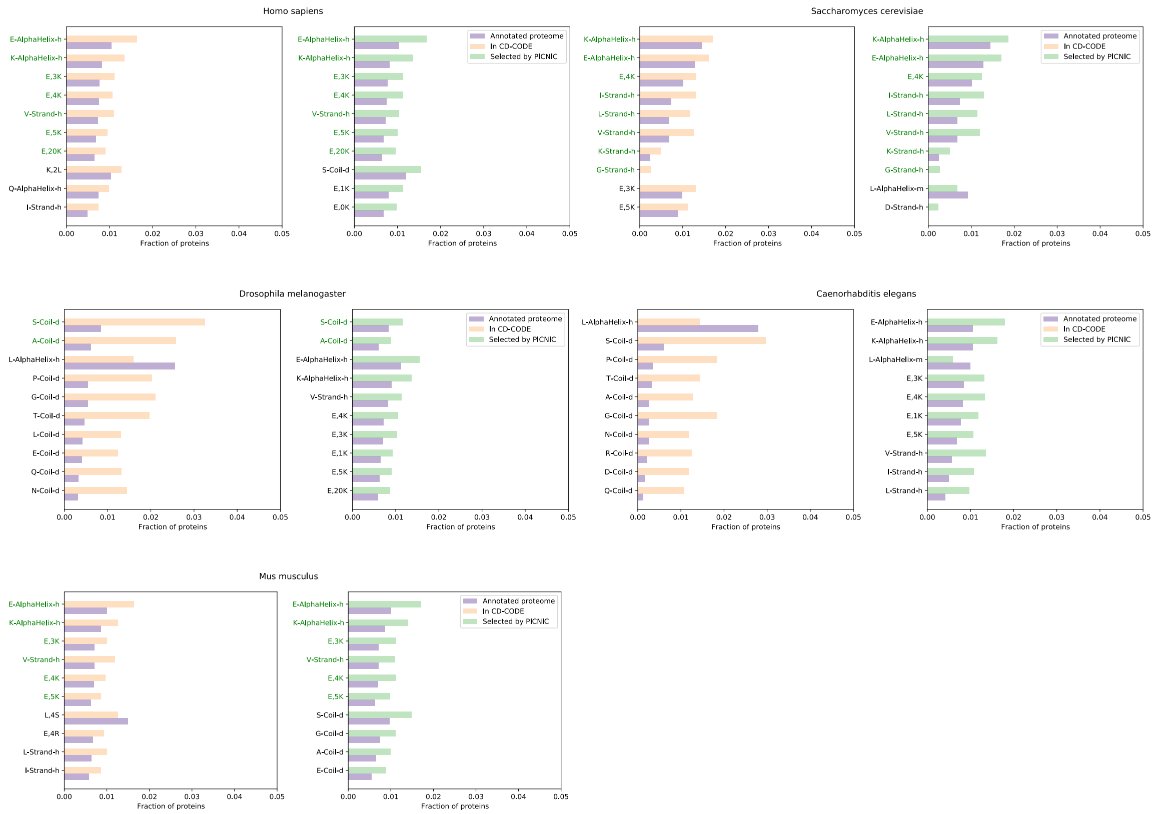

Figure S7. PICNIC$_{GO}$ detects properties of proteins in biomolecular condensates captured by distance-based and structure-based features for different species.

Top 10 features (with exclusion of disorder and complexity score) are displayed for each specie sorted by absolute value of difference in corresponding distributions (all annotated proteome vs. proteins in CD-CODE on the left panel; all annotated proteome vs. proteins selected as positive by PICNIC$_{GO}$ on the right panel). Common features (with most divergent distributions between all proteins and proteins in biomolecular condensates, captured by PICNIC$_{GO}$ model) are shown in green color. Only species with sufficient number of proteins detected in biomolecular condensates (presented in CD-CODE) were chosen for the analysis. Source data are provided as a Source Data file.

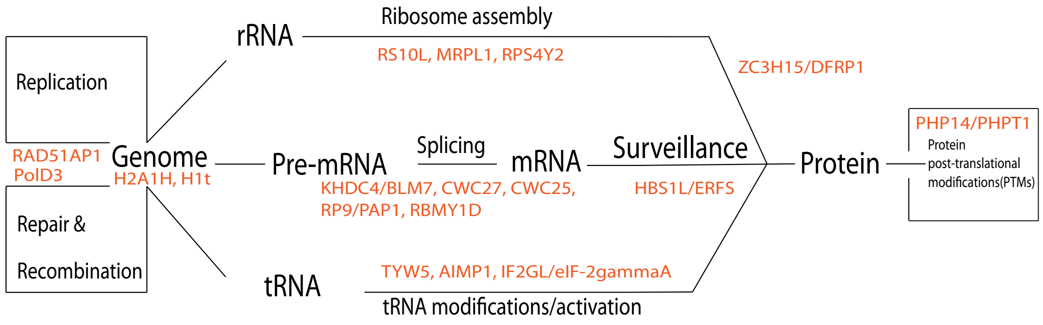

Figure S8. Molecular functions and properties of the proteins in the experimental validation dataset.

Molecular scale functions of the tested 24 proteins span a wide range from transcription, translation to post-translational modifications. The length of proteins varies between 125-684 amino acids (**Dataset S2**).

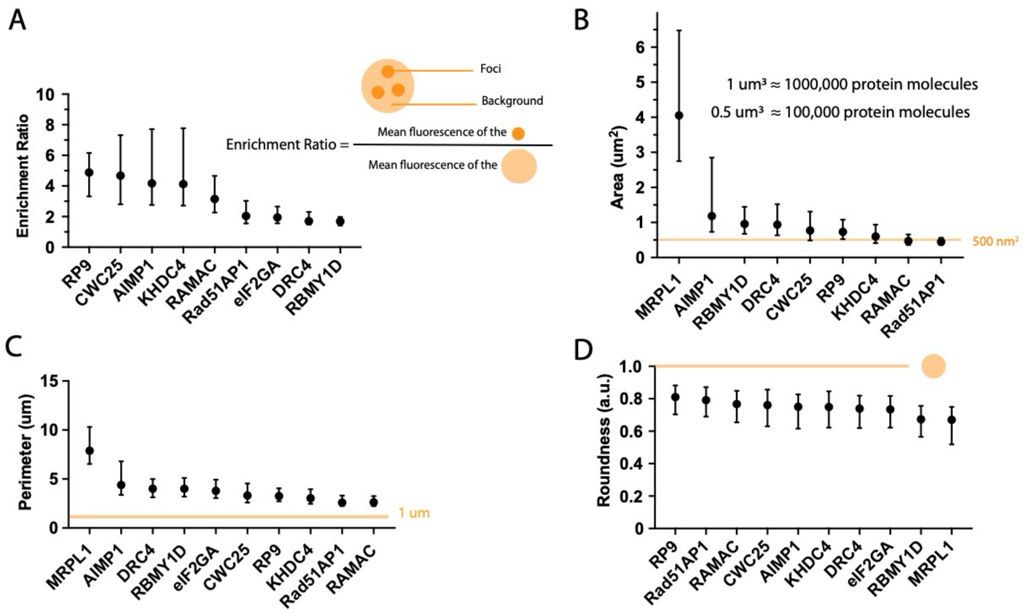

Figure S9. Quantification of fluorescent images to define condensates.

A) Foci were defined based on enrichment in fluorescent intensity, i.e., the intensity ratio inside relative to outside of the foci is greater than one.

We measured simple characteristics from fluorescent microscopy images: area (B) and perimeter (C), informing on the size and the typical number of proteins in a foci); and shape (roundness, D). We consider foci as condensates above a diameter of 350 nm (distance between two furthest pixels in one condensate), that is well above the diffraction limit. This would correspond to ~1µm perimeter assuming a round shape. Median and interquartile ranges are shown. Source data are provided as a Source Data file.

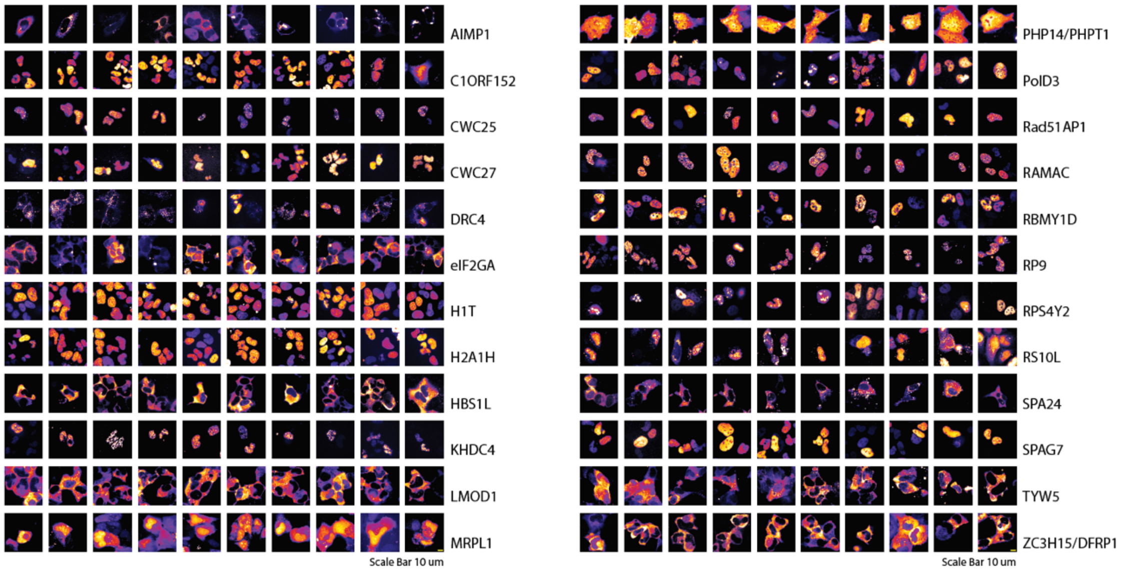

Figure S10. Gallery of cells expressing the 24 proteins tested which were predicted to form condensates (positive predictions).

For each protein tested 10 different images showing one to many cells in the same field of view consistently demonstrating the presence of condensates for 21 out of 24 proteins Interestingly, some proteins showed localization to more than one type/site of condensate. For example, DRC4: nuclear and cytoplasmic; MRPL1: nuclear and cytoplasmic bodies; RS10L: cytoplasmic bodies/ filaments as well as nucleolar localization.

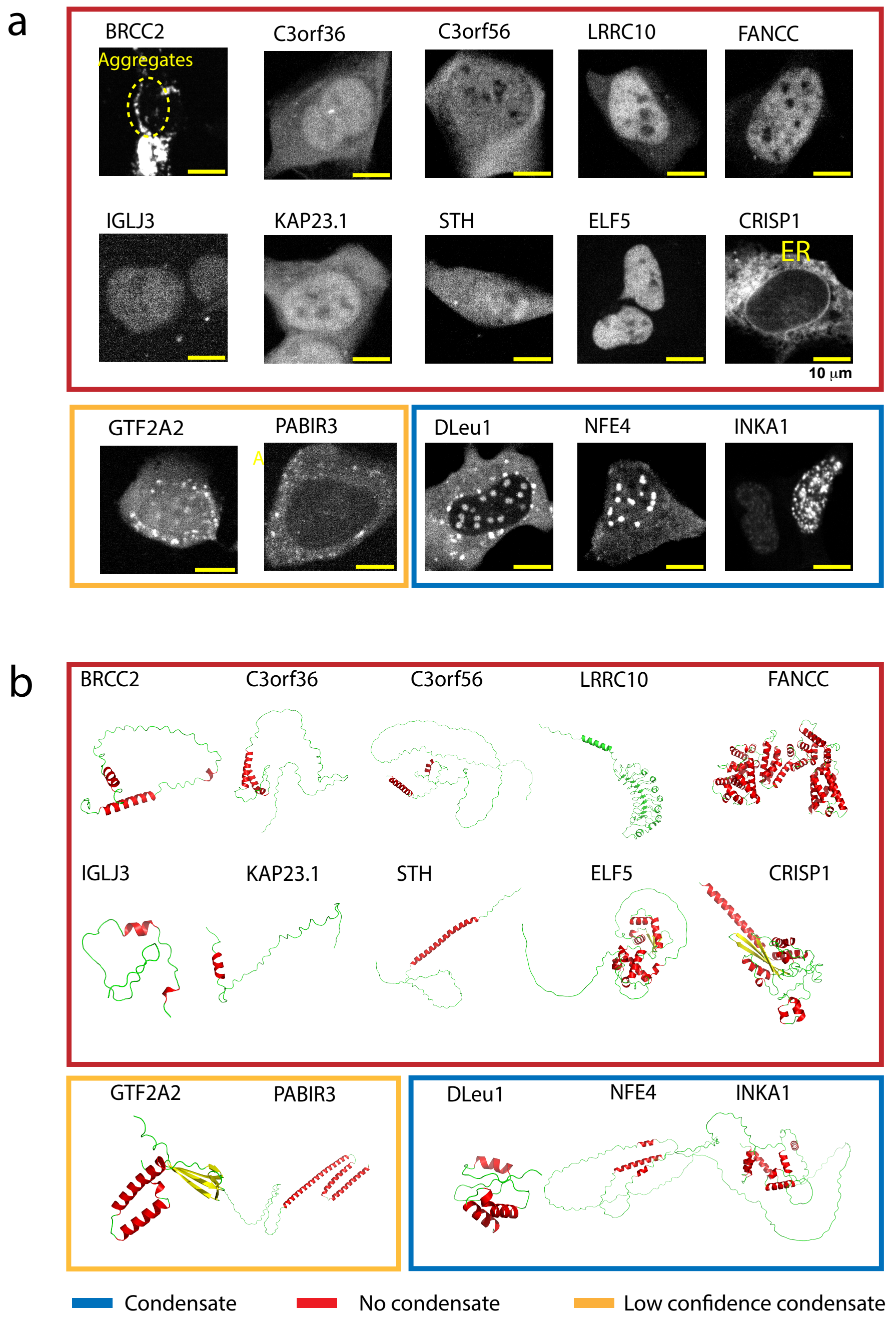

Figure S11. Most (10 out of 15) proteins predicted not to form condensates indeed do not form detectable foci.

a) Representative images of the U2OS cells expressing the tested proteins tagged with a fluorescent protein (iRFP). All images are scaled to the scale bar 10 μm (shown as yellow bars). We found 10 out of the 15 tested proteins did not form condensates, while two proteins GTFA2 and PABIR3 formed low confidence condensates in the minority of cells (yellow square), and 3 proteins formed high confidence condensates (blue squares). Images are provided as **Dataset S4**.

b) Wide range of structural motifs covered in the test proteins; AlphaFold2 structural models of the proteins are colored according to secondary structures (AF2 pdb structures are provided as **Dataset S6**). Many of the tested proteins have large IDRs and yet do not form condensates.

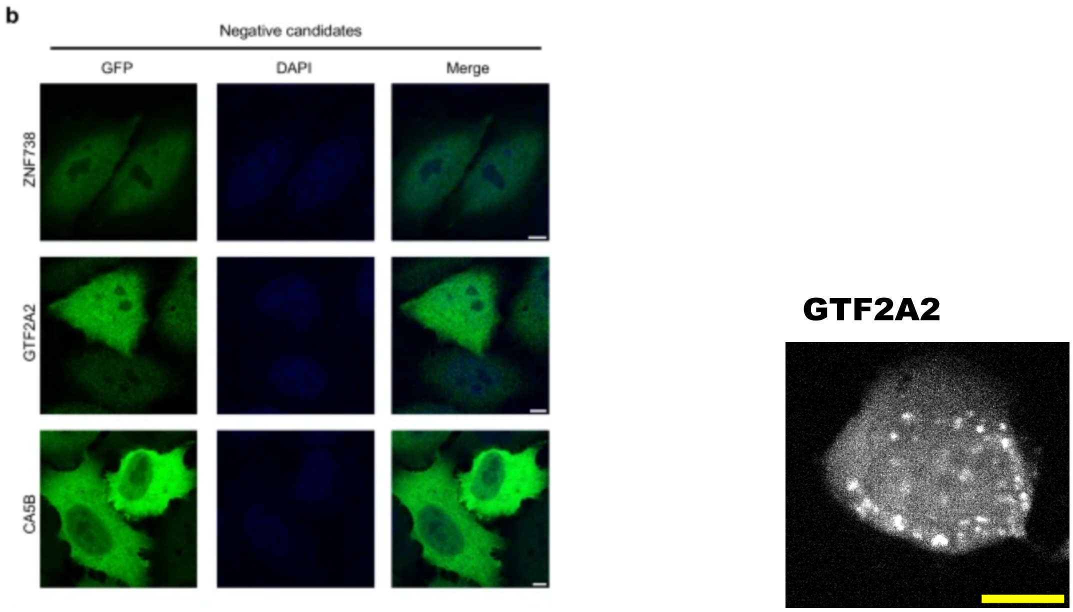

Figure S12. Cell-type specific condensate formation.
Previously reported condensate negative protein, GTF2A2 expressed in HeLa cells tagged with GFP (left panel, Figure 5b middle from Hou et al. Nature Communications 2024)[10] form low-confidence condensates in our experiments (right panel) expressed in U2OS cells and tagged with iRFP (image available as **Dataset S4**). The image is scaled to the scale bar 10 µm (shown as yellow bar).

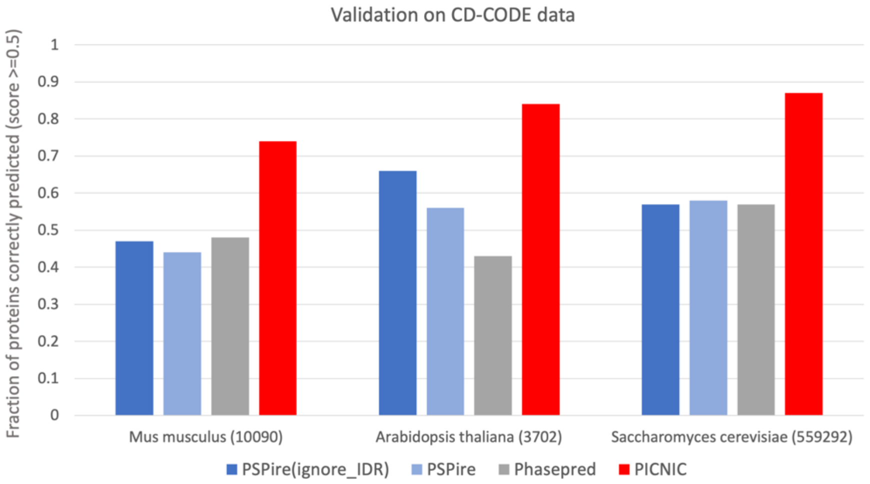

Figure S13. Performance of predictors on a set of species other than human. PICNIC performs best amongst the predictors that were possible to test, Phasepred[2] and PSPire[10], which gives two scores (PSPire and PSPire(ignore_IDR)). Other tools do not provide and/or allow computation of scores on any sequence. Data on condensate forming proteins are extracted from the CD-CODE database (*Mus musculus N* = 1644, *Arabidopsis thaliana N* =1497, *Saccharomyces cerevisiae N* = 583). Source data are provided as a Source Data file.

a)

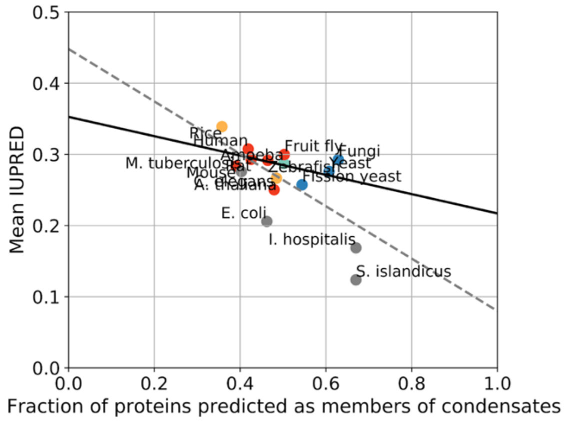

b)

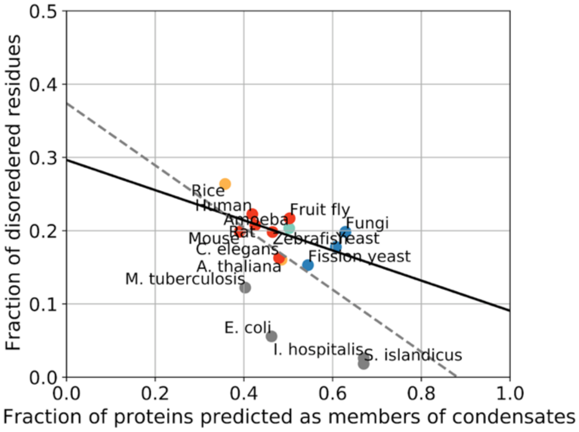

c)

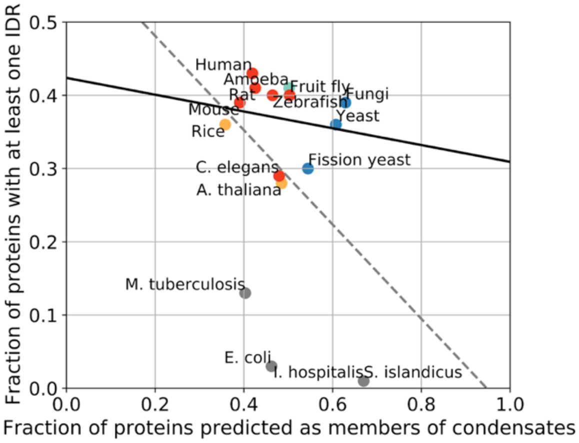

Figure S14. Overall disorder of a proteome does not correlate with the fraction of predicted condensate forming proteins.

We used three different metrics to quantify disorder of 16 proteomes and computed Pearson correlation with fraction of proteins predicted to be members of condensates by PICNIC: **a)** fraction of proteins with at least one IDR (>40 aa), R^2=0.03 (solid line, without grey dots), R^2=0.17 (dashed line, all dots), **b)** Mean of the mean IUPred score of all proteins, R^2=0.22 (solid line, without grey dots), R^2=0.44 (dashed line, all dots) **c)** Mean of the fraction of disordered residues (IUPRED >=

0.5) per protein, R^2=0.30 (solid line, without grey dots), R^2=0.34 (dashed line, all dots). Source data are provided as a Source Data file.

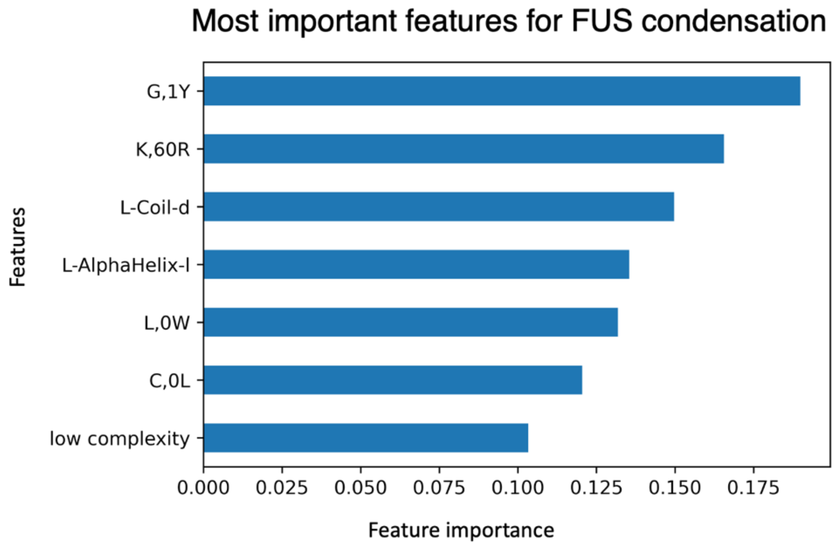

Figure S15. The top features picked by PICNIC to predict FUS (Uniprot ID: FUS_HUMAN) condensation. Source data are provided as a Source Data file.

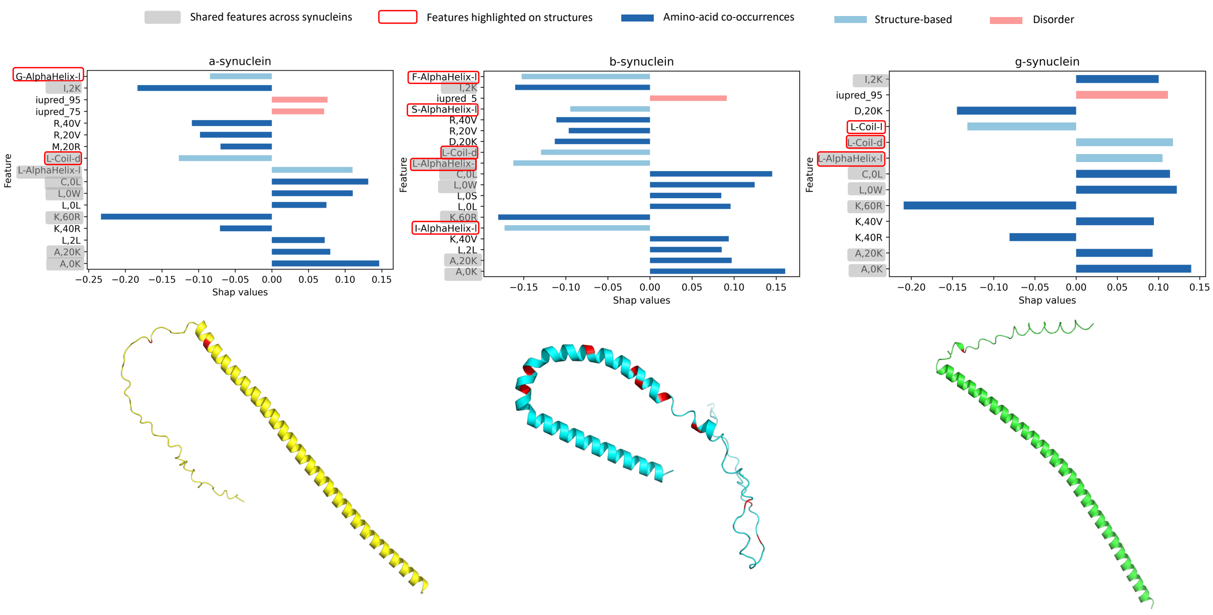

Figure S16. **The top features picked by PICNIC regarding the synuclein protein family: alpha-, beta- and gamma-synuclein.** Threshold for absolute SHAP (SHapley Additive exPlanations) values displayed was 0.07, 0.08, 0.08, respectively). Negative SHAP values mean that the feature is present, while positive values mean that the feature is absent in a given protein. There are many shared features that contribute to the prediction for all three synucleins (grey). The structure-based features (red) were mapped on the respective 3D models (AlphaFold2) of the proteins. Source data are provided as a Source Data file.

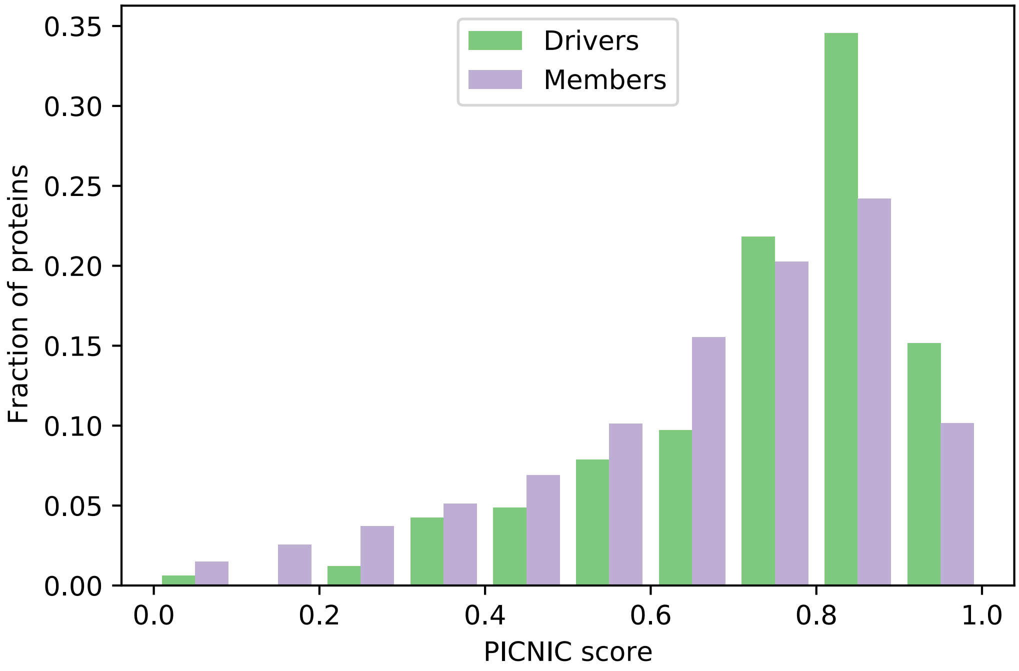

Figure S17. Distribution of PICNIC scores for proteins annotated as drivers (*N*=165 shown in green) and members (*N*=8074, shown in violet) in CD-CODE across 14 species. Source data are provided as a Source Data file.

Supplementary Tables

Supplementary Table 1. List of proteins that contain mutations (point mutations or deletions) and their liquid-liquid phase separation (LLPS) properties (protein sequences are provided as **Dataset S5**).

**name**	**LLPS**	**gene**	**PICNIC**	**type of mutation**	**PubMed ID**
sod1	yes	sod1	0.8100940564065315	wt	36416085
G85R	yes	sod1	0.8387347877510064	point mutations	36416085
I113T	yes	sod1	0.8100940564065315	point mutations	36416085
G37R	no	sod1	0.8297468021417307	point mutations	36416085
cbx2	yes	cbx2	0.8091539978408043	wt	31171700
cbx2_13	no	cbx2	0.6554493794706464	point mutations	31171700
cbx2_23	no	cbx2	0.6228464034932841	point mutations	31171700
cbx2_16	no-yes (defects)	cbx2	0.6035792935089319	point mutations	31171700
cbx2_10	no-yes (defects)	cbx2	0.6898040289269018	point mutations	31171700
cbx2_dea	yes	cbx2	0.8109040163434185	point mutations	31171700
hnRNPA1_noidr	no	hnRNPA1	0.9098013063765105	deletion	26412307
hnRNPA1	yes	hnRNPA1	0.9201123054130387	wt	26412307
fmr1	yes	fmr1	0.8963611531078023	wt	30765518
fmr1_nolcr	no	fmr1	0.8189975423279743	deletion	30765518
cgas	yes	cgas	0.8398338477242777	deletion	29976794
cgas_del	no	cgas	0.6530765323971761	deletion	29976794
fib1	yes	fib1	0.9366168221837954	deletion	27212236
fib1_mut	no	fib1	0.8760152480967373	deletion	27212236
laf1	yes	laf1	0.8740628322878754	wt	26015579
laf1_del	no	laf1	0.8253481317961218	deletion	26015579
tau	yes	tau	0.8158427467627462	wt	29472250
tau_mut	no	tau	0.7984753587950086	deletion + point mutations	29472250
tardbp	yes	tardbp	0.8786224374351072	wt	27545621
tardbp_del	no	tardbp	0.5940628364337122	deletion	27545621
edc3	yes	edc3	0.6296964850131994	wt	28472520
edc3_mut	no	edc3	0.7029606052376794	deletion	28472520
edc3_noidr	no	edc3	0.5329281825693932	deletion	28472520

Supplementary References

1. Mészáros, B., Erdos, G. & Dosztányi, Z. IUPred2A: context-dependent prediction of protein disorder as a function of redox state and protein binding. *Nucleic Acids Res.* **46**, W329–W337 (2018).
2. Chen, Z. *et al.* Screening membraneless organelle participants with machine-learning models that integrate multimodal features. *Proc. Natl. Acad. Sci. U. S. A.* **119**, e2115369119 (2022).
3. van Mierlo, G. *et al.* Predicting protein condensate formation using machine learning. *Cell Rep.* **34**, 108705 (2021).
4. Hardenberg, M., Horvath, A., Ambrus, V., Fuxreiter, M. & Vendruscolo, M. Widespread occurrence of the droplet state of proteins in the human proteome. *Proc. Natl. Acad. Sci. U. S. A.* **117**, 33254–33262 (2021).
5. Saar, K. L. *et al.* Learning the molecular grammar of protein condensates from sequence determinants and embeddings. *Proc. Natl. Acad. Sci. U. S. A.* **118**, (2021).
6. Bolognesi, B. *et al.* A Concentration-Dependent Liquid Phase Separation Can Cause Toxicity upon Increased Protein Expression. *Cell Rep.* **16**, 222–231 (2016).
7. Cho, N. H. *et al.* OpenCell: Endogenous tagging for the cartography of human cellular organization. *Science* **375**, eabi6983 (2022).
8. Rostam, N. *et al.* CD-CODE: crowdsourcing condensate database and encyclopedia. *Nat. Methods* (2023) doi:10.1038/s41592-023-01831-0.
9. Karlsson, M. *et al.* A single-cell type transcriptomics map of human tissues. *Sci Adv* **7**, (2021).
10. Hou, S. *et al.* Machine learning predictor PSPire screens for phase-separating proteins lacking intrinsically disordered regions. *Nat. Commun.* **15**, 2147 (2024).
