# Supplementary figures and images for: PICNIC accurately predicts condensate-forming proteins regardless of their structural disorder across organisms (part 2 of 3)
Source: Nat Commun. 2024 Dec 11;15:10668. doi: 10.1038/s41467-024-55089-x (PMC11634905; doi:10.1038/s41467-024-55089-x)

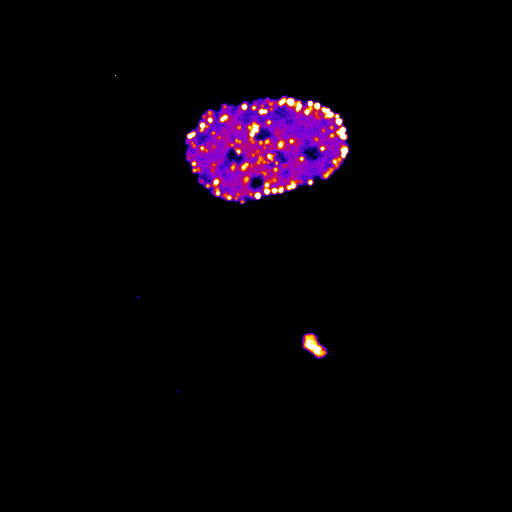

Supplement: Supplementary file 5 — Dataset S4 [file 41467_2024_55089_MOESM5_ESM.zip › Dataset_S4_representative_images/FigureS10_Representative Images/RAMAC/6.tif]

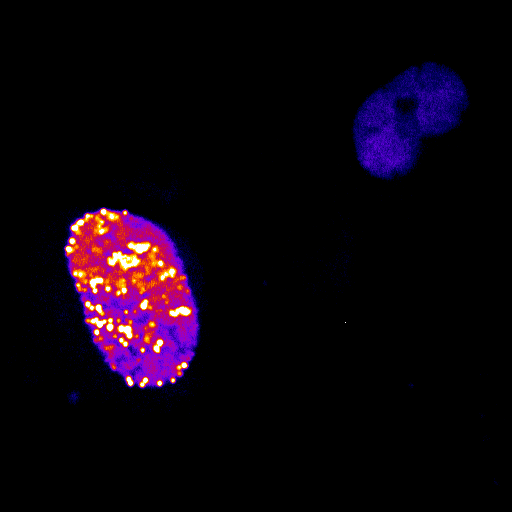

Supplement: Supplementary file 5 — Dataset S4 [file 41467_2024_55089_MOESM5_ESM.zip › Dataset_S4_representative_images/FigureS10_Representative Images/RAMAC/7.tif]

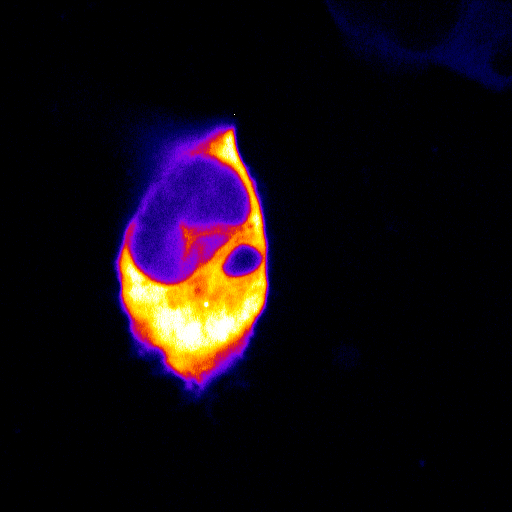

Supplement: Supplementary file 5 — Dataset S4 [file 41467_2024_55089_MOESM5_ESM.zip › Dataset_S4_representative_images/FigureS10_Representative Images/ZC3H15/10.tif]

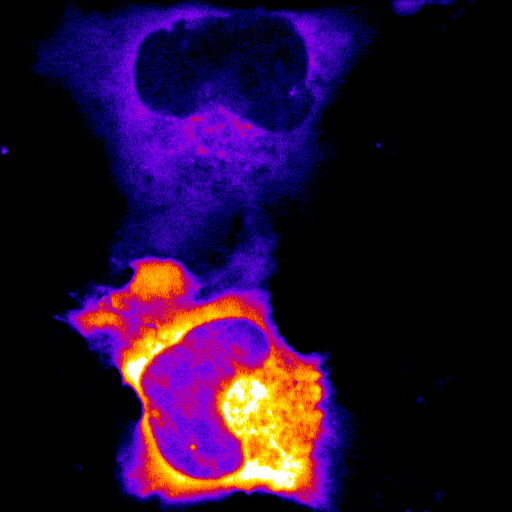

Supplement: Supplementary file 5 — Dataset S4 [file 41467_2024_55089_MOESM5_ESM.zip › Dataset_S4_representative_images/FigureS10_Representative Images/ZC3H15/11.tif]

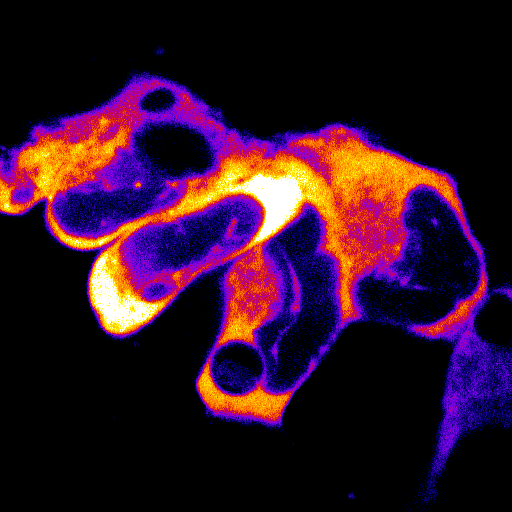

Supplement: Supplementary file 5 — Dataset S4 [file 41467_2024_55089_MOESM5_ESM.zip › Dataset_S4_representative_images/FigureS10_Representative Images/ZC3H15/9.tif]

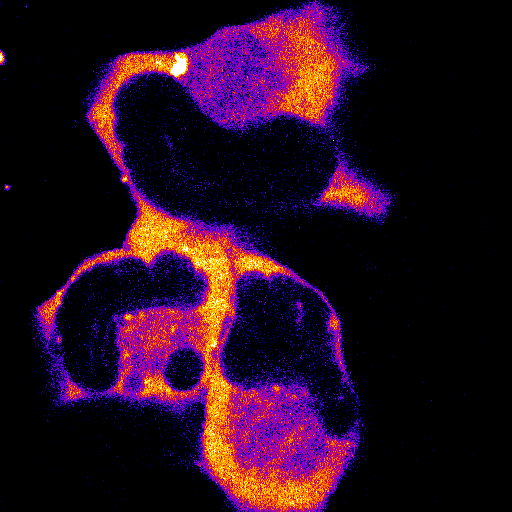

Supplement: Supplementary file 5 — Dataset S4 [file 41467_2024_55089_MOESM5_ESM.zip › Dataset_S4_representative_images/FigureS10_Representative Images/ZC3H15/8.tif]

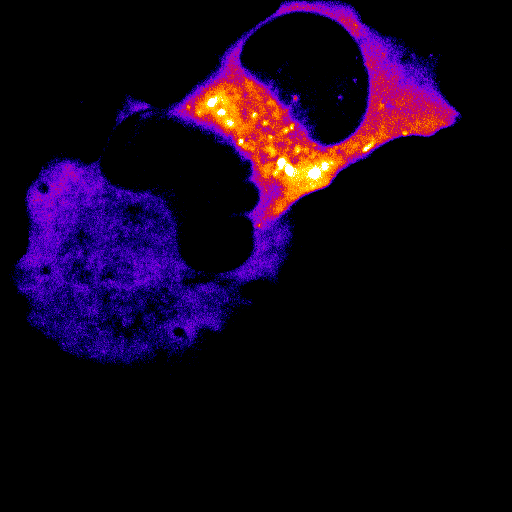

Supplement: Supplementary file 5 — Dataset S4 [file 41467_2024_55089_MOESM5_ESM.zip › Dataset_S4_representative_images/FigureS10_Representative Images/ZC3H15/3.tif]

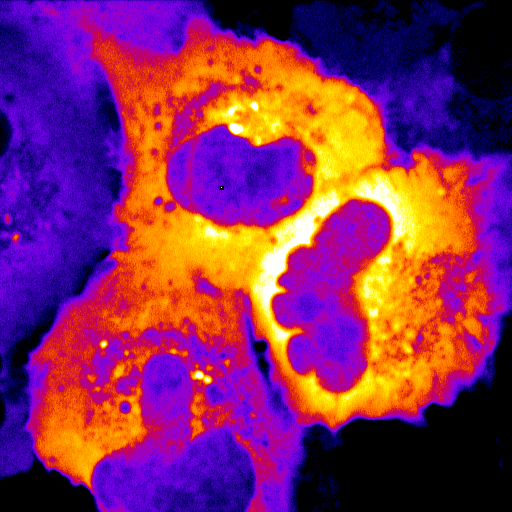

Supplement: Supplementary file 5 — Dataset S4 [file 41467_2024_55089_MOESM5_ESM.zip › Dataset_S4_representative_images/FigureS10_Representative Images/ZC3H15/2.tif]

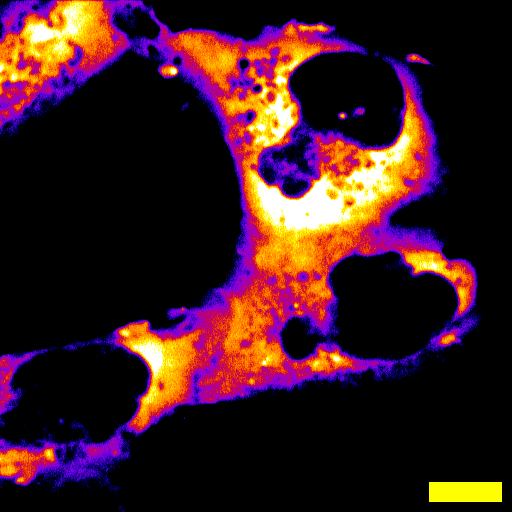

Supplement: Supplementary file 5 — Dataset S4 [file 41467_2024_55089_MOESM5_ESM.zip › Dataset_S4_representative_images/FigureS10_Representative Images/ZC3H15/1.tif]

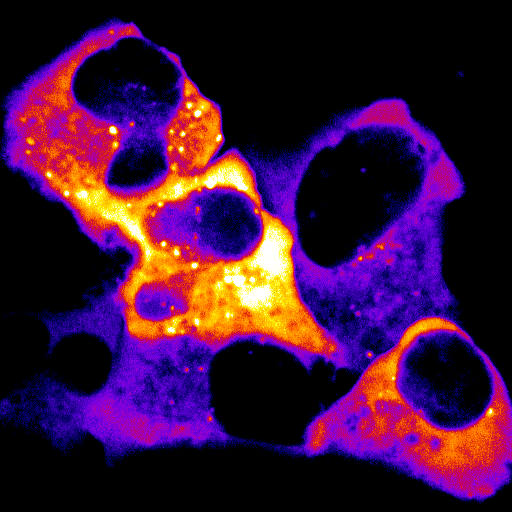

Supplement: Supplementary file 5 — Dataset S4 [file 41467_2024_55089_MOESM5_ESM.zip › Dataset_S4_representative_images/FigureS10_Representative Images/ZC3H15/5.tif]

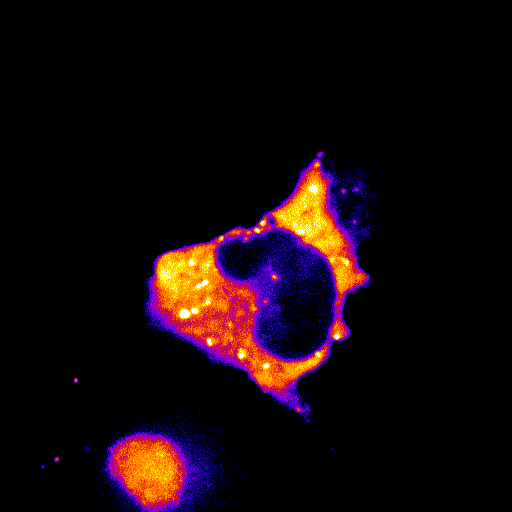

Supplement: Supplementary file 5 — Dataset S4 [file 41467_2024_55089_MOESM5_ESM.zip › Dataset_S4_representative_images/FigureS10_Representative Images/ZC3H15/4.tif]

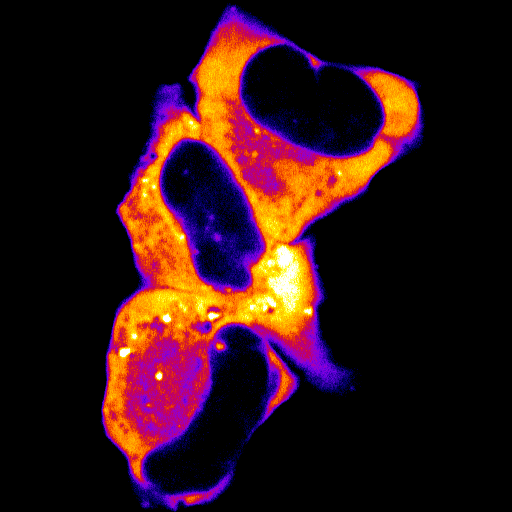

Supplement: Supplementary file 5 — Dataset S4 [file 41467_2024_55089_MOESM5_ESM.zip › Dataset_S4_representative_images/FigureS10_Representative Images/ZC3H15/6.tif]

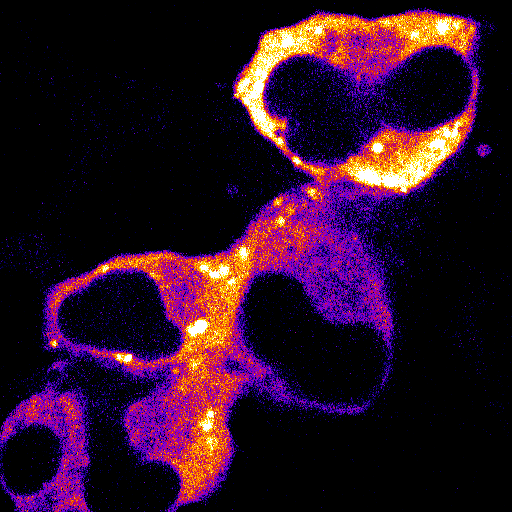

Supplement: Supplementary file 5 — Dataset S4 [file 41467_2024_55089_MOESM5_ESM.zip › Dataset_S4_representative_images/FigureS10_Representative Images/ZC3H15/7.tif]

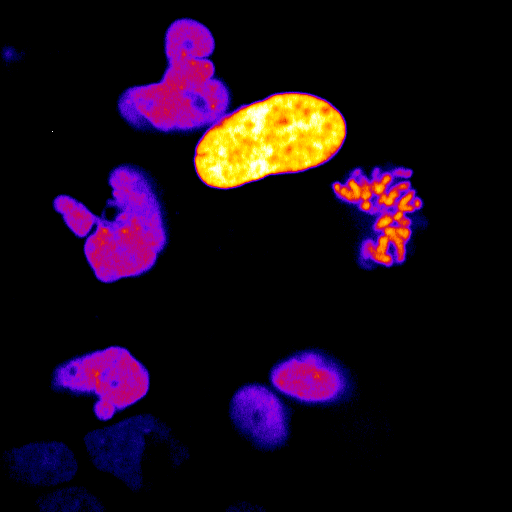

Supplement: Supplementary file 5 — Dataset S4 [file 41467_2024_55089_MOESM5_ESM.zip › Dataset_S4_representative_images/FigureS10_Representative Images/H2A1H/10.tif]

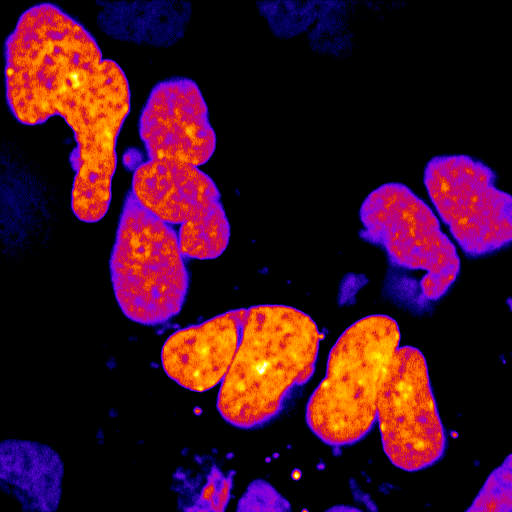

Supplement: Supplementary file 5 — Dataset S4 [file 41467_2024_55089_MOESM5_ESM.zip › Dataset_S4_representative_images/FigureS10_Representative Images/H2A1H/9.tif]

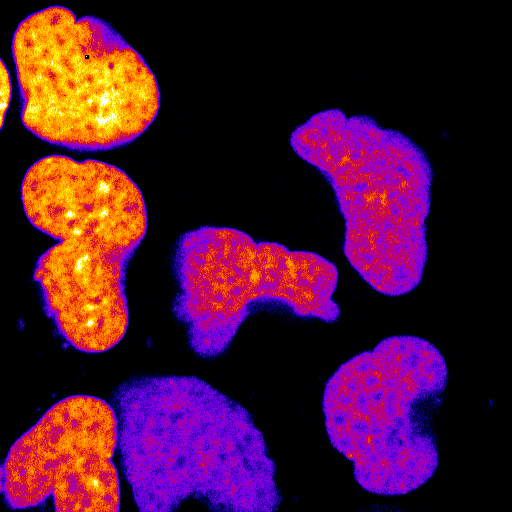

Supplement: Supplementary file 5 — Dataset S4 [file 41467_2024_55089_MOESM5_ESM.zip › Dataset_S4_representative_images/FigureS10_Representative Images/H2A1H/8.tif]

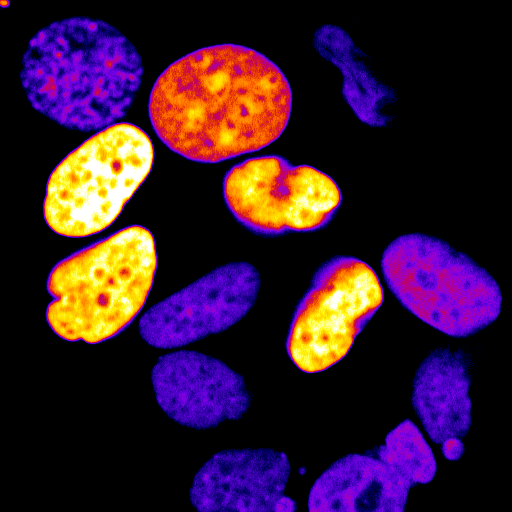

Supplement: Supplementary file 5 — Dataset S4 [file 41467_2024_55089_MOESM5_ESM.zip › Dataset_S4_representative_images/FigureS10_Representative Images/H2A1H/3.tif]

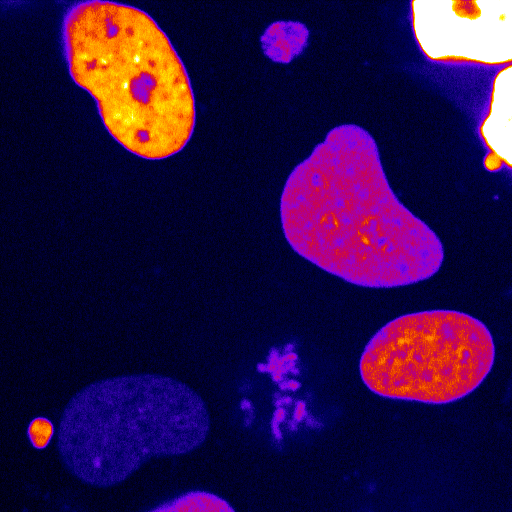

Supplement: Supplementary file 5 — Dataset S4 [file 41467_2024_55089_MOESM5_ESM.zip › Dataset_S4_representative_images/FigureS10_Representative Images/H2A1H/2.tif]

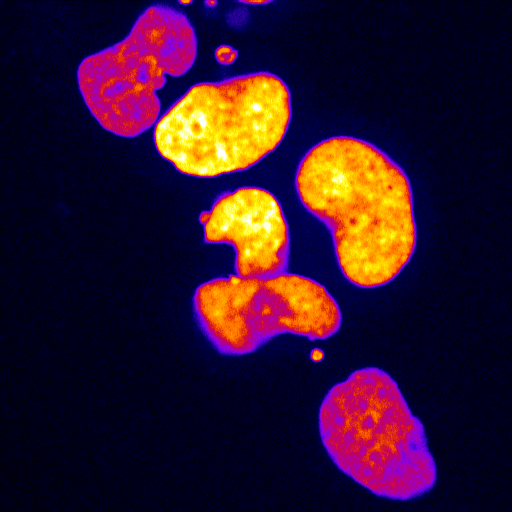

Supplement: Supplementary file 5 — Dataset S4 [file 41467_2024_55089_MOESM5_ESM.zip › Dataset_S4_representative_images/FigureS10_Representative Images/H2A1H/1.tif]

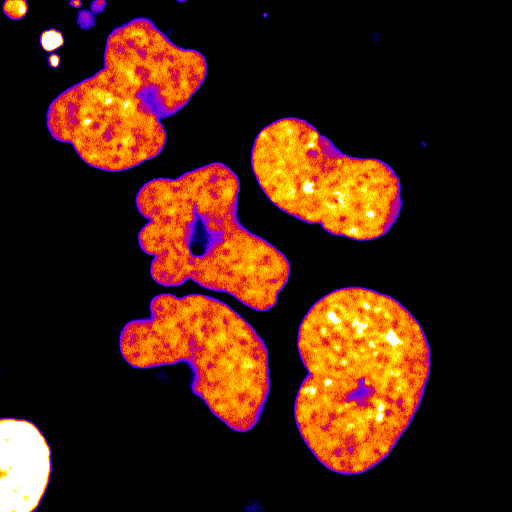

Supplement: Supplementary file 5 — Dataset S4 [file 41467_2024_55089_MOESM5_ESM.zip › Dataset_S4_representative_images/FigureS10_Representative Images/H2A1H/5.tif]

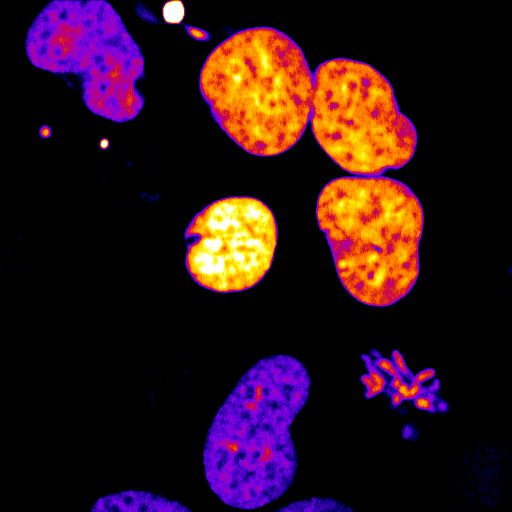

Supplement: Supplementary file 5 — Dataset S4 [file 41467_2024_55089_MOESM5_ESM.zip › Dataset_S4_representative_images/FigureS10_Representative Images/H2A1H/4.tif]

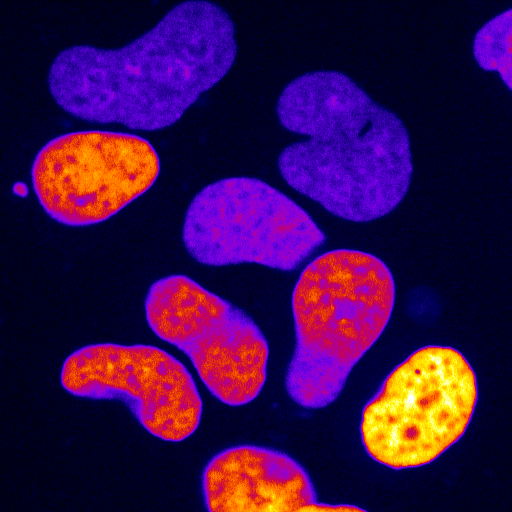

Supplement: Supplementary file 5 — Dataset S4 [file 41467_2024_55089_MOESM5_ESM.zip › Dataset_S4_representative_images/FigureS10_Representative Images/H2A1H/6.tif]

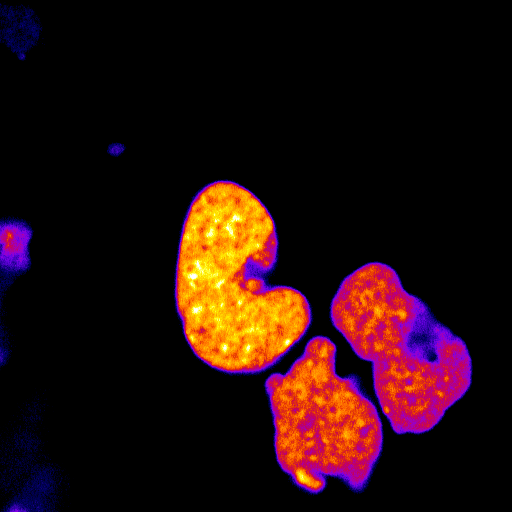

Supplement: Supplementary file 5 — Dataset S4 [file 41467_2024_55089_MOESM5_ESM.zip › Dataset_S4_representative_images/FigureS10_Representative Images/H2A1H/7.tif]

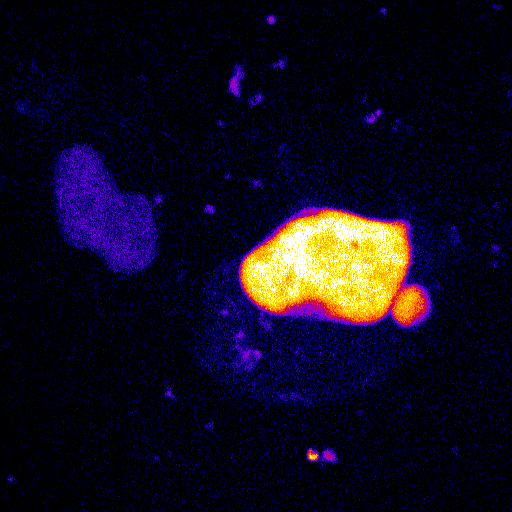

Supplement: Supplementary file 5 — Dataset S4 [file 41467_2024_55089_MOESM5_ESM.zip › Dataset_S4_representative_images/FigureS10_Representative Images/CWC27/10.tif]

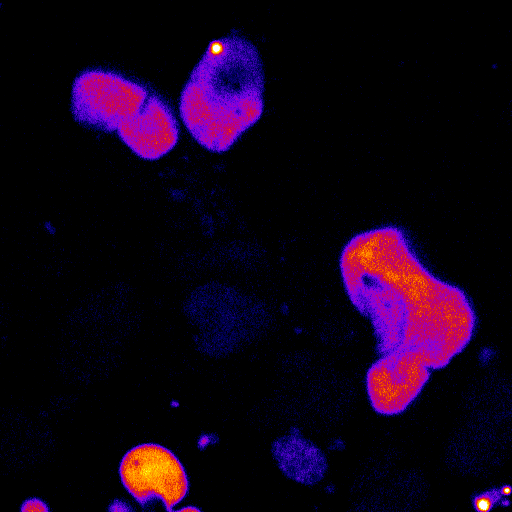

Supplement: Supplementary file 5 — Dataset S4 [file 41467_2024_55089_MOESM5_ESM.zip › Dataset_S4_representative_images/FigureS10_Representative Images/CWC27/9.tif]

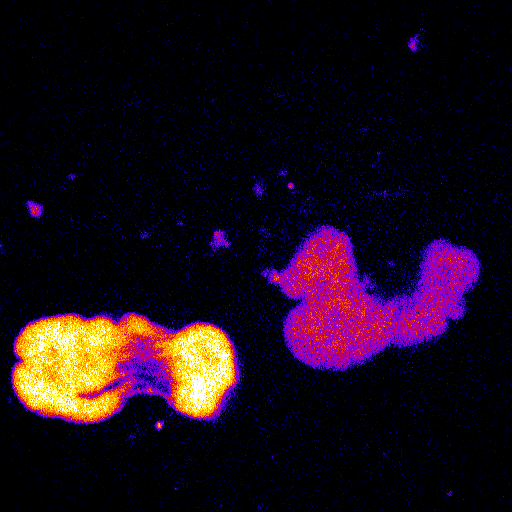

Supplement: Supplementary file 5 — Dataset S4 [file 41467_2024_55089_MOESM5_ESM.zip › Dataset_S4_representative_images/FigureS10_Representative Images/CWC27/8.tif]

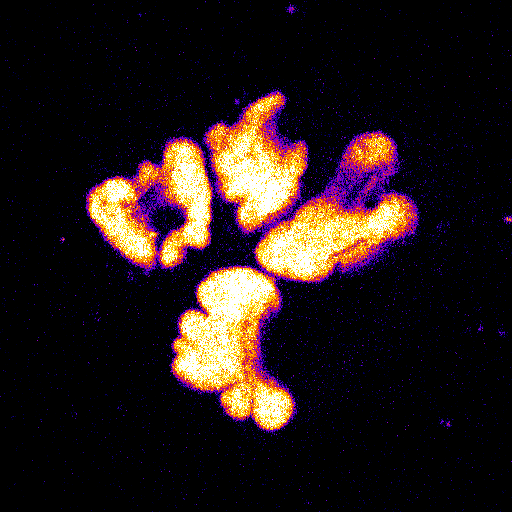

Supplement: Supplementary file 5 — Dataset S4 [file 41467_2024_55089_MOESM5_ESM.zip › Dataset_S4_representative_images/FigureS10_Representative Images/CWC27/3.tif]

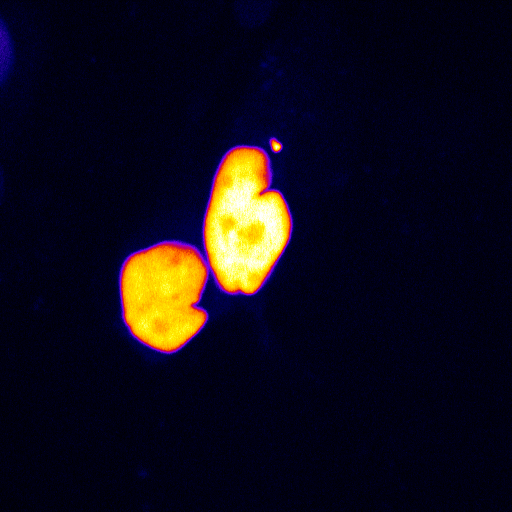

Supplement: Supplementary file 5 — Dataset S4 [file 41467_2024_55089_MOESM5_ESM.zip › Dataset_S4_representative_images/FigureS10_Representative Images/CWC27/2.tif]

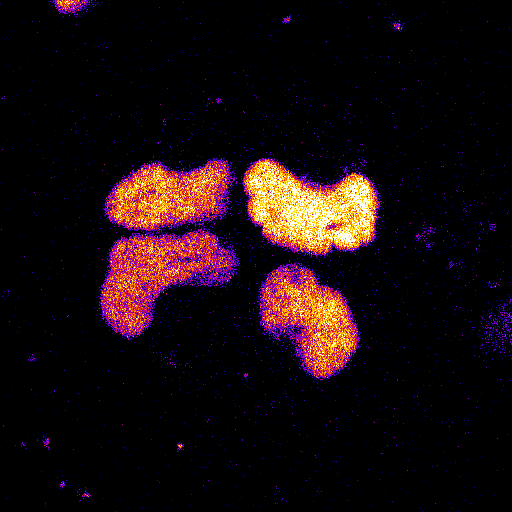

Supplement: Supplementary file 5 — Dataset S4 [file 41467_2024_55089_MOESM5_ESM.zip › Dataset_S4_representative_images/FigureS10_Representative Images/CWC27/1.tif]

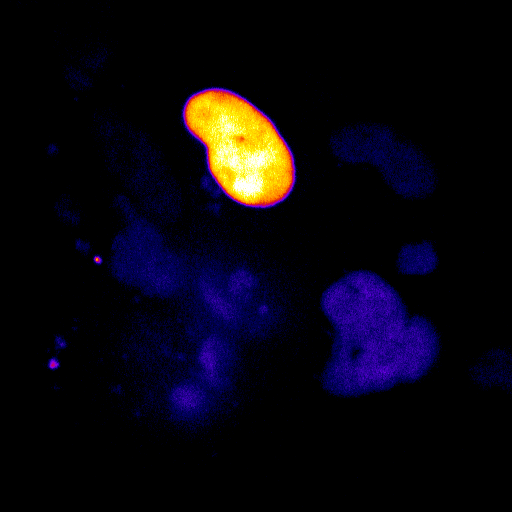

Supplement: Supplementary file 5 — Dataset S4 [file 41467_2024_55089_MOESM5_ESM.zip › Dataset_S4_representative_images/FigureS10_Representative Images/CWC27/5.tif]

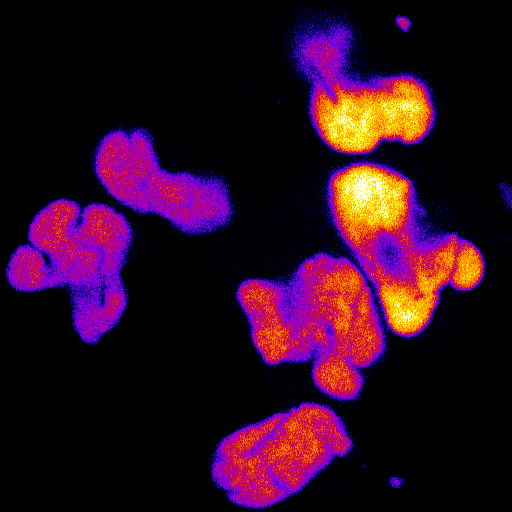

Supplement: Supplementary file 5 — Dataset S4 [file 41467_2024_55089_MOESM5_ESM.zip › Dataset_S4_representative_images/FigureS10_Representative Images/CWC27/4.tif]

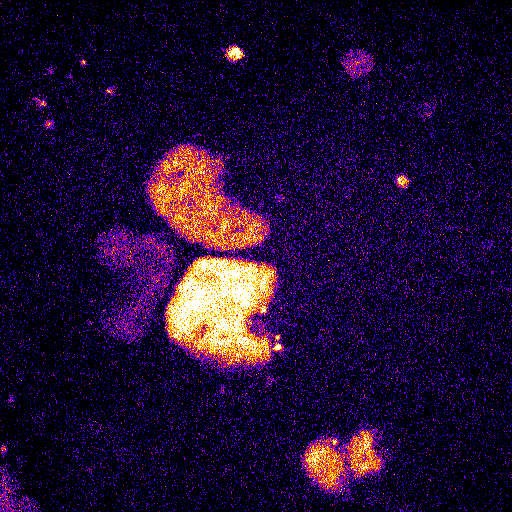

Supplement: Supplementary file 5 — Dataset S4 [file 41467_2024_55089_MOESM5_ESM.zip › Dataset_S4_representative_images/FigureS10_Representative Images/CWC27/6.tif]

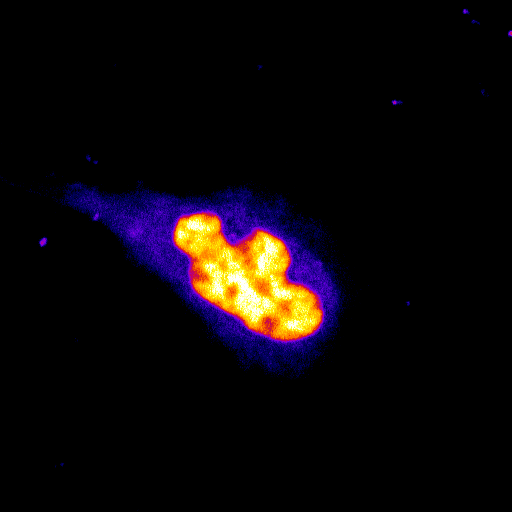

Supplement: Supplementary file 5 — Dataset S4 [file 41467_2024_55089_MOESM5_ESM.zip › Dataset_S4_representative_images/FigureS10_Representative Images/CWC27/7.tif]

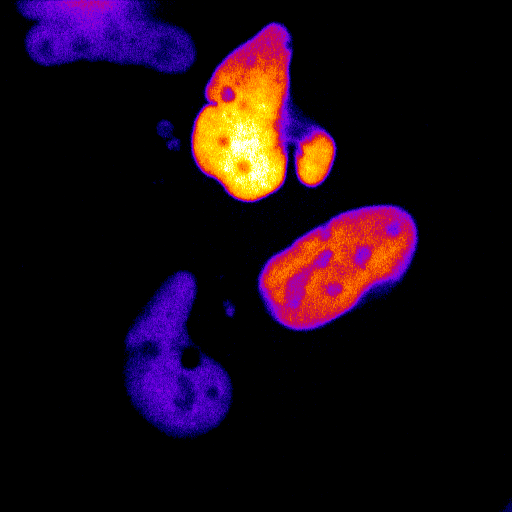

Supplement: Supplementary file 5 — Dataset S4 [file 41467_2024_55089_MOESM5_ESM.zip › Dataset_S4_representative_images/FigureS10_Representative Images/SPAG7/12.tif]

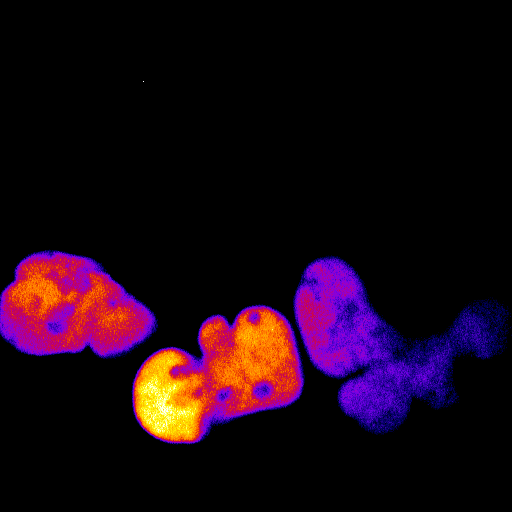

Supplement: Supplementary file 5 — Dataset S4 [file 41467_2024_55089_MOESM5_ESM.zip › Dataset_S4_representative_images/FigureS10_Representative Images/SPAG7/10.tif]

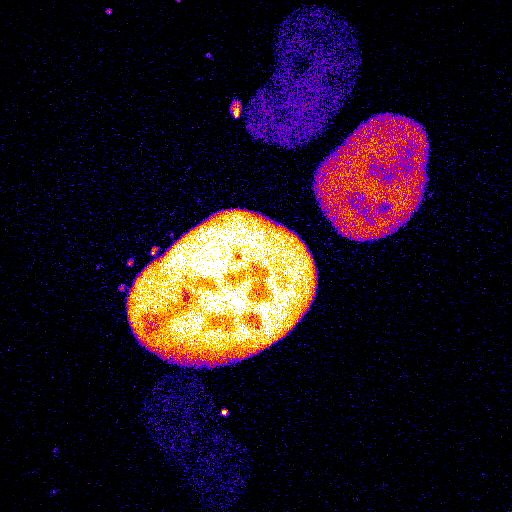

Supplement: Supplementary file 5 — Dataset S4 [file 41467_2024_55089_MOESM5_ESM.zip › Dataset_S4_representative_images/FigureS10_Representative Images/SPAG7/11.tif]

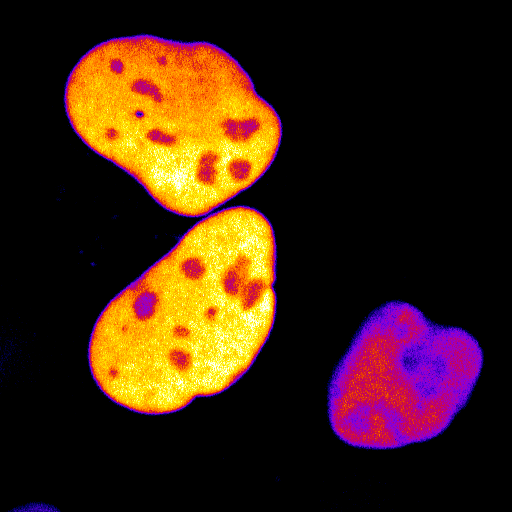

Supplement: Supplementary file 5 — Dataset S4 [file 41467_2024_55089_MOESM5_ESM.zip › Dataset_S4_representative_images/FigureS10_Representative Images/SPAG7/9.tif]

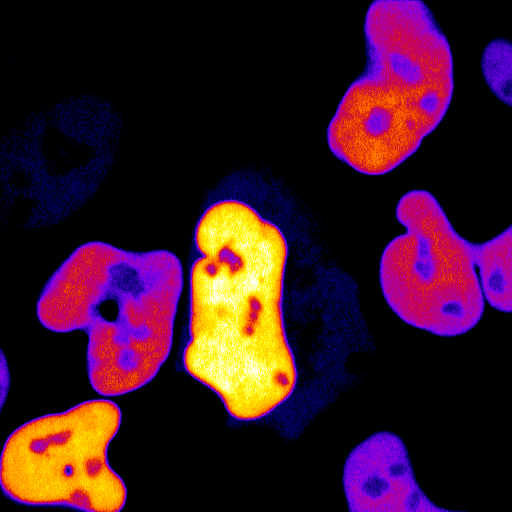

Supplement: Supplementary file 5 — Dataset S4 [file 41467_2024_55089_MOESM5_ESM.zip › Dataset_S4_representative_images/FigureS10_Representative Images/SPAG7/8.tif]

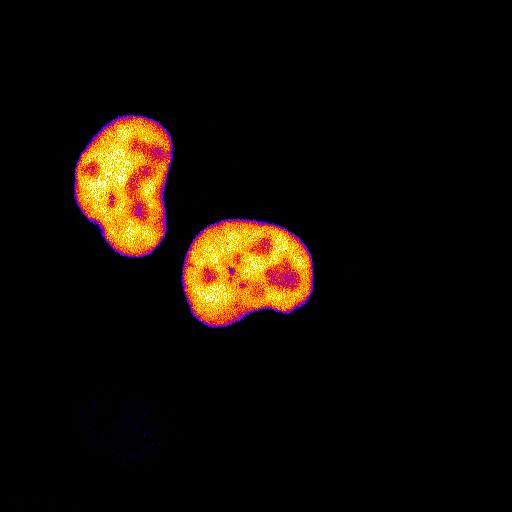

Supplement: Supplementary file 5 — Dataset S4 [file 41467_2024_55089_MOESM5_ESM.zip › Dataset_S4_representative_images/FigureS10_Representative Images/SPAG7/3.tif]

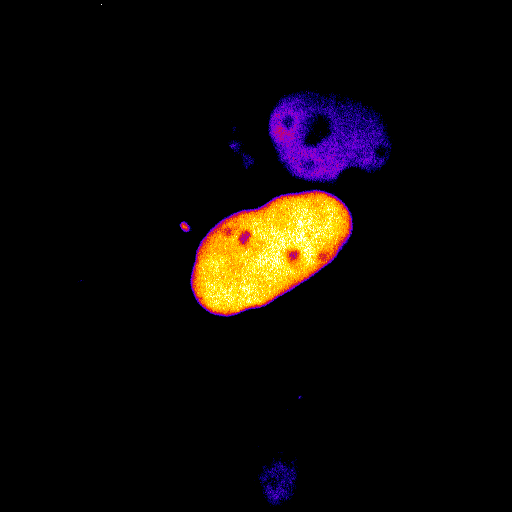

Supplement: Supplementary file 5 — Dataset S4 [file 41467_2024_55089_MOESM5_ESM.zip › Dataset_S4_representative_images/FigureS10_Representative Images/SPAG7/2.tif]

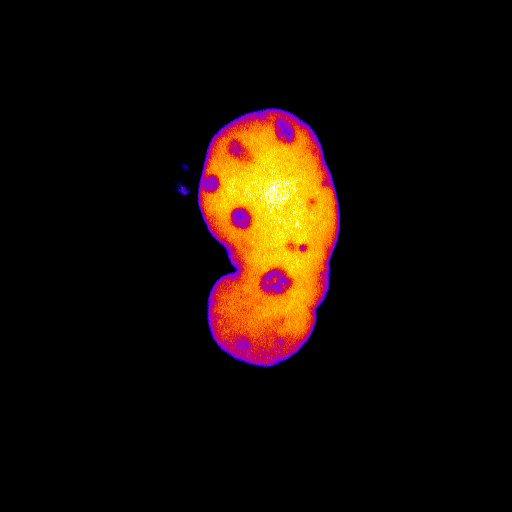

Supplement: Supplementary file 5 — Dataset S4 [file 41467_2024_55089_MOESM5_ESM.zip › Dataset_S4_representative_images/FigureS10_Representative Images/SPAG7/1.tif]

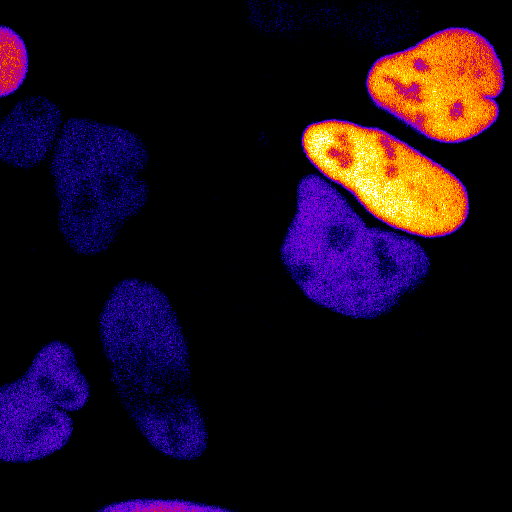

Supplement: Supplementary file 5 — Dataset S4 [file 41467_2024_55089_MOESM5_ESM.zip › Dataset_S4_representative_images/FigureS10_Representative Images/SPAG7/5.tif]

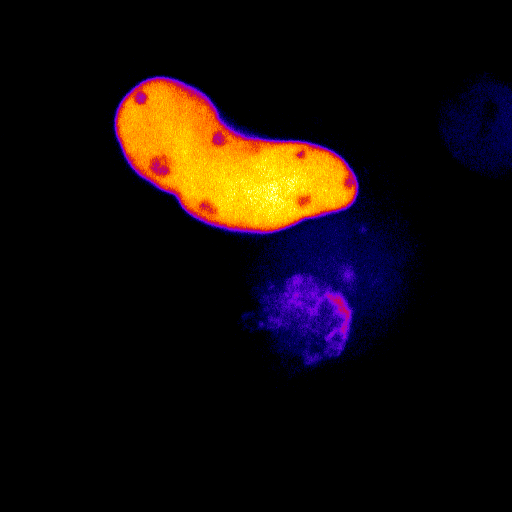

Supplement: Supplementary file 5 — Dataset S4 [file 41467_2024_55089_MOESM5_ESM.zip › Dataset_S4_representative_images/FigureS10_Representative Images/SPAG7/4.tif]

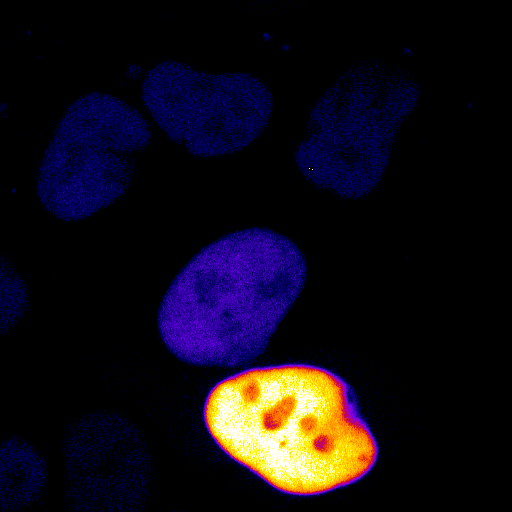

Supplement: Supplementary file 5 — Dataset S4 [file 41467_2024_55089_MOESM5_ESM.zip › Dataset_S4_representative_images/FigureS10_Representative Images/SPAG7/6.tif]

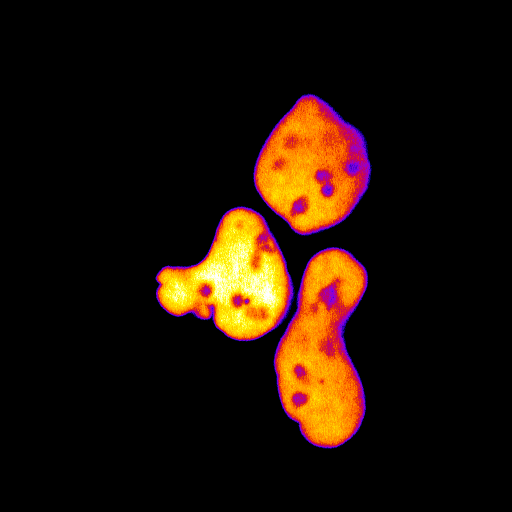

Supplement: Supplementary file 5 — Dataset S4 [file 41467_2024_55089_MOESM5_ESM.zip › Dataset_S4_representative_images/FigureS10_Representative Images/SPAG7/7.tif]

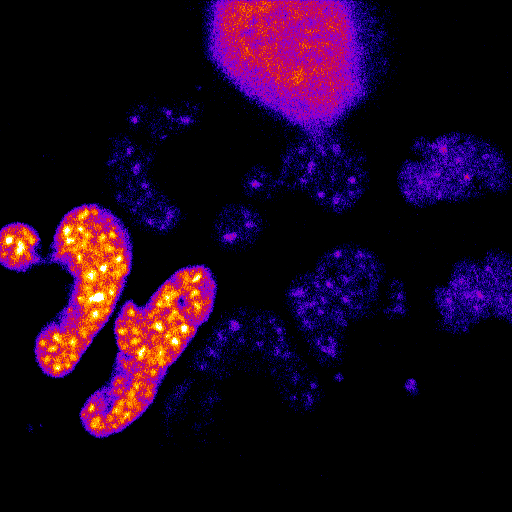

Supplement: Supplementary file 5 — Dataset S4 [file 41467_2024_55089_MOESM5_ESM.zip › Dataset_S4_representative_images/FigureS10_Representative Images/RP9/13.tif]

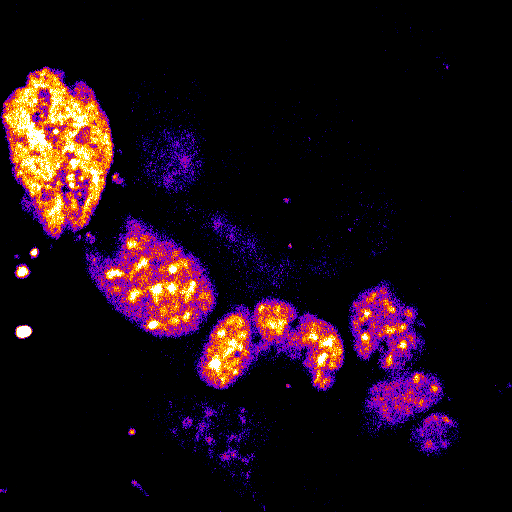

Supplement: Supplementary file 5 — Dataset S4 [file 41467_2024_55089_MOESM5_ESM.zip › Dataset_S4_representative_images/FigureS10_Representative Images/RP9/12.tif]

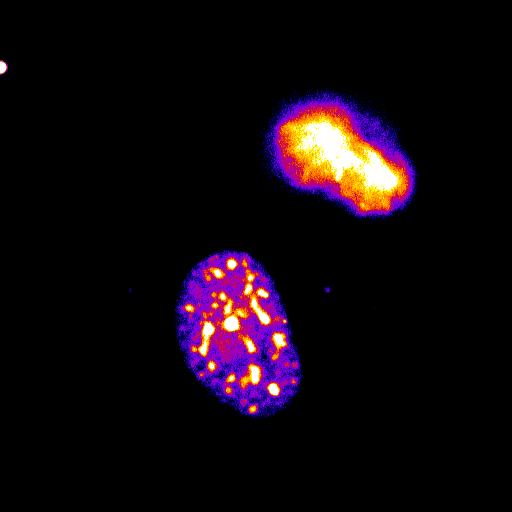

Supplement: Supplementary file 5 — Dataset S4 [file 41467_2024_55089_MOESM5_ESM.zip › Dataset_S4_representative_images/FigureS10_Representative Images/RP9/10.tif]

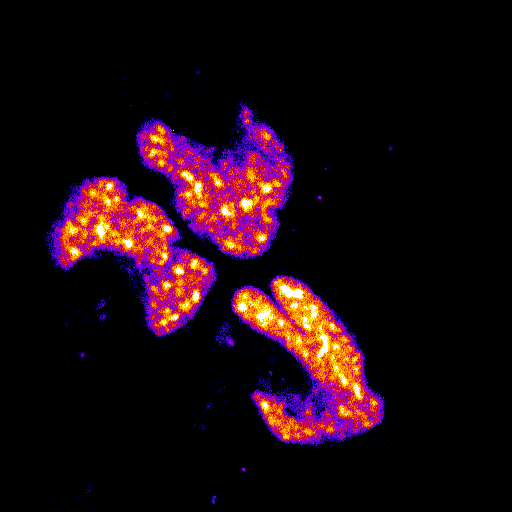

Supplement: Supplementary file 5 — Dataset S4 [file 41467_2024_55089_MOESM5_ESM.zip › Dataset_S4_representative_images/FigureS10_Representative Images/RP9/11.tif]

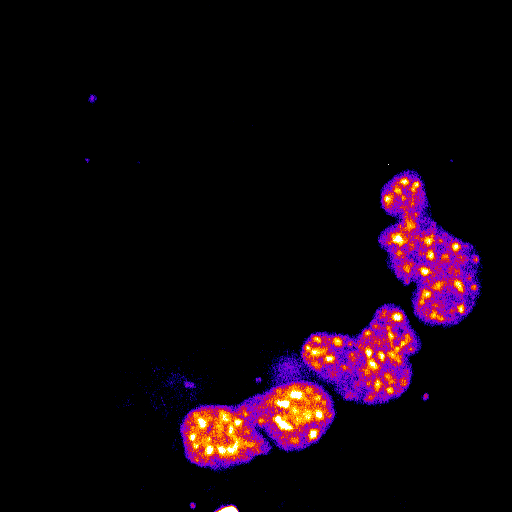

Supplement: Supplementary file 5 — Dataset S4 [file 41467_2024_55089_MOESM5_ESM.zip › Dataset_S4_representative_images/FigureS10_Representative Images/RP9/9.tif]

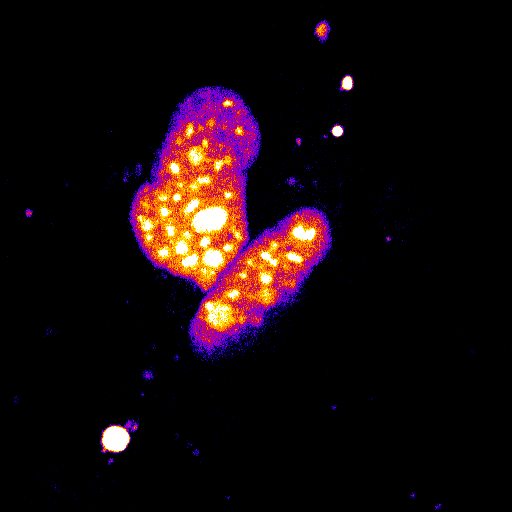

Supplement: Supplementary file 5 — Dataset S4 [file 41467_2024_55089_MOESM5_ESM.zip › Dataset_S4_representative_images/FigureS10_Representative Images/RP9/8.tif]

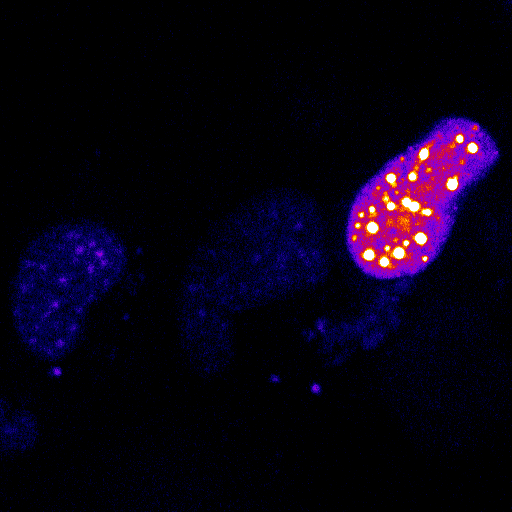

Supplement: Supplementary file 5 — Dataset S4 [file 41467_2024_55089_MOESM5_ESM.zip › Dataset_S4_representative_images/FigureS10_Representative Images/RP9/3.tif]

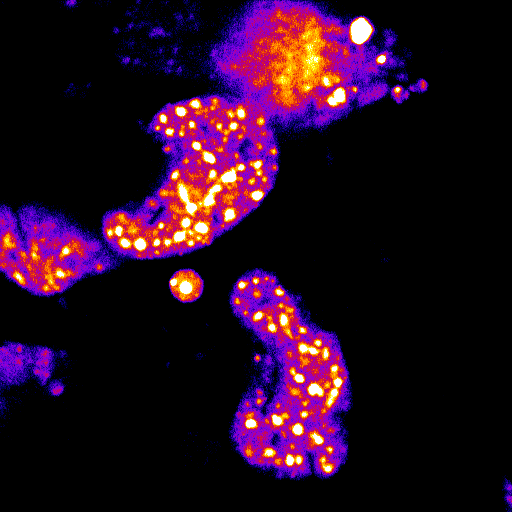

Supplement: Supplementary file 5 — Dataset S4 [file 41467_2024_55089_MOESM5_ESM.zip › Dataset_S4_representative_images/FigureS10_Representative Images/RP9/2.tif]

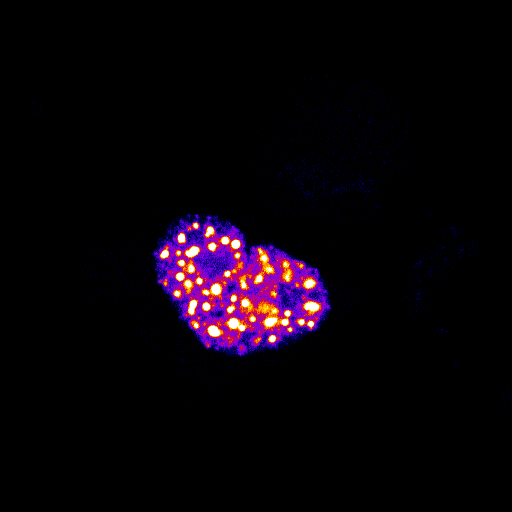

Supplement: Supplementary file 5 — Dataset S4 [file 41467_2024_55089_MOESM5_ESM.zip › Dataset_S4_representative_images/FigureS10_Representative Images/RP9/1.tif]

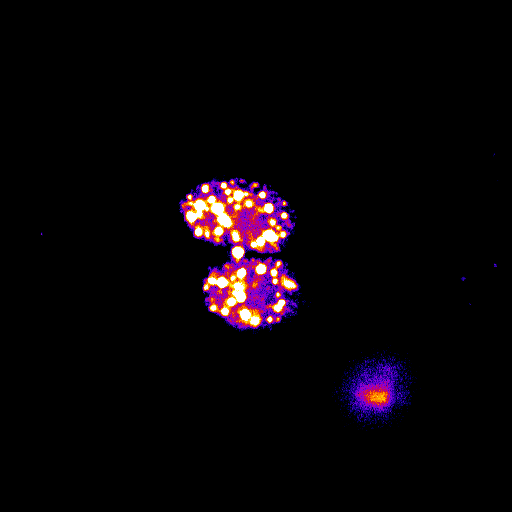

Supplement: Supplementary file 5 — Dataset S4 [file 41467_2024_55089_MOESM5_ESM.zip › Dataset_S4_representative_images/FigureS10_Representative Images/RP9/5.tif]

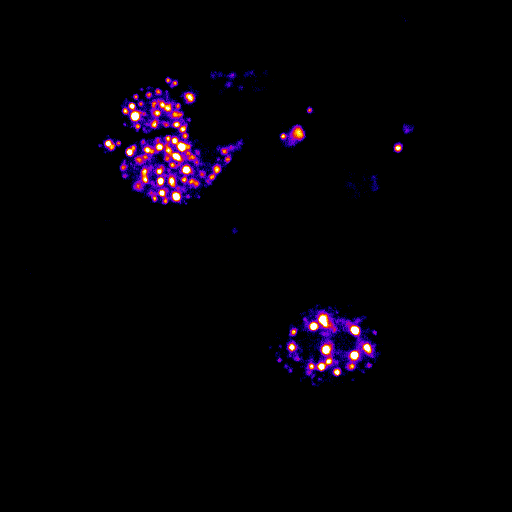

Supplement: Supplementary file 5 — Dataset S4 [file 41467_2024_55089_MOESM5_ESM.zip › Dataset_S4_representative_images/FigureS10_Representative Images/RP9/4.tif]

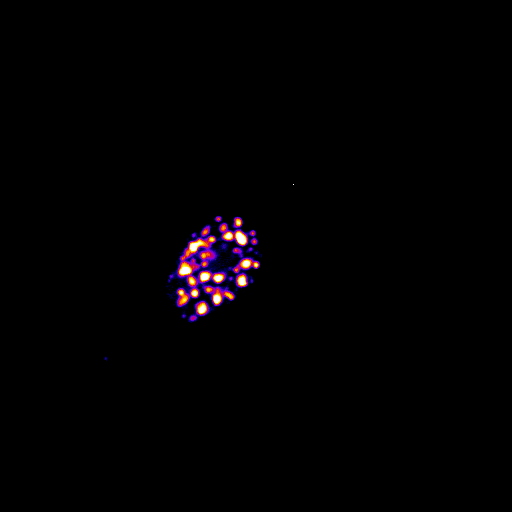

Supplement: Supplementary file 5 — Dataset S4 [file 41467_2024_55089_MOESM5_ESM.zip › Dataset_S4_representative_images/FigureS10_Representative Images/RP9/6.tif]

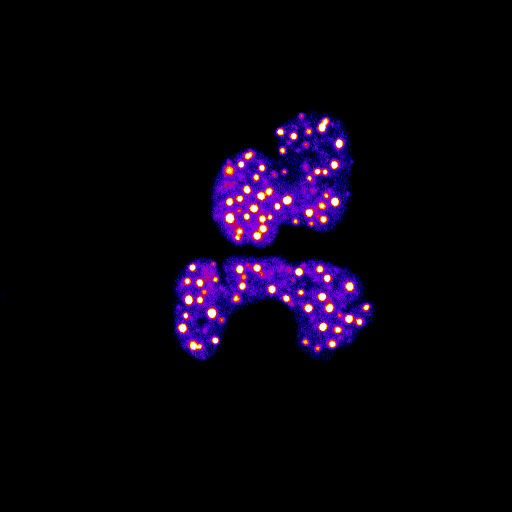

Supplement: Supplementary file 5 — Dataset S4 [file 41467_2024_55089_MOESM5_ESM.zip › Dataset_S4_representative_images/FigureS10_Representative Images/RP9/7.tif]

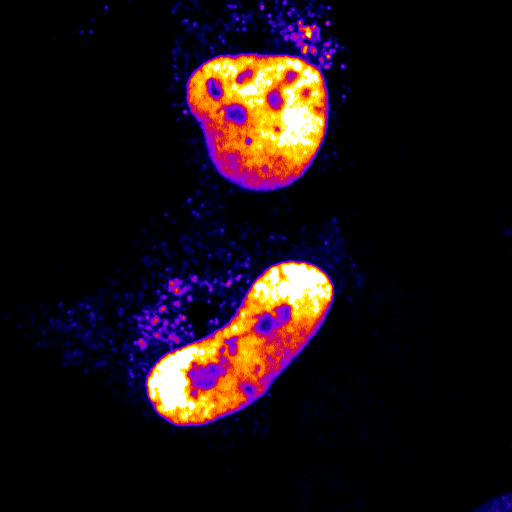

Supplement: Supplementary file 5 — Dataset S4 [file 41467_2024_55089_MOESM5_ESM.zip › Dataset_S4_representative_images/FigureS10_Representative Images/RBMY1D/12.tif]

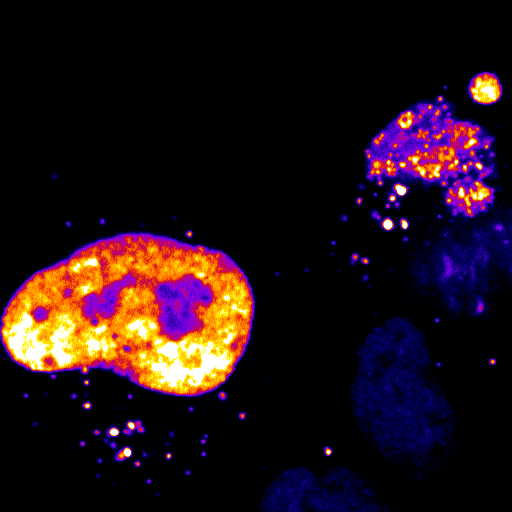

Supplement: Supplementary file 5 — Dataset S4 [file 41467_2024_55089_MOESM5_ESM.zip › Dataset_S4_representative_images/FigureS10_Representative Images/RBMY1D/10.tif]

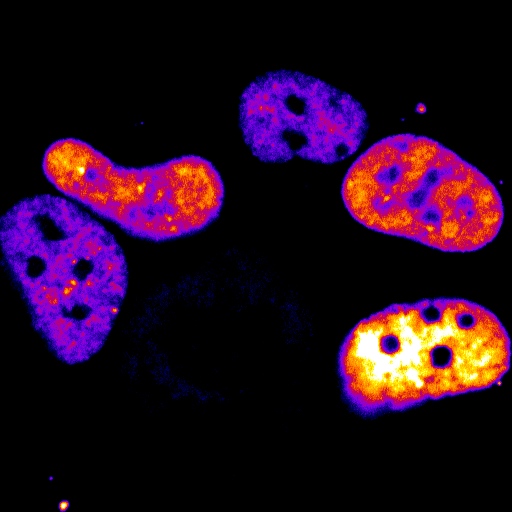

Supplement: Supplementary file 5 — Dataset S4 [file 41467_2024_55089_MOESM5_ESM.zip › Dataset_S4_representative_images/FigureS10_Representative Images/RBMY1D/11.tif]

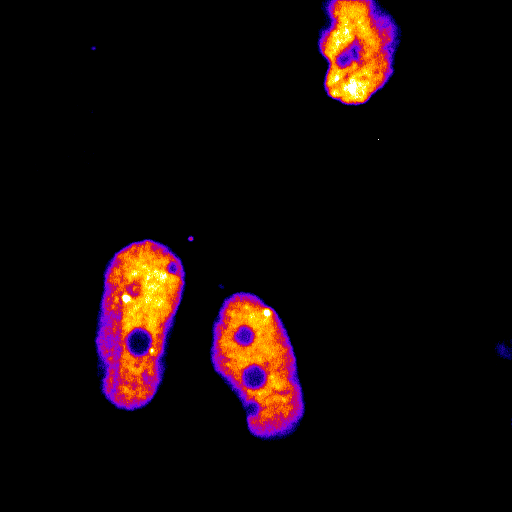

Supplement: Supplementary file 5 — Dataset S4 [file 41467_2024_55089_MOESM5_ESM.zip › Dataset_S4_representative_images/FigureS10_Representative Images/RBMY1D/9.tif]

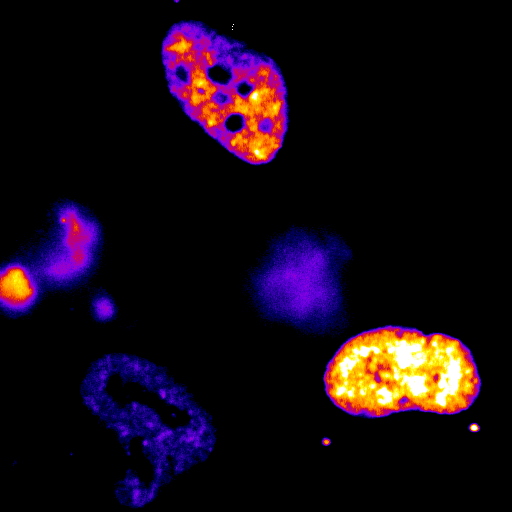

Supplement: Supplementary file 5 — Dataset S4 [file 41467_2024_55089_MOESM5_ESM.zip › Dataset_S4_representative_images/FigureS10_Representative Images/RBMY1D/8.tif]

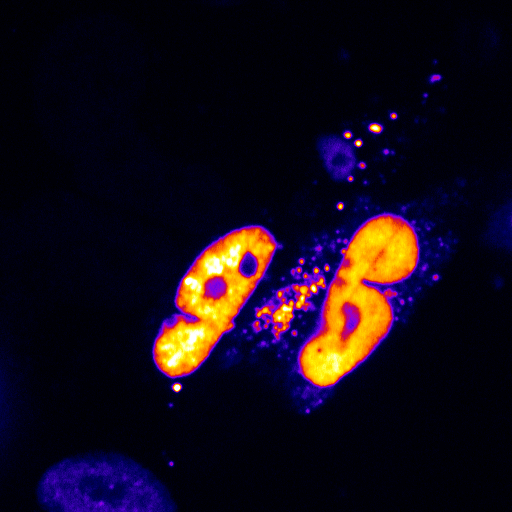

Supplement: Supplementary file 5 — Dataset S4 [file 41467_2024_55089_MOESM5_ESM.zip › Dataset_S4_representative_images/FigureS10_Representative Images/RBMY1D/3.tif]

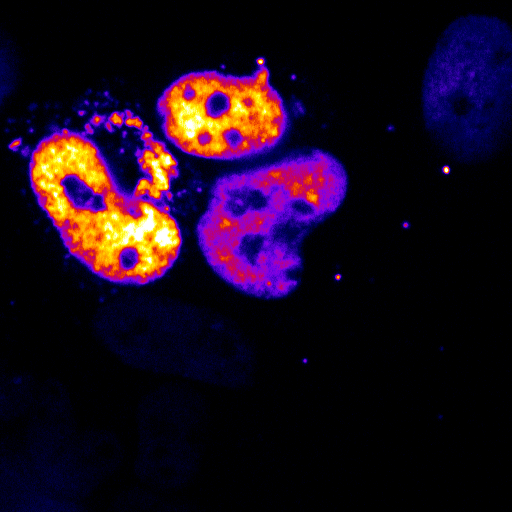

Supplement: Supplementary file 5 — Dataset S4 [file 41467_2024_55089_MOESM5_ESM.zip › Dataset_S4_representative_images/FigureS10_Representative Images/RBMY1D/2.tif]

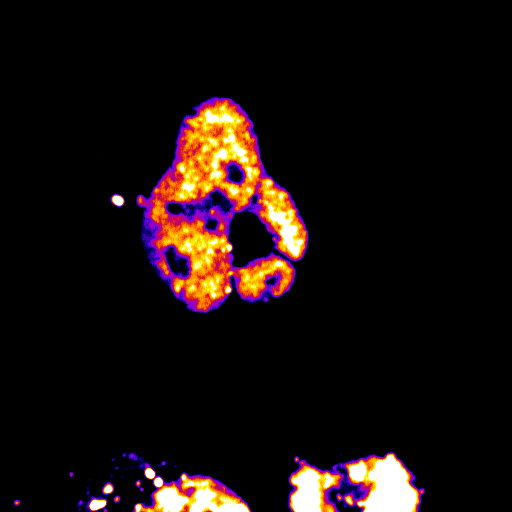

Supplement: Supplementary file 5 — Dataset S4 [file 41467_2024_55089_MOESM5_ESM.zip › Dataset_S4_representative_images/FigureS10_Representative Images/RBMY1D/1.tif]

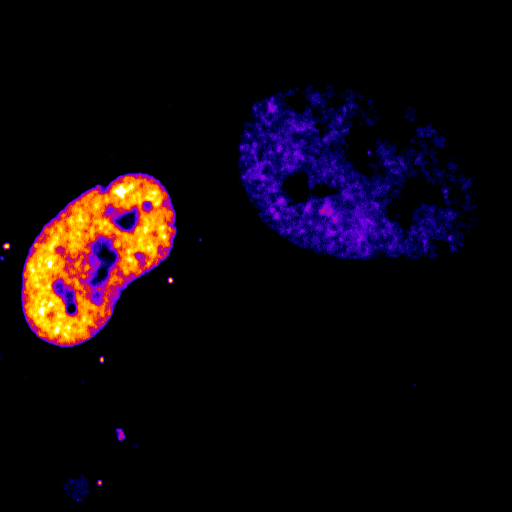

Supplement: Supplementary file 5 — Dataset S4 [file 41467_2024_55089_MOESM5_ESM.zip › Dataset_S4_representative_images/FigureS10_Representative Images/RBMY1D/5.tif]

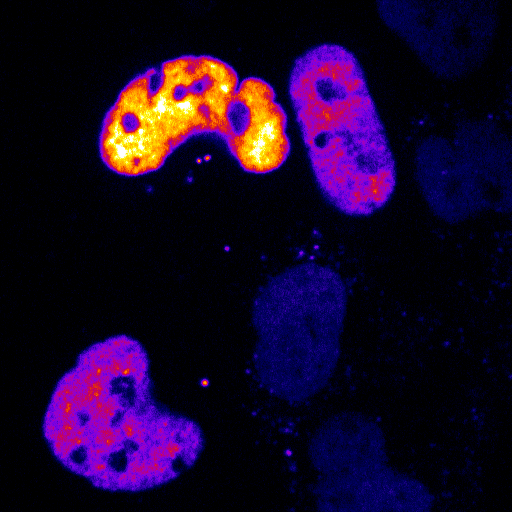

Supplement: Supplementary file 5 — Dataset S4 [file 41467_2024_55089_MOESM5_ESM.zip › Dataset_S4_representative_images/FigureS10_Representative Images/RBMY1D/4.tif]

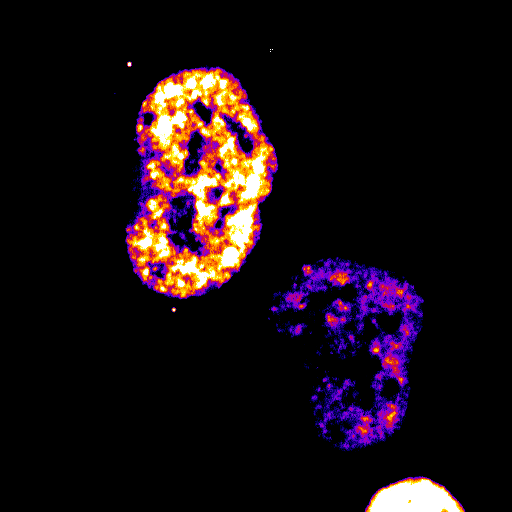

Supplement: Supplementary file 5 — Dataset S4 [file 41467_2024_55089_MOESM5_ESM.zip › Dataset_S4_representative_images/FigureS10_Representative Images/RBMY1D/6.tif]

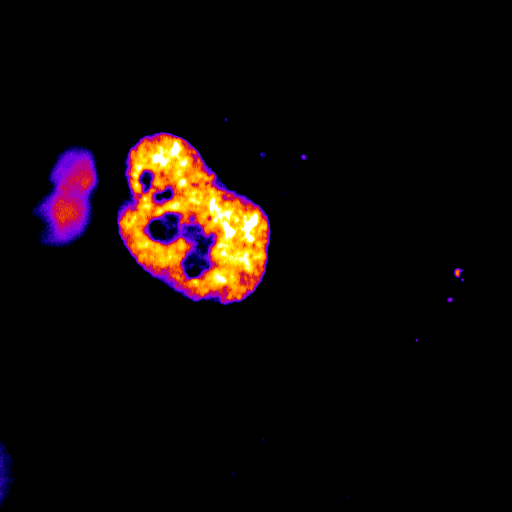

Supplement: Supplementary file 5 — Dataset S4 [file 41467_2024_55089_MOESM5_ESM.zip › Dataset_S4_representative_images/FigureS10_Representative Images/RBMY1D/7.tif]

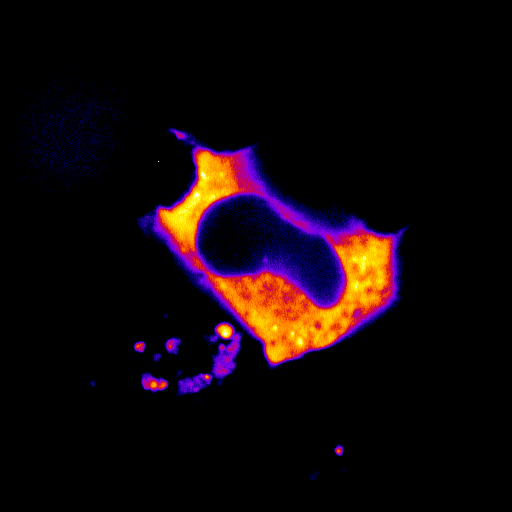

Supplement: Supplementary file 5 — Dataset S4 [file 41467_2024_55089_MOESM5_ESM.zip › Dataset_S4_representative_images/FigureS10_Representative Images/HBS1L/10.tif]

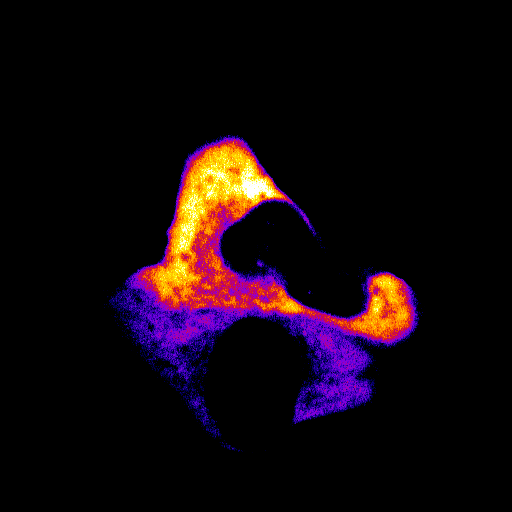

Supplement: Supplementary file 5 — Dataset S4 [file 41467_2024_55089_MOESM5_ESM.zip › Dataset_S4_representative_images/FigureS10_Representative Images/HBS1L/11.tif]

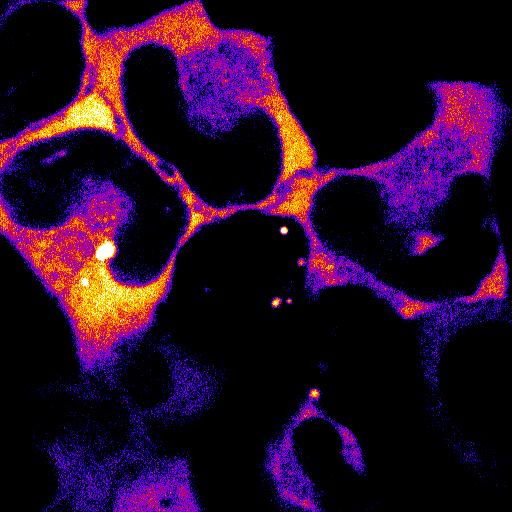

Supplement: Supplementary file 5 — Dataset S4 [file 41467_2024_55089_MOESM5_ESM.zip › Dataset_S4_representative_images/FigureS10_Representative Images/HBS1L/9.tif]

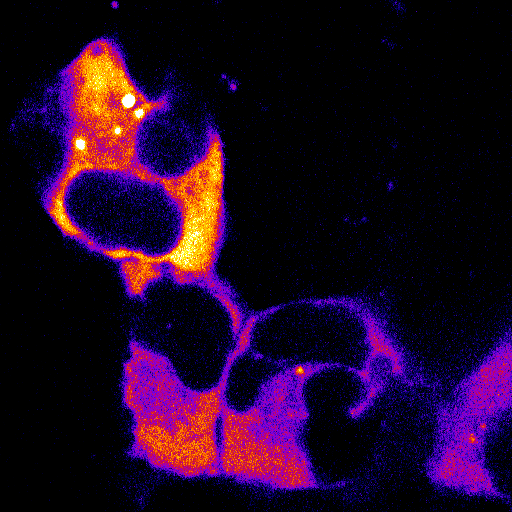

Supplement: Supplementary file 5 — Dataset S4 [file 41467_2024_55089_MOESM5_ESM.zip › Dataset_S4_representative_images/FigureS10_Representative Images/HBS1L/8.tif]

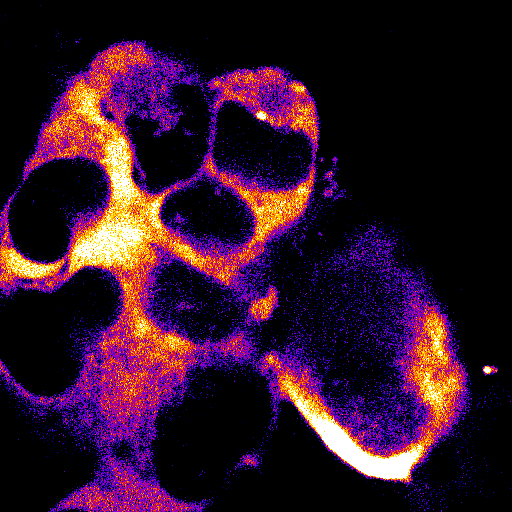

Supplement: Supplementary file 5 — Dataset S4 [file 41467_2024_55089_MOESM5_ESM.zip › Dataset_S4_representative_images/FigureS10_Representative Images/HBS1L/3.tif]

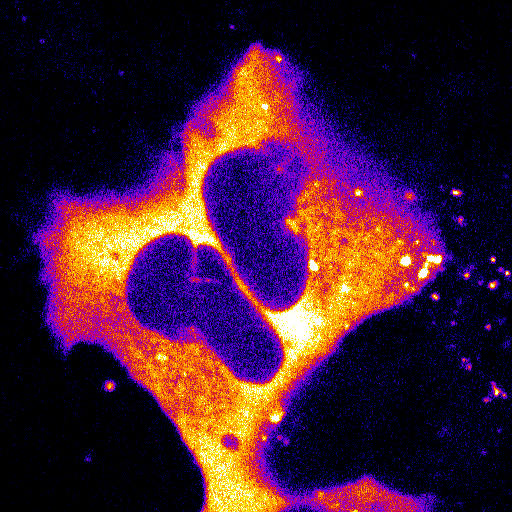

Supplement: Supplementary file 5 — Dataset S4 [file 41467_2024_55089_MOESM5_ESM.zip › Dataset_S4_representative_images/FigureS10_Representative Images/HBS1L/2.tif]

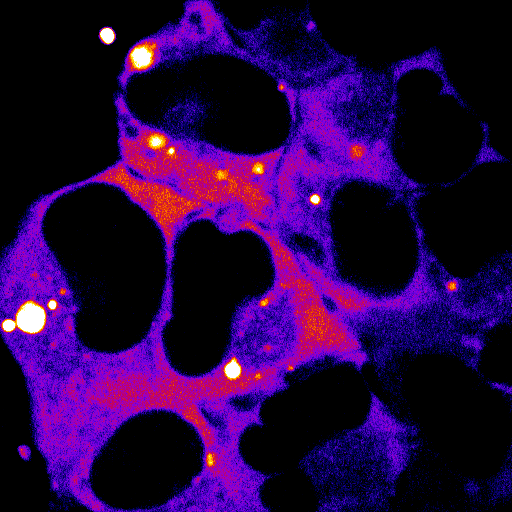

Supplement: Supplementary file 5 — Dataset S4 [file 41467_2024_55089_MOESM5_ESM.zip › Dataset_S4_representative_images/FigureS10_Representative Images/HBS1L/1.tif]

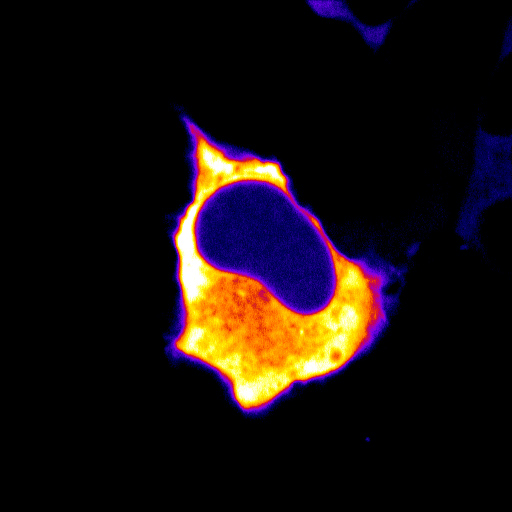

Supplement: Supplementary file 5 — Dataset S4 [file 41467_2024_55089_MOESM5_ESM.zip › Dataset_S4_representative_images/FigureS10_Representative Images/HBS1L/5.tif]

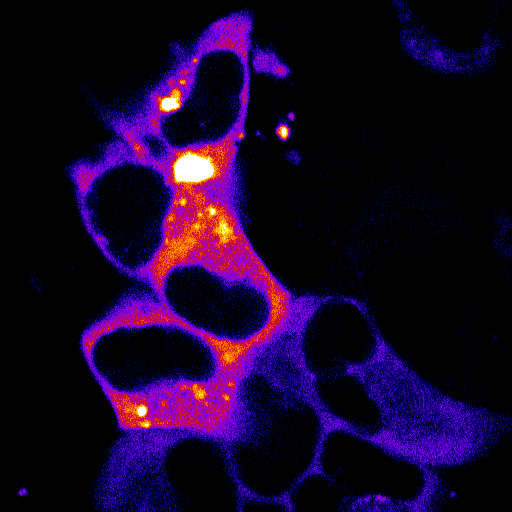

Supplement: Supplementary file 5 — Dataset S4 [file 41467_2024_55089_MOESM5_ESM.zip › Dataset_S4_representative_images/FigureS10_Representative Images/HBS1L/4.tif]

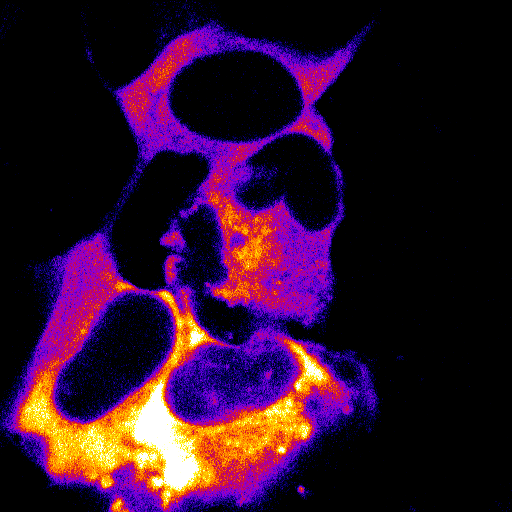

Supplement: Supplementary file 5 — Dataset S4 [file 41467_2024_55089_MOESM5_ESM.zip › Dataset_S4_representative_images/FigureS10_Representative Images/HBS1L/6.tif]

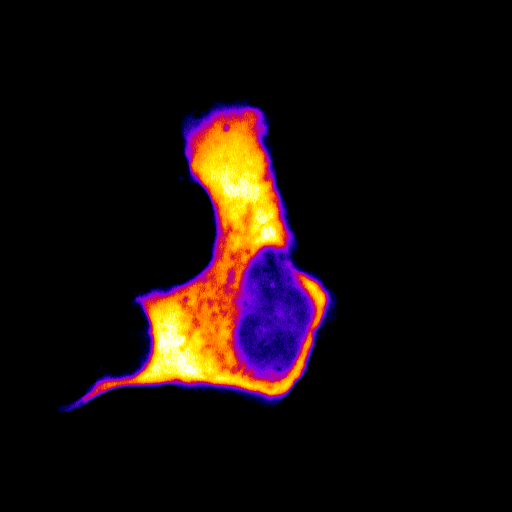

Supplement: Supplementary file 5 — Dataset S4 [file 41467_2024_55089_MOESM5_ESM.zip › Dataset_S4_representative_images/FigureS10_Representative Images/HBS1L/7.tif]

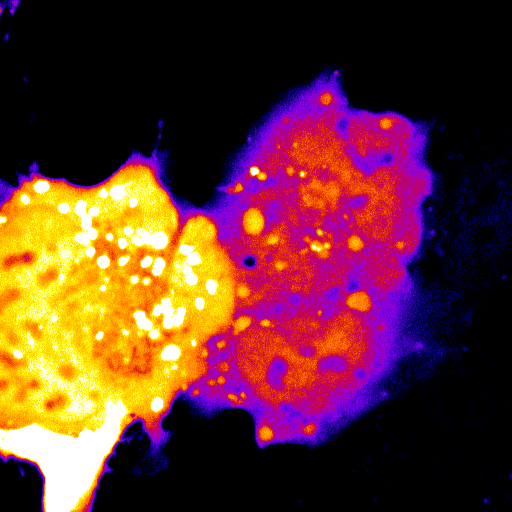

Supplement: Supplementary file 5 — Dataset S4 [file 41467_2024_55089_MOESM5_ESM.zip › Dataset_S4_representative_images/FigureS10_Representative Images/PHP14/10.tif]

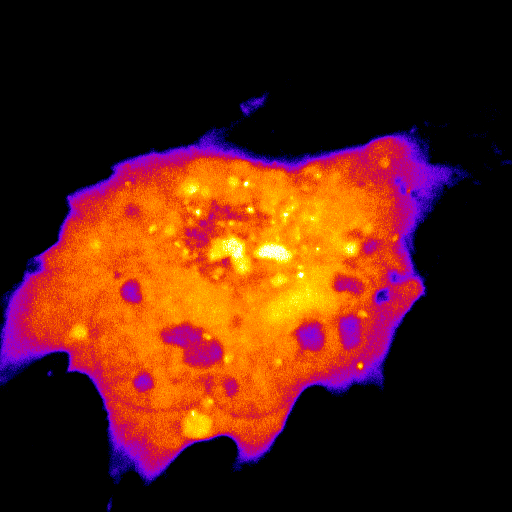

Supplement: Supplementary file 5 — Dataset S4 [file 41467_2024_55089_MOESM5_ESM.zip › Dataset_S4_representative_images/FigureS10_Representative Images/PHP14/11.tif]

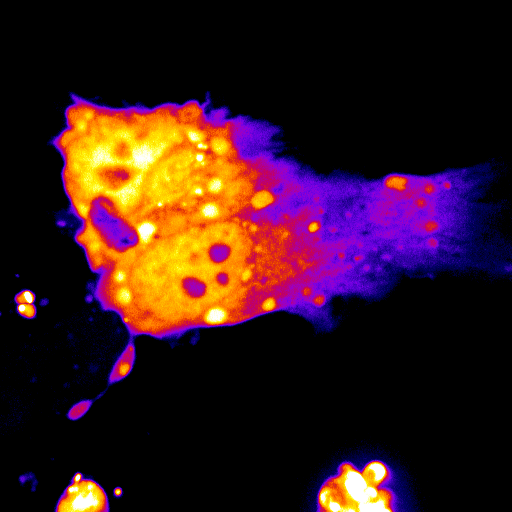

Supplement: Supplementary file 5 — Dataset S4 [file 41467_2024_55089_MOESM5_ESM.zip › Dataset_S4_representative_images/FigureS10_Representative Images/PHP14/9.tif]

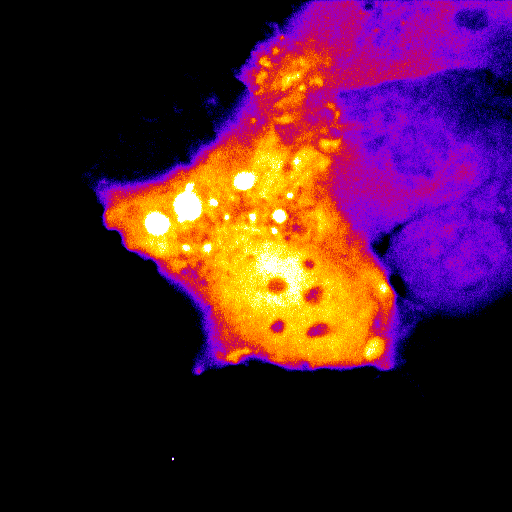

Supplement: Supplementary file 5 — Dataset S4 [file 41467_2024_55089_MOESM5_ESM.zip › Dataset_S4_representative_images/FigureS10_Representative Images/PHP14/8.tif]

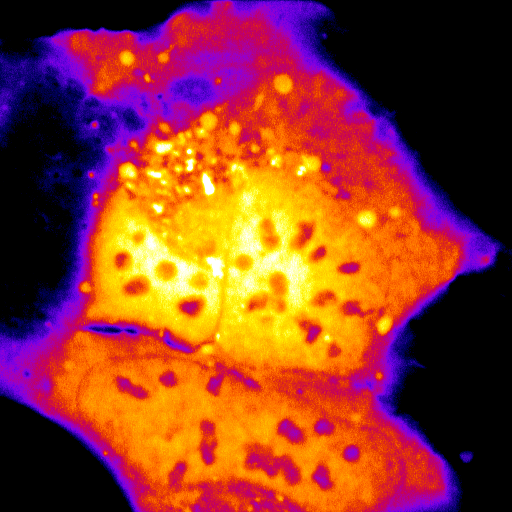

Supplement: Supplementary file 5 — Dataset S4 [file 41467_2024_55089_MOESM5_ESM.zip › Dataset_S4_representative_images/FigureS10_Representative Images/PHP14/3.tif]

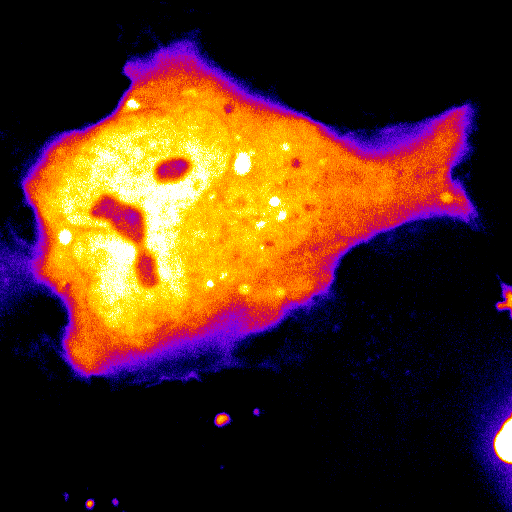

Supplement: Supplementary file 5 — Dataset S4 [file 41467_2024_55089_MOESM5_ESM.zip › Dataset_S4_representative_images/FigureS10_Representative Images/PHP14/2.tif]

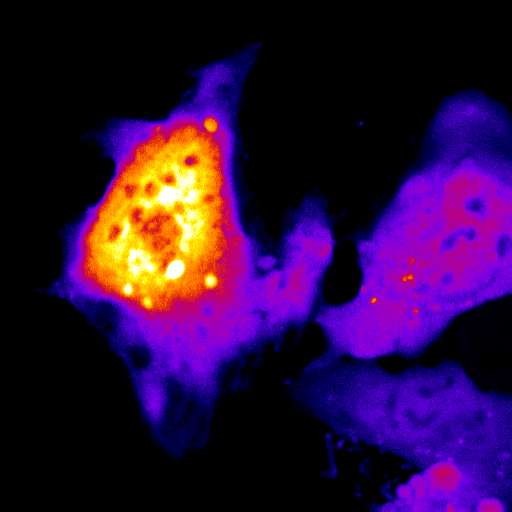

Supplement: Supplementary file 5 — Dataset S4 [file 41467_2024_55089_MOESM5_ESM.zip › Dataset_S4_representative_images/FigureS10_Representative Images/PHP14/1.tif]

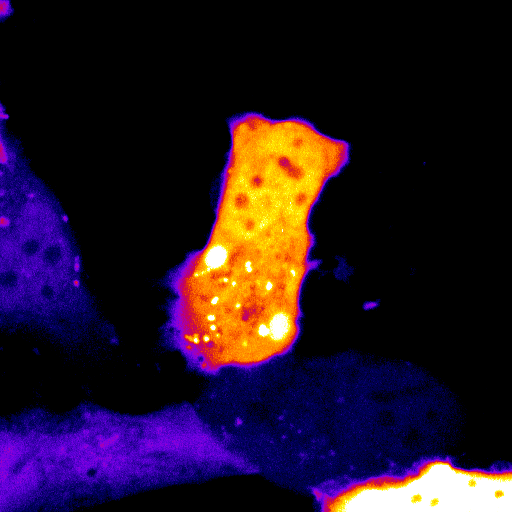

Supplement: Supplementary file 5 — Dataset S4 [file 41467_2024_55089_MOESM5_ESM.zip › Dataset_S4_representative_images/FigureS10_Representative Images/PHP14/5.tif]

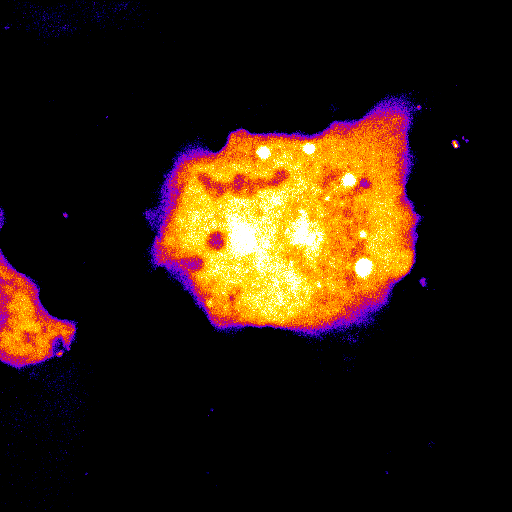

Supplement: Supplementary file 5 — Dataset S4 [file 41467_2024_55089_MOESM5_ESM.zip › Dataset_S4_representative_images/FigureS10_Representative Images/PHP14/4.tif]

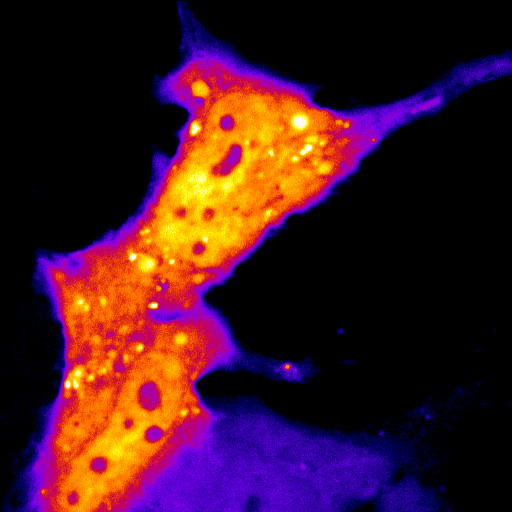

Supplement: Supplementary file 5 — Dataset S4 [file 41467_2024_55089_MOESM5_ESM.zip › Dataset_S4_representative_images/FigureS10_Representative Images/PHP14/6.tif]

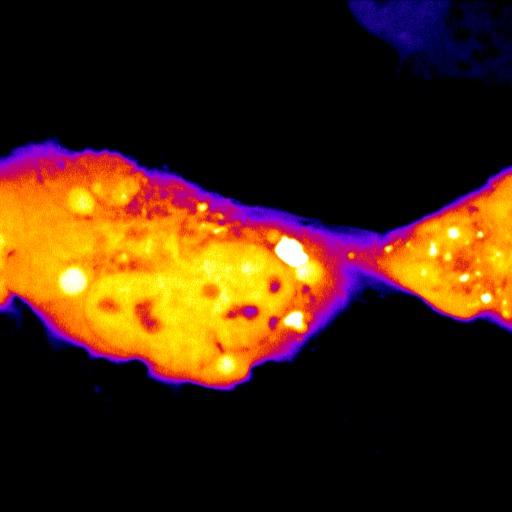

Supplement: Supplementary file 5 — Dataset S4 [file 41467_2024_55089_MOESM5_ESM.zip › Dataset_S4_representative_images/FigureS10_Representative Images/PHP14/7.tif]

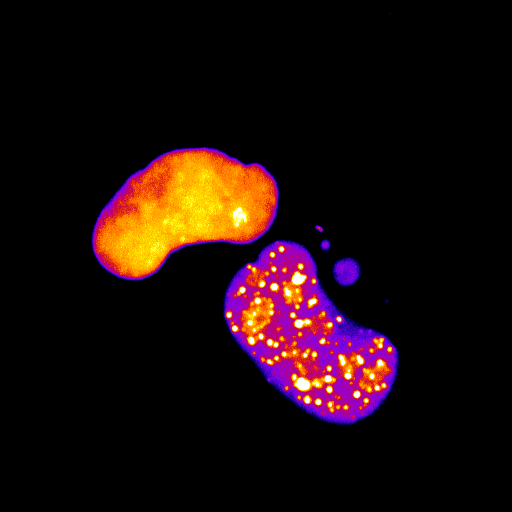

Supplement: Supplementary file 5 — Dataset S4 [file 41467_2024_55089_MOESM5_ESM.zip › Dataset_S4_representative_images/FigureS10_Representative Images/RAD51-AP1/10.tif]

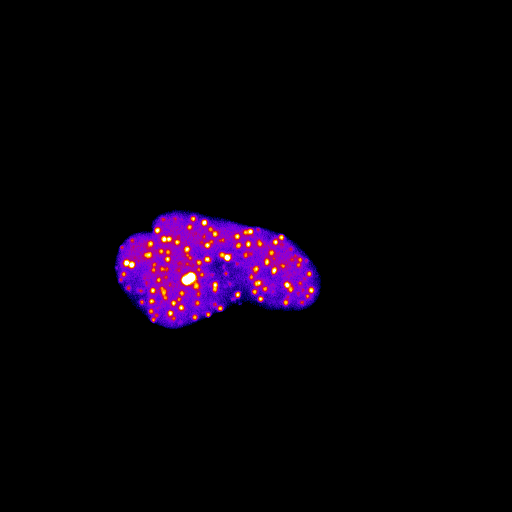

Supplement: Supplementary file 5 — Dataset S4 [file 41467_2024_55089_MOESM5_ESM.zip › Dataset_S4_representative_images/FigureS10_Representative Images/RAD51-AP1/11.tif]

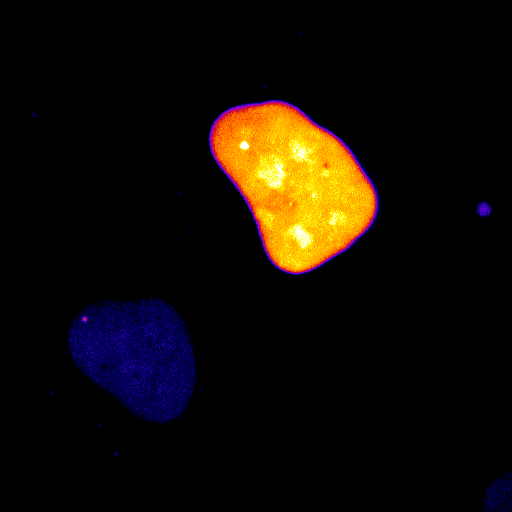

Supplement: Supplementary file 5 — Dataset S4 [file 41467_2024_55089_MOESM5_ESM.zip › Dataset_S4_representative_images/FigureS10_Representative Images/RAD51-AP1/9.tif]

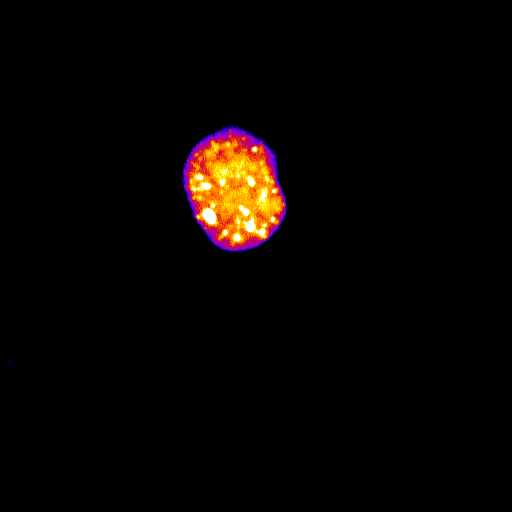

Supplement: Supplementary file 5 — Dataset S4 [file 41467_2024_55089_MOESM5_ESM.zip › Dataset_S4_representative_images/FigureS10_Representative Images/RAD51-AP1/8.tif]

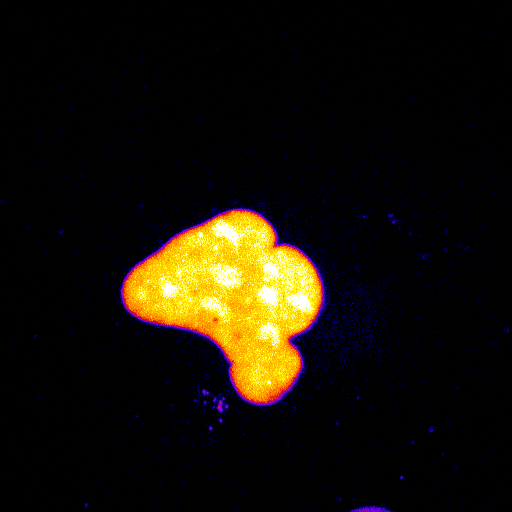

Supplement: Supplementary file 5 — Dataset S4 [file 41467_2024_55089_MOESM5_ESM.zip › Dataset_S4_representative_images/FigureS10_Representative Images/RAD51-AP1/3.tif]

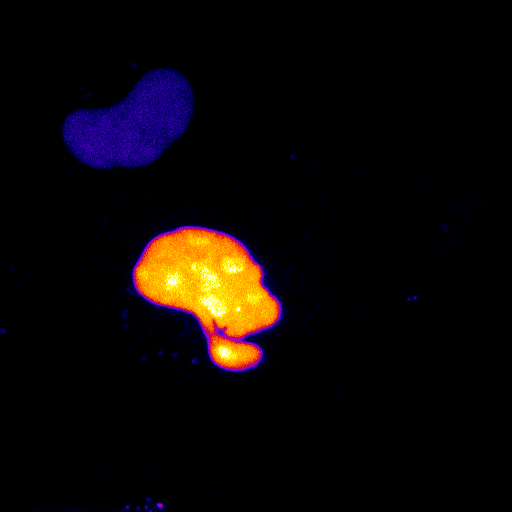

Supplement: Supplementary file 5 — Dataset S4 [file 41467_2024_55089_MOESM5_ESM.zip › Dataset_S4_representative_images/FigureS10_Representative Images/RAD51-AP1/2.tif]

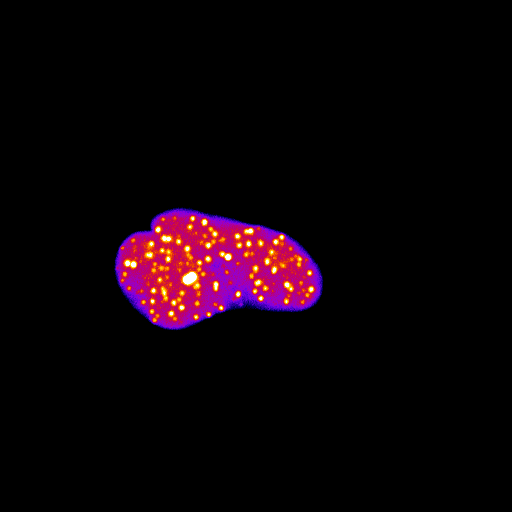

Supplement: Supplementary file 5 — Dataset S4 [file 41467_2024_55089_MOESM5_ESM.zip › Dataset_S4_representative_images/FigureS10_Representative Images/RAD51-AP1/1.tif]

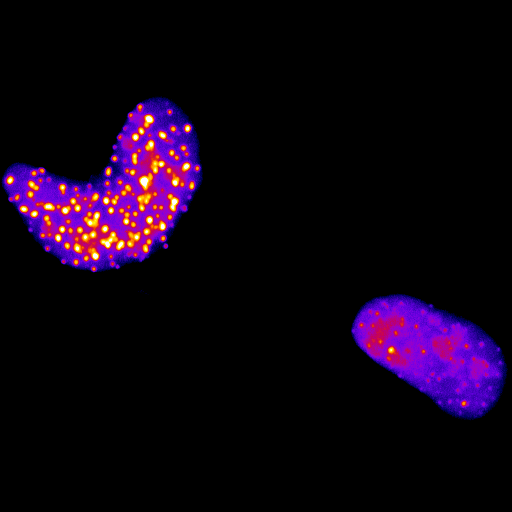

Supplement: Supplementary file 5 — Dataset S4 [file 41467_2024_55089_MOESM5_ESM.zip › Dataset_S4_representative_images/FigureS10_Representative Images/RAD51-AP1/5.tif]
