# Supplementary figures and images for: PICNIC accurately predicts condensate-forming proteins regardless of their structural disorder across organisms (part 1 of 3)
Source: Nat Commun. 2024 Dec 11;15:10668. doi: 10.1038/s41467-024-55089-x (PMC11634905; doi:10.1038/s41467-024-55089-x)

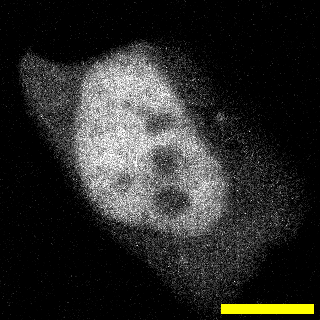

Supplement: Supplementary file 5 — Dataset S4 [file 41467_2024_55089_MOESM5_ESM.zip › Dataset_S4_representative_images/FigureS11_Negative_predictions/LRRC10.tif]

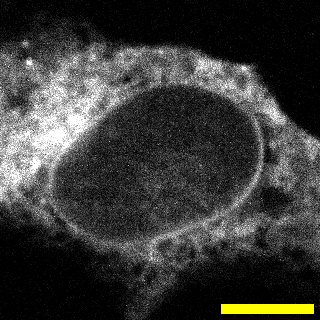

Supplement: Supplementary file 5 — Dataset S4 [file 41467_2024_55089_MOESM5_ESM.zip › Dataset_S4_representative_images/FigureS11_Negative_predictions/CRISP1.tif]

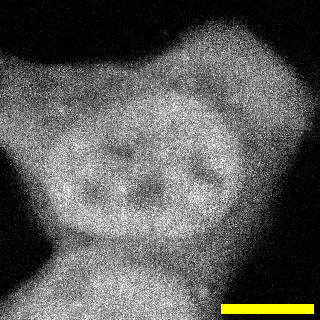

Supplement: Supplementary file 5 — Dataset S4 [file 41467_2024_55089_MOESM5_ESM.zip › Dataset_S4_representative_images/FigureS11_Negative_predictions/KAP23.1.tif]

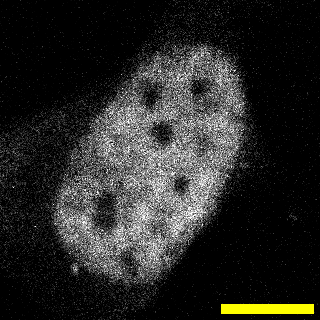

Supplement: Supplementary file 5 — Dataset S4 [file 41467_2024_55089_MOESM5_ESM.zip › Dataset_S4_representative_images/FigureS11_Negative_predictions/FANCC.tif]

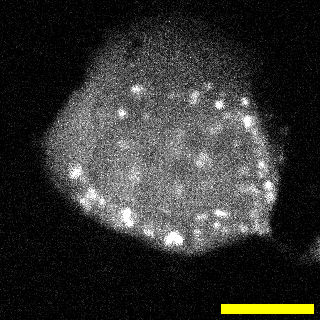

Supplement: Supplementary file 5 — Dataset S4 [file 41467_2024_55089_MOESM5_ESM.zip › Dataset_S4_representative_images/FigureS11_Negative_predictions/GTF2A2.tif]

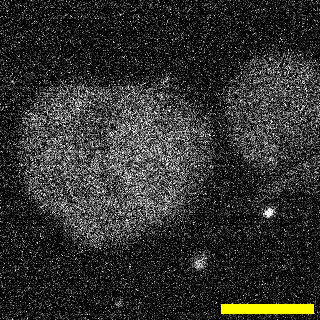

Supplement: Supplementary file 5 — Dataset S4 [file 41467_2024_55089_MOESM5_ESM.zip › Dataset_S4_representative_images/FigureS11_Negative_predictions/IGLJ3.tif]

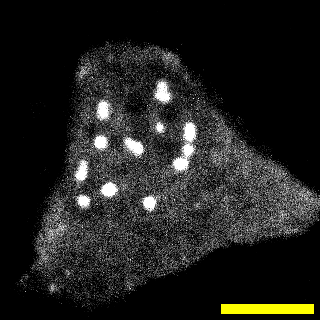

Supplement: Supplementary file 5 — Dataset S4 [file 41467_2024_55089_MOESM5_ESM.zip › Dataset_S4_representative_images/FigureS11_Negative_predictions/NFE4.tif]

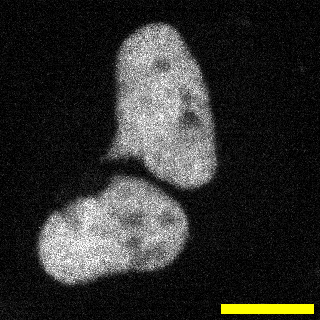

Supplement: Supplementary file 5 — Dataset S4 [file 41467_2024_55089_MOESM5_ESM.zip › Dataset_S4_representative_images/FigureS11_Negative_predictions/ELF5.tif]

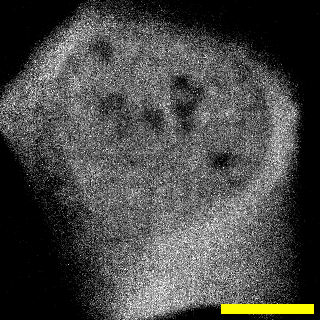

Supplement: Supplementary file 5 — Dataset S4 [file 41467_2024_55089_MOESM5_ESM.zip › Dataset_S4_representative_images/FigureS11_Negative_predictions/C3ORF56.tif]

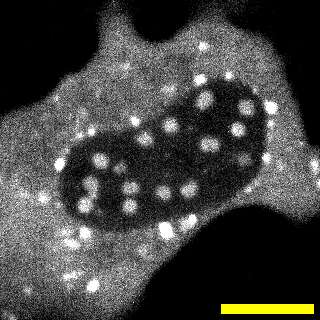

Supplement: Supplementary file 5 — Dataset S4 [file 41467_2024_55089_MOESM5_ESM.zip › Dataset_S4_representative_images/FigureS11_Negative_predictions/DLeu1.tif]

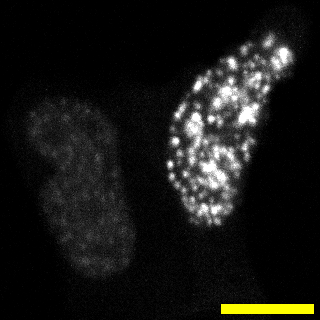

Supplement: Supplementary file 5 — Dataset S4 [file 41467_2024_55089_MOESM5_ESM.zip › Dataset_S4_representative_images/FigureS11_Negative_predictions/INKA1.tif]

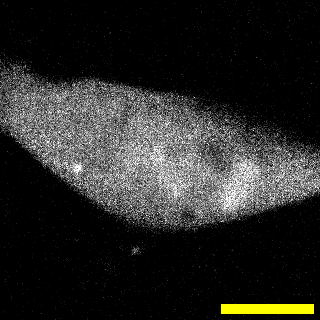

Supplement: Supplementary file 5 — Dataset S4 [file 41467_2024_55089_MOESM5_ESM.zip › Dataset_S4_representative_images/FigureS11_Negative_predictions/STH.tif]

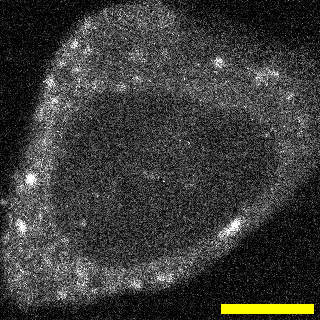

Supplement: Supplementary file 5 — Dataset S4 [file 41467_2024_55089_MOESM5_ESM.zip › Dataset_S4_representative_images/FigureS11_Negative_predictions/PABIR3.tif]

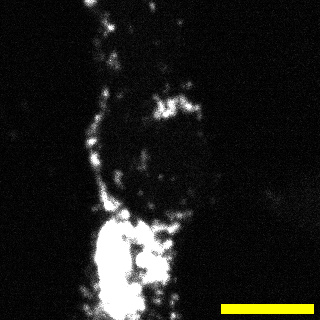

Supplement: Supplementary file 5 — Dataset S4 [file 41467_2024_55089_MOESM5_ESM.zip › Dataset_S4_representative_images/FigureS11_Negative_predictions/BRCC2.tif]

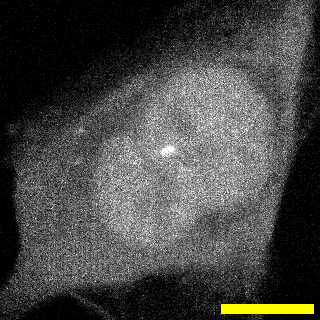

Supplement: Supplementary file 5 — Dataset S4 [file 41467_2024_55089_MOESM5_ESM.zip › Dataset_S4_representative_images/FigureS11_Negative_predictions/C3ORF36.tif]

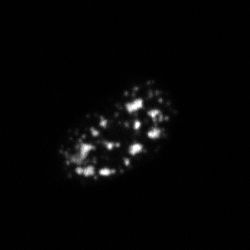

Supplement: Supplementary file 5 — Dataset S4 [file 41467_2024_55089_MOESM5_ESM.zip › Dataset_S4_representative_images/Figure6_FRAP/CWC25.tif]

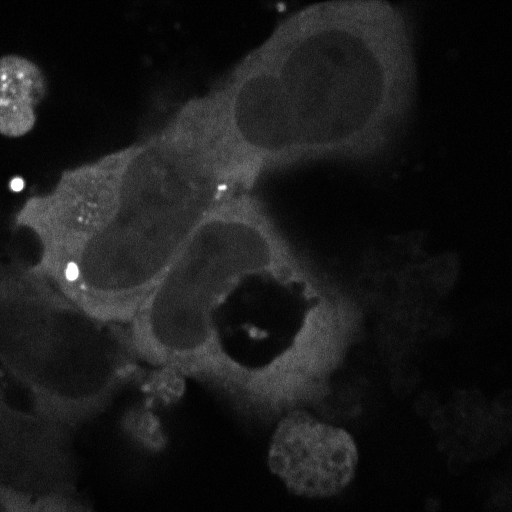

Supplement: Supplementary file 5 — Dataset S4 [file 41467_2024_55089_MOESM5_ESM.zip › Dataset_S4_representative_images/Figure6_FRAP/TYW5.tif]

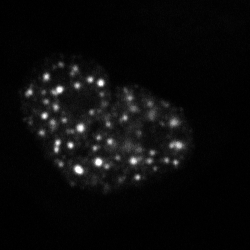

Supplement: Supplementary file 5 — Dataset S4 [file 41467_2024_55089_MOESM5_ESM.zip › Dataset_S4_representative_images/Figure6_FRAP/RP9.tif]

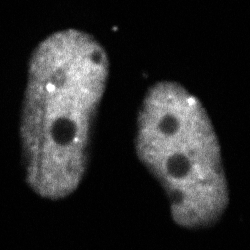

Supplement: Supplementary file 5 — Dataset S4 [file 41467_2024_55089_MOESM5_ESM.zip › Dataset_S4_representative_images/Figure6_FRAP/RBMY1D.tif]

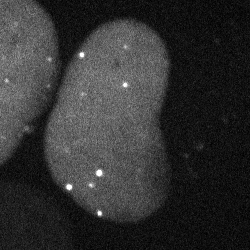

Supplement: Supplementary file 5 — Dataset S4 [file 41467_2024_55089_MOESM5_ESM.zip › Dataset_S4_representative_images/Figure6_FRAP/Rad51AP1.tif]

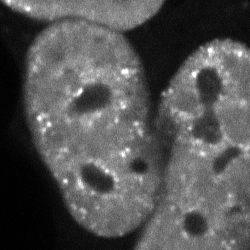

Supplement: Supplementary file 5 — Dataset S4 [file 41467_2024_55089_MOESM5_ESM.zip › Dataset_S4_representative_images/Figure6_FRAP/RAMAC.tif]

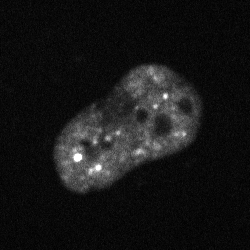

Supplement: Supplementary file 5 — Dataset S4 [file 41467_2024_55089_MOESM5_ESM.zip › Dataset_S4_representative_images/Figure6_FRAP/KHDC4.tif]

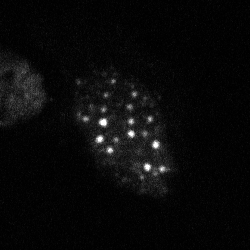

Supplement: Supplementary file 5 — Dataset S4 [file 41467_2024_55089_MOESM5_ESM.zip › Dataset_S4_representative_images/Figure6_FRAP/DRC4.tif]

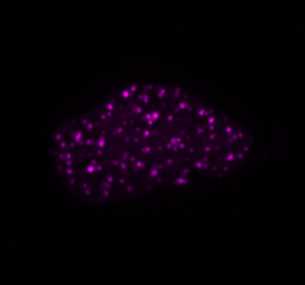

Supplement: Supplementary file 5 — Dataset S4 [file 41467_2024_55089_MOESM5_ESM.zip › Dataset_S4_representative_images/Figure4_Positive_predictions/CWC25.tif]

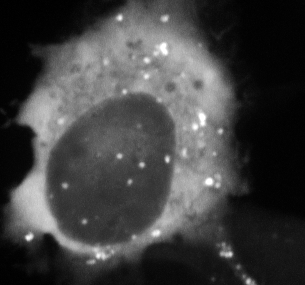

Supplement: Supplementary file 5 — Dataset S4 [file 41467_2024_55089_MOESM5_ESM.zip › Dataset_S4_representative_images/Figure4_Positive_predictions/TYW5.tif]

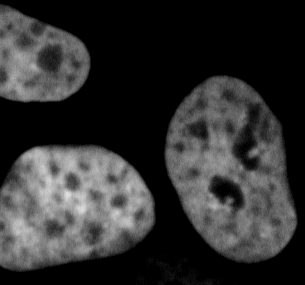

Supplement: Supplementary file 5 — Dataset S4 [file 41467_2024_55089_MOESM5_ESM.zip › Dataset_S4_representative_images/Figure4_Positive_predictions/H1T.tif]

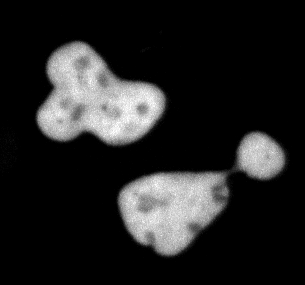

Supplement: Supplementary file 5 — Dataset S4 [file 41467_2024_55089_MOESM5_ESM.zip › Dataset_S4_representative_images/Figure4_Positive_predictions/C1ORF52.tif]

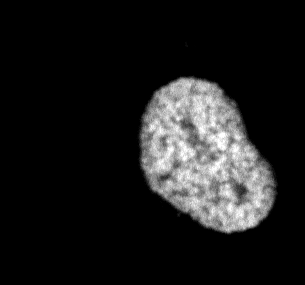

Supplement: Supplementary file 5 — Dataset S4 [file 41467_2024_55089_MOESM5_ESM.zip › Dataset_S4_representative_images/Figure4_Positive_predictions/PolD3.tif]

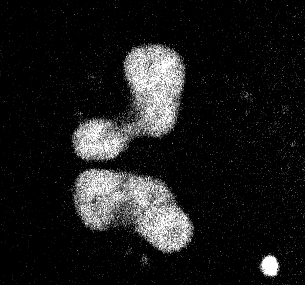

Supplement: Supplementary file 5 — Dataset S4 [file 41467_2024_55089_MOESM5_ESM.zip › Dataset_S4_representative_images/Figure4_Positive_predictions/CWC27.tif]

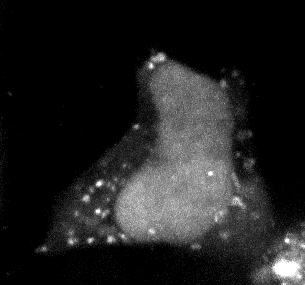

Supplement: Supplementary file 5 — Dataset S4 [file 41467_2024_55089_MOESM5_ESM.zip › Dataset_S4_representative_images/Figure4_Positive_predictions/RS10L.tif]

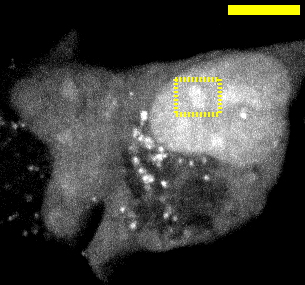

Supplement: Supplementary file 5 — Dataset S4 [file 41467_2024_55089_MOESM5_ESM.zip › Dataset_S4_representative_images/Figure4_Positive_predictions/MRPL1.tif]

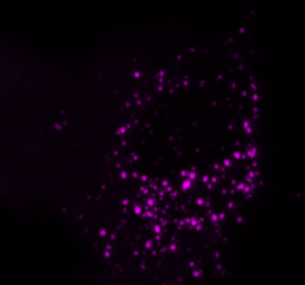

Supplement: Supplementary file 5 — Dataset S4 [file 41467_2024_55089_MOESM5_ESM.zip › Dataset_S4_representative_images/Figure4_Positive_predictions/SPA24.tif]

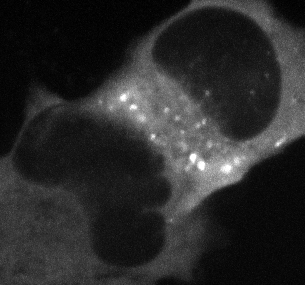

Supplement: Supplementary file 5 — Dataset S4 [file 41467_2024_55089_MOESM5_ESM.zip › Dataset_S4_representative_images/Figure4_Positive_predictions/ZC3H15.tif]

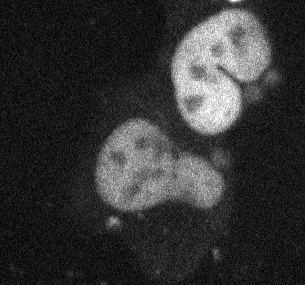

Supplement: Supplementary file 5 — Dataset S4 [file 41467_2024_55089_MOESM5_ESM.zip › Dataset_S4_representative_images/Figure4_Positive_predictions/RPS4Y2.tif]

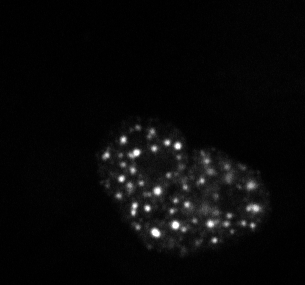

Supplement: Supplementary file 5 — Dataset S4 [file 41467_2024_55089_MOESM5_ESM.zip › Dataset_S4_representative_images/Figure4_Positive_predictions/RP9.tif]

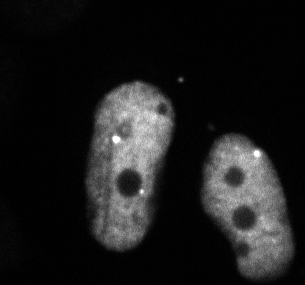

Supplement: Supplementary file 5 — Dataset S4 [file 41467_2024_55089_MOESM5_ESM.zip › Dataset_S4_representative_images/Figure4_Positive_predictions/RBMY1D.tif]

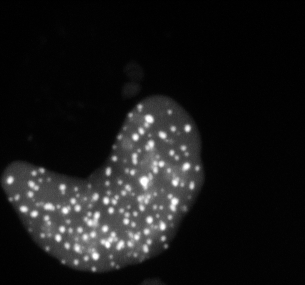

Supplement: Supplementary file 5 — Dataset S4 [file 41467_2024_55089_MOESM5_ESM.zip › Dataset_S4_representative_images/Figure4_Positive_predictions/Rad51AP1.tif]

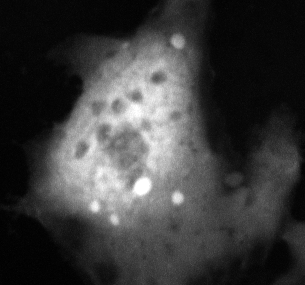

Supplement: Supplementary file 5 — Dataset S4 [file 41467_2024_55089_MOESM5_ESM.zip › Dataset_S4_representative_images/Figure4_Positive_predictions/PHP14.tif]

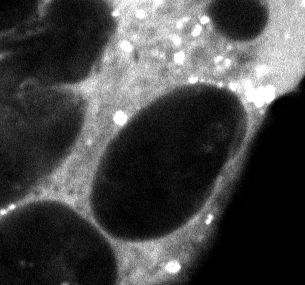

Supplement: Supplementary file 5 — Dataset S4 [file 41467_2024_55089_MOESM5_ESM.zip › Dataset_S4_representative_images/Figure4_Positive_predictions/LMOD1.tif]

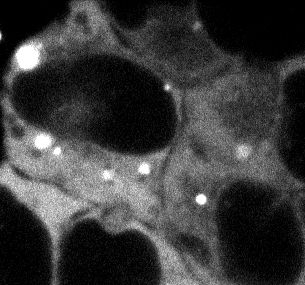

Supplement: Supplementary file 5 — Dataset S4 [file 41467_2024_55089_MOESM5_ESM.zip › Dataset_S4_representative_images/Figure4_Positive_predictions/HBS1L.tif]

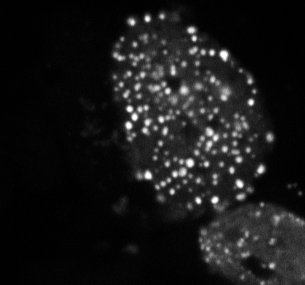

Supplement: Supplementary file 5 — Dataset S4 [file 41467_2024_55089_MOESM5_ESM.zip › Dataset_S4_representative_images/Figure4_Positive_predictions/RAMAC.tif]

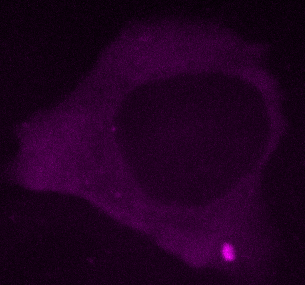

Supplement: Supplementary file 5 — Dataset S4 [file 41467_2024_55089_MOESM5_ESM.zip › Dataset_S4_representative_images/Figure4_Positive_predictions/eIF2GA.tif]

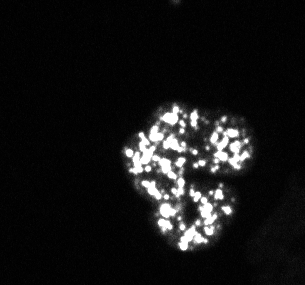

Supplement: Supplementary file 5 — Dataset S4 [file 41467_2024_55089_MOESM5_ESM.zip › Dataset_S4_representative_images/Figure4_Positive_predictions/KHDC4.tif]

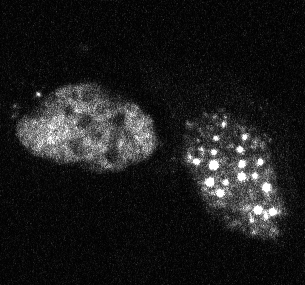

Supplement: Supplementary file 5 — Dataset S4 [file 41467_2024_55089_MOESM5_ESM.zip › Dataset_S4_representative_images/Figure4_Positive_predictions/DRC4.tif]

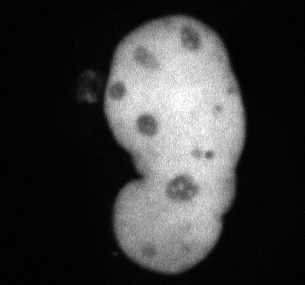

Supplement: Supplementary file 5 — Dataset S4 [file 41467_2024_55089_MOESM5_ESM.zip › Dataset_S4_representative_images/Figure4_Positive_predictions/SPAG7.tif]

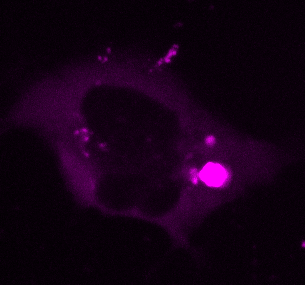

Supplement: Supplementary file 5 — Dataset S4 [file 41467_2024_55089_MOESM5_ESM.zip › Dataset_S4_representative_images/Figure4_Positive_predictions/AIMP1.tif]

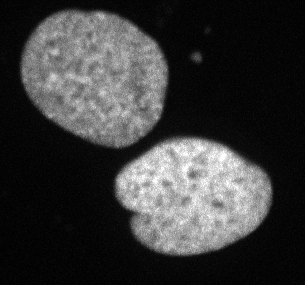

Supplement: Supplementary file 5 — Dataset S4 [file 41467_2024_55089_MOESM5_ESM.zip › Dataset_S4_representative_images/Figure4_Positive_predictions/H2A1H.tif]

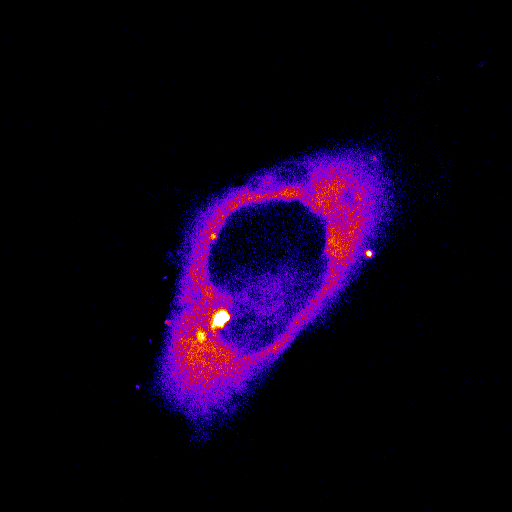

Supplement: Supplementary file 5 — Dataset S4 [file 41467_2024_55089_MOESM5_ESM.zip › Dataset_S4_representative_images/FigureS10_Representative Images/AIMP1/10.tif]

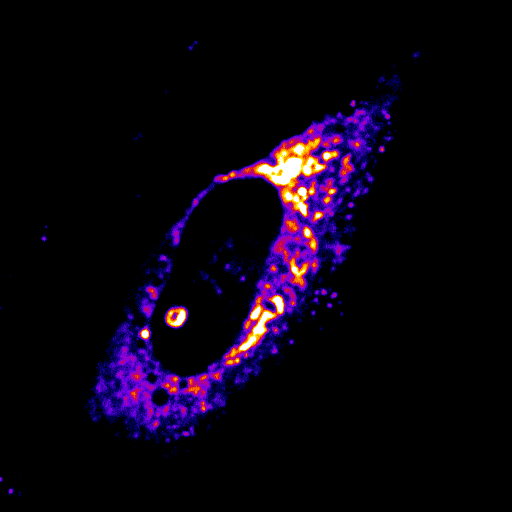

Supplement: Supplementary file 5 — Dataset S4 [file 41467_2024_55089_MOESM5_ESM.zip › Dataset_S4_representative_images/FigureS10_Representative Images/AIMP1/9.tif]

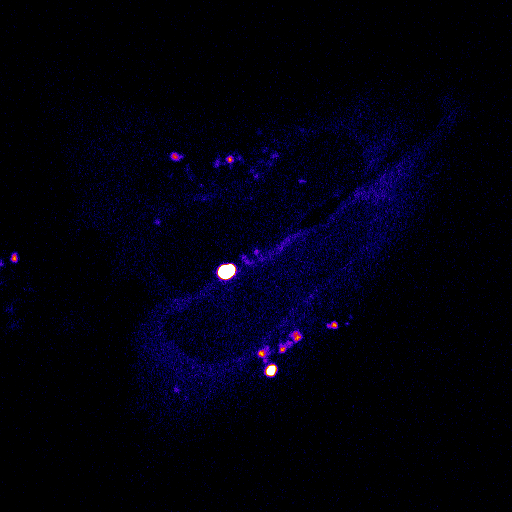

Supplement: Supplementary file 5 — Dataset S4 [file 41467_2024_55089_MOESM5_ESM.zip › Dataset_S4_representative_images/FigureS10_Representative Images/AIMP1/8.tif]

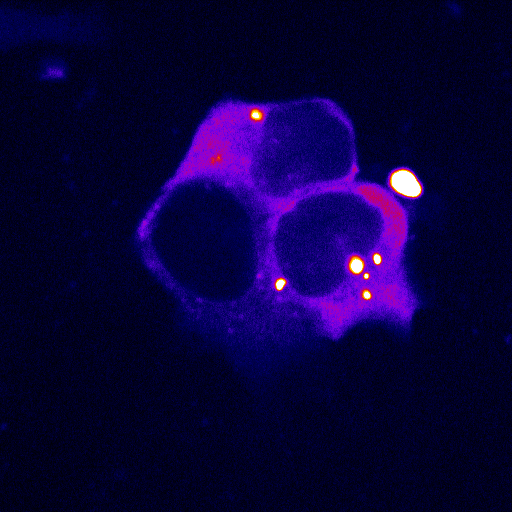

Supplement: Supplementary file 5 — Dataset S4 [file 41467_2024_55089_MOESM5_ESM.zip › Dataset_S4_representative_images/FigureS10_Representative Images/AIMP1/3.tif]

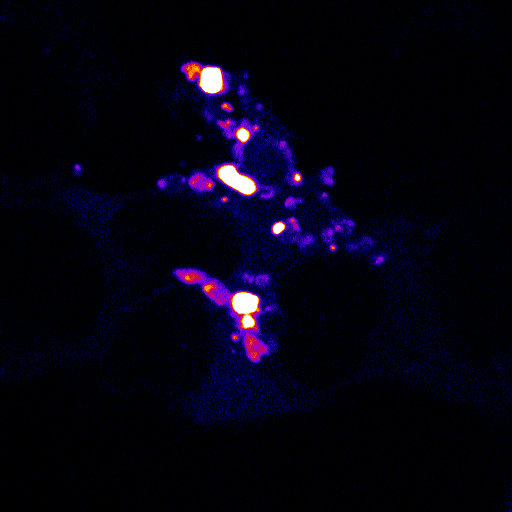

Supplement: Supplementary file 5 — Dataset S4 [file 41467_2024_55089_MOESM5_ESM.zip › Dataset_S4_representative_images/FigureS10_Representative Images/AIMP1/2.tif]

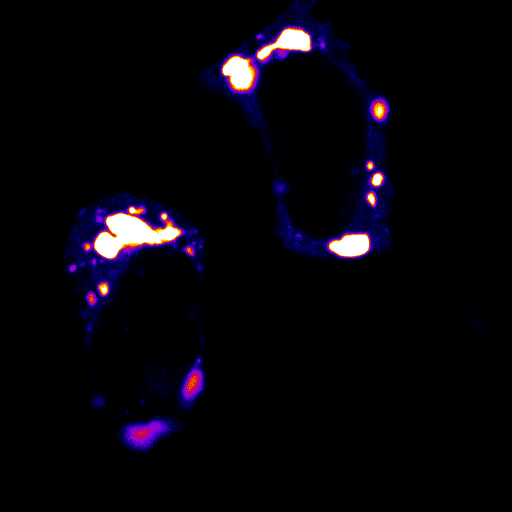

Supplement: Supplementary file 5 — Dataset S4 [file 41467_2024_55089_MOESM5_ESM.zip › Dataset_S4_representative_images/FigureS10_Representative Images/AIMP1/1.tif]

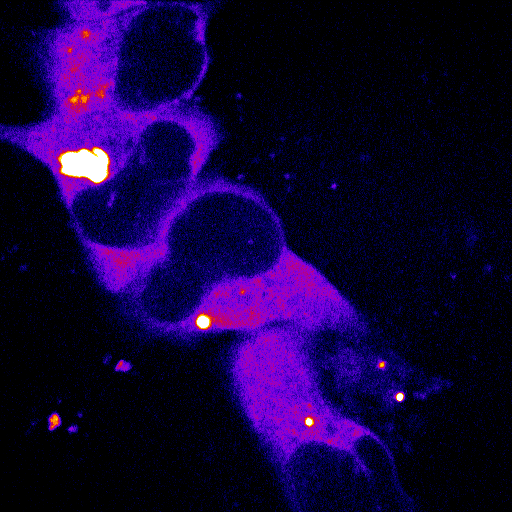

Supplement: Supplementary file 5 — Dataset S4 [file 41467_2024_55089_MOESM5_ESM.zip › Dataset_S4_representative_images/FigureS10_Representative Images/AIMP1/5.tif]

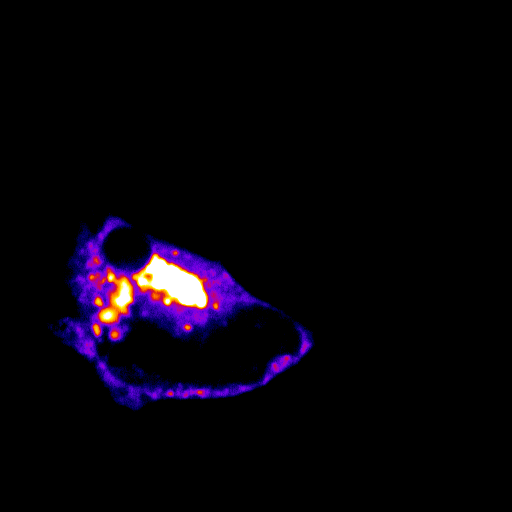

Supplement: Supplementary file 5 — Dataset S4 [file 41467_2024_55089_MOESM5_ESM.zip › Dataset_S4_representative_images/FigureS10_Representative Images/AIMP1/4.tif]

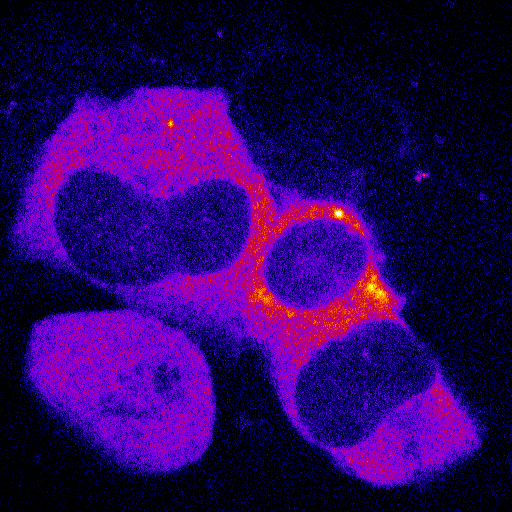

Supplement: Supplementary file 5 — Dataset S4 [file 41467_2024_55089_MOESM5_ESM.zip › Dataset_S4_representative_images/FigureS10_Representative Images/AIMP1/6.tif]

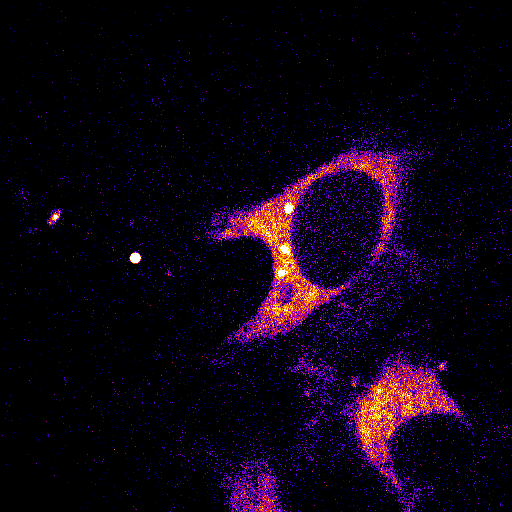

Supplement: Supplementary file 5 — Dataset S4 [file 41467_2024_55089_MOESM5_ESM.zip › Dataset_S4_representative_images/FigureS10_Representative Images/AIMP1/7.tif]

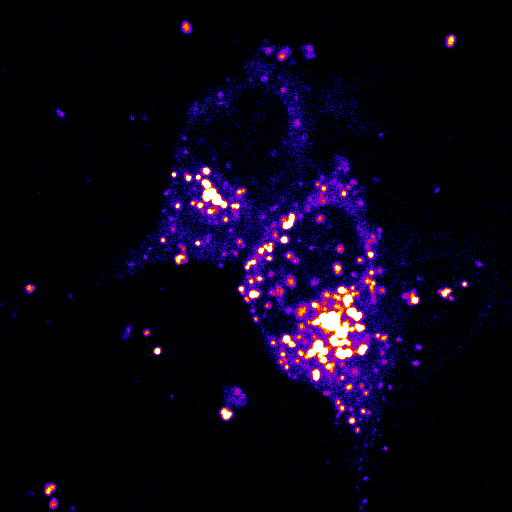

Supplement: Supplementary file 5 — Dataset S4 [file 41467_2024_55089_MOESM5_ESM.zip › Dataset_S4_representative_images/FigureS10_Representative Images/DRC4/10.tif]

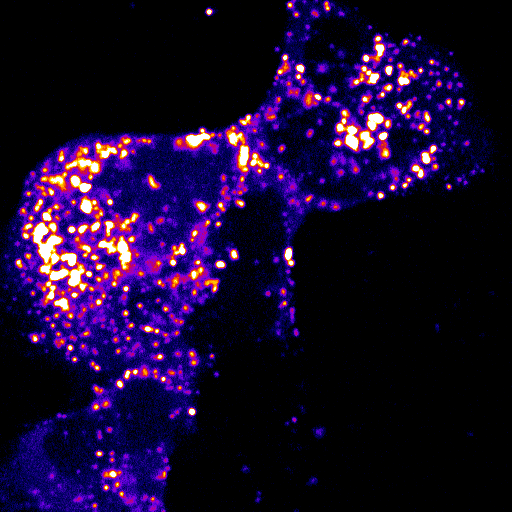

Supplement: Supplementary file 5 — Dataset S4 [file 41467_2024_55089_MOESM5_ESM.zip › Dataset_S4_representative_images/FigureS10_Representative Images/DRC4/9.tif]

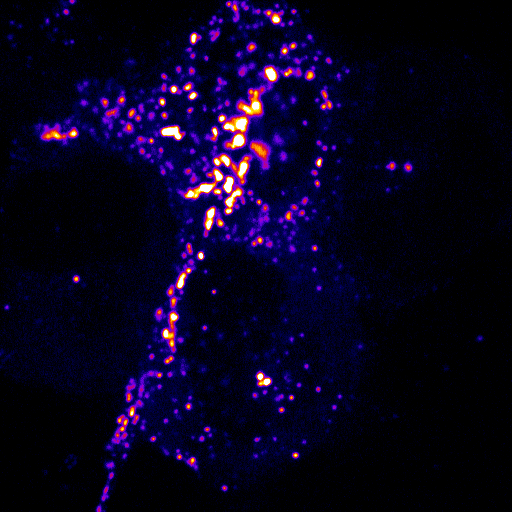

Supplement: Supplementary file 5 — Dataset S4 [file 41467_2024_55089_MOESM5_ESM.zip › Dataset_S4_representative_images/FigureS10_Representative Images/DRC4/8.tif]

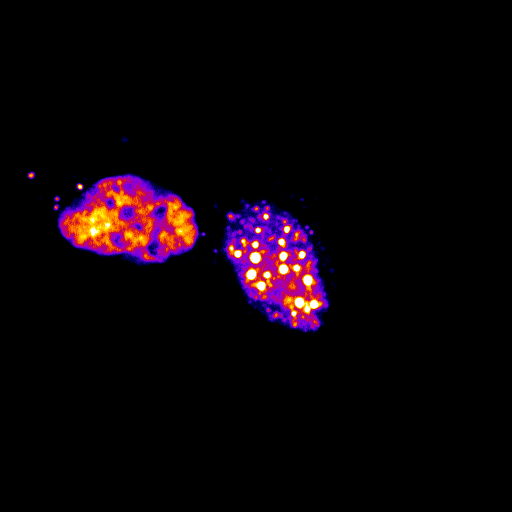

Supplement: Supplementary file 5 — Dataset S4 [file 41467_2024_55089_MOESM5_ESM.zip › Dataset_S4_representative_images/FigureS10_Representative Images/DRC4/3.tif]

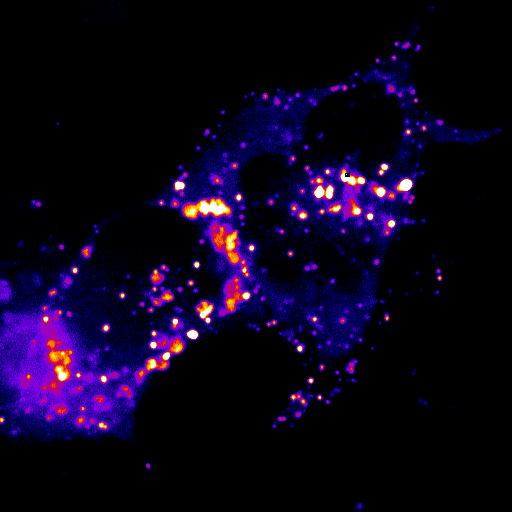

Supplement: Supplementary file 5 — Dataset S4 [file 41467_2024_55089_MOESM5_ESM.zip › Dataset_S4_representative_images/FigureS10_Representative Images/DRC4/2.tif]

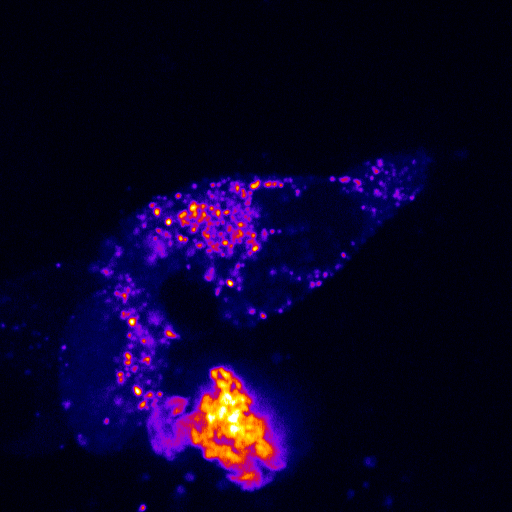

Supplement: Supplementary file 5 — Dataset S4 [file 41467_2024_55089_MOESM5_ESM.zip › Dataset_S4_representative_images/FigureS10_Representative Images/DRC4/1.tif]

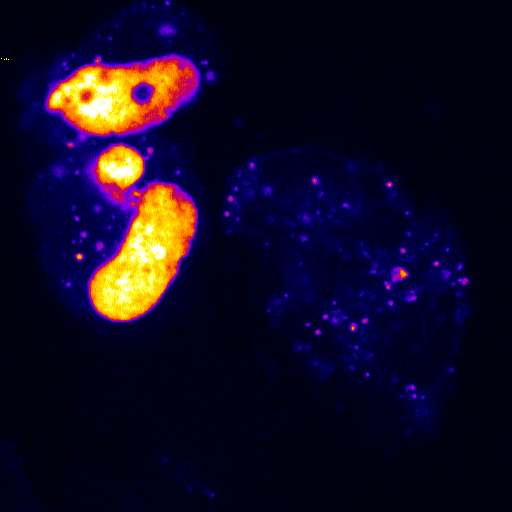

Supplement: Supplementary file 5 — Dataset S4 [file 41467_2024_55089_MOESM5_ESM.zip › Dataset_S4_representative_images/FigureS10_Representative Images/DRC4/5.tif]

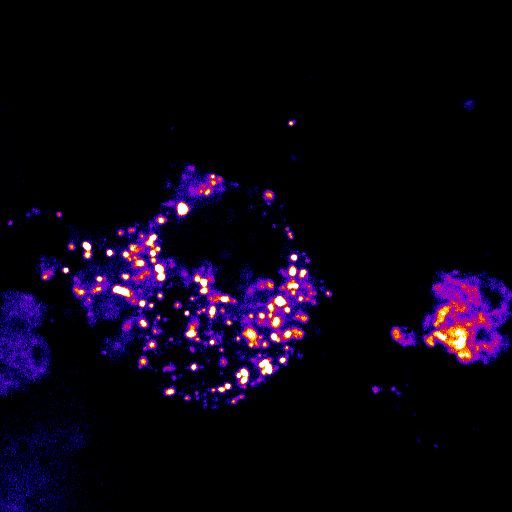

Supplement: Supplementary file 5 — Dataset S4 [file 41467_2024_55089_MOESM5_ESM.zip › Dataset_S4_representative_images/FigureS10_Representative Images/DRC4/4.tif]

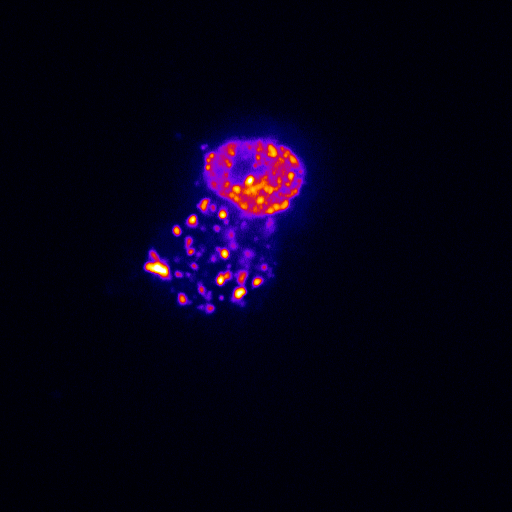

Supplement: Supplementary file 5 — Dataset S4 [file 41467_2024_55089_MOESM5_ESM.zip › Dataset_S4_representative_images/FigureS10_Representative Images/DRC4/6.tif]

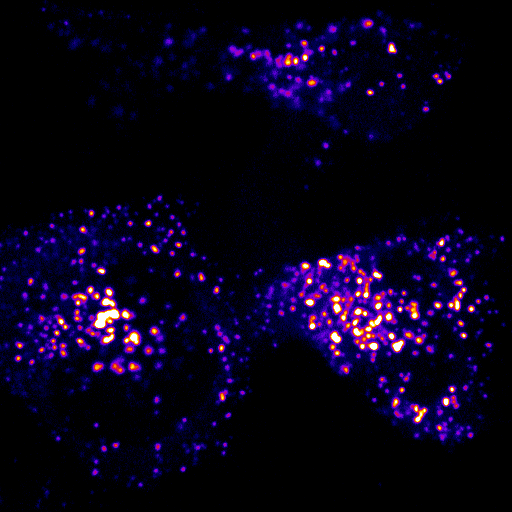

Supplement: Supplementary file 5 — Dataset S4 [file 41467_2024_55089_MOESM5_ESM.zip › Dataset_S4_representative_images/FigureS10_Representative Images/DRC4/7.tif]

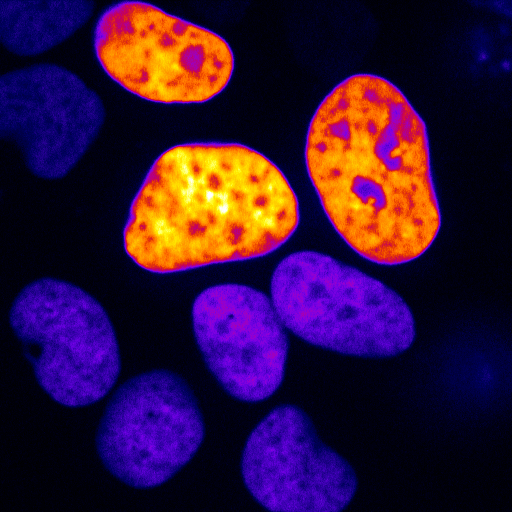

Supplement: Supplementary file 5 — Dataset S4 [file 41467_2024_55089_MOESM5_ESM.zip › Dataset_S4_representative_images/FigureS10_Representative Images/H1T/10.tif]

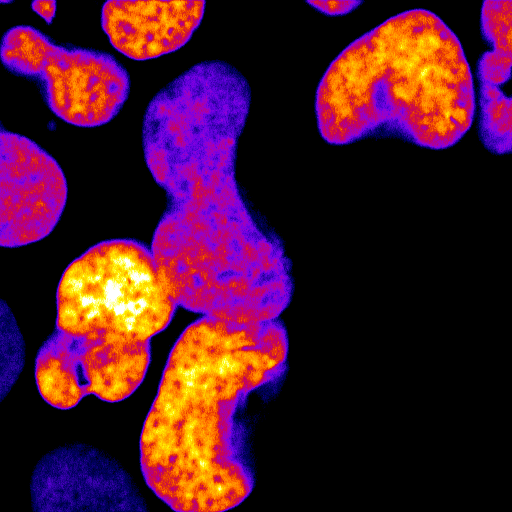

Supplement: Supplementary file 5 — Dataset S4 [file 41467_2024_55089_MOESM5_ESM.zip › Dataset_S4_representative_images/FigureS10_Representative Images/H1T/9.tif]

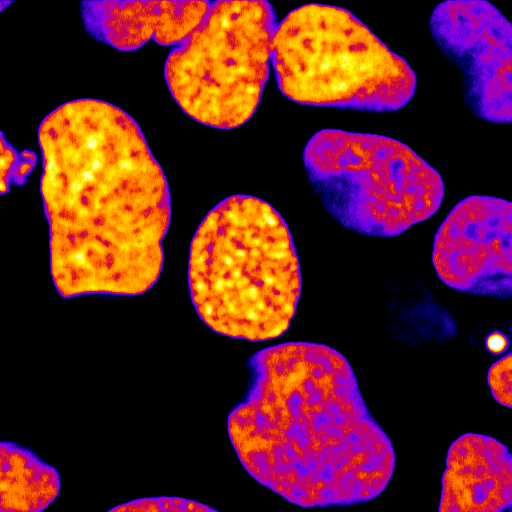

Supplement: Supplementary file 5 — Dataset S4 [file 41467_2024_55089_MOESM5_ESM.zip › Dataset_S4_representative_images/FigureS10_Representative Images/H1T/8.tif]

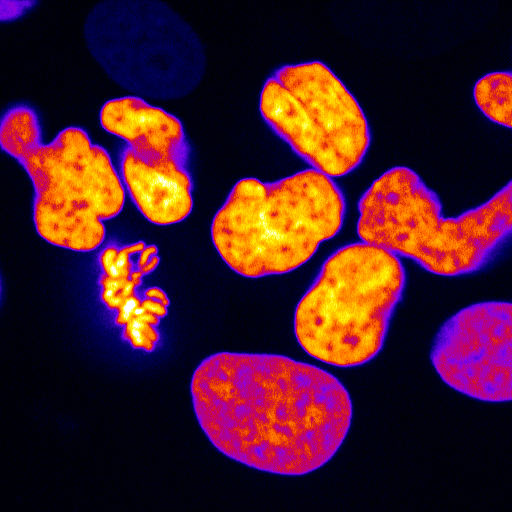

Supplement: Supplementary file 5 — Dataset S4 [file 41467_2024_55089_MOESM5_ESM.zip › Dataset_S4_representative_images/FigureS10_Representative Images/H1T/3.tif]

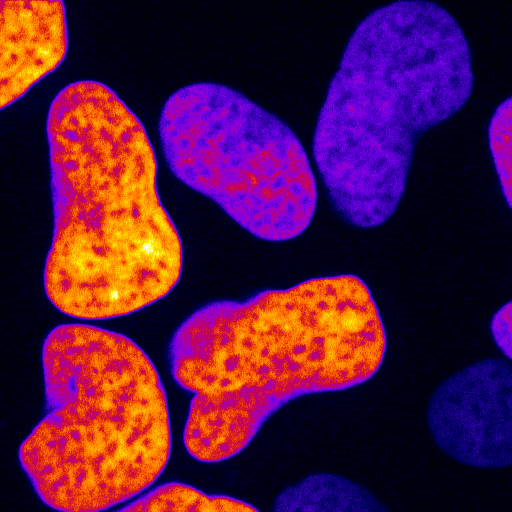

Supplement: Supplementary file 5 — Dataset S4 [file 41467_2024_55089_MOESM5_ESM.zip › Dataset_S4_representative_images/FigureS10_Representative Images/H1T/2.tif]

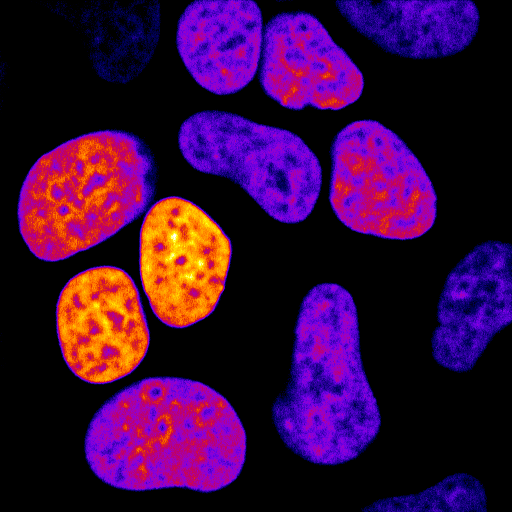

Supplement: Supplementary file 5 — Dataset S4 [file 41467_2024_55089_MOESM5_ESM.zip › Dataset_S4_representative_images/FigureS10_Representative Images/H1T/1.tif]

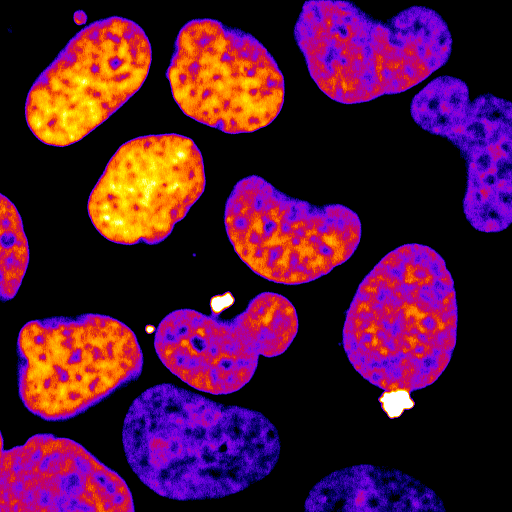

Supplement: Supplementary file 5 — Dataset S4 [file 41467_2024_55089_MOESM5_ESM.zip › Dataset_S4_representative_images/FigureS10_Representative Images/H1T/5.tif]

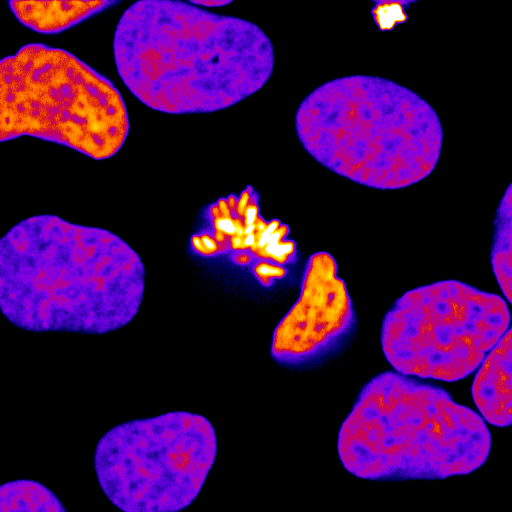

Supplement: Supplementary file 5 — Dataset S4 [file 41467_2024_55089_MOESM5_ESM.zip › Dataset_S4_representative_images/FigureS10_Representative Images/H1T/4.tif]

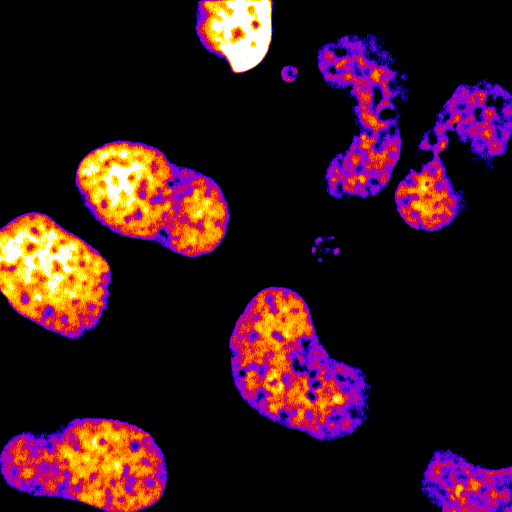

Supplement: Supplementary file 5 — Dataset S4 [file 41467_2024_55089_MOESM5_ESM.zip › Dataset_S4_representative_images/FigureS10_Representative Images/H1T/6.tif]

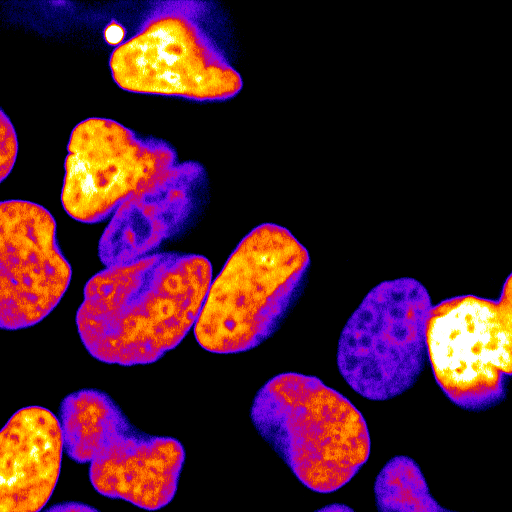

Supplement: Supplementary file 5 — Dataset S4 [file 41467_2024_55089_MOESM5_ESM.zip › Dataset_S4_representative_images/FigureS10_Representative Images/H1T/7.tif]

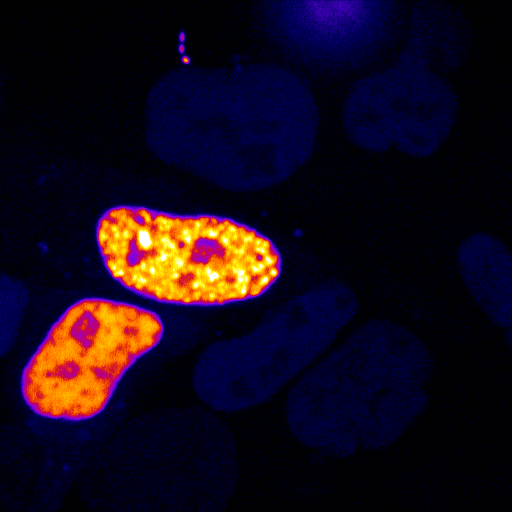

Supplement: Supplementary file 5 — Dataset S4 [file 41467_2024_55089_MOESM5_ESM.zip › Dataset_S4_representative_images/FigureS10_Representative Images/PolD3/10.tif]

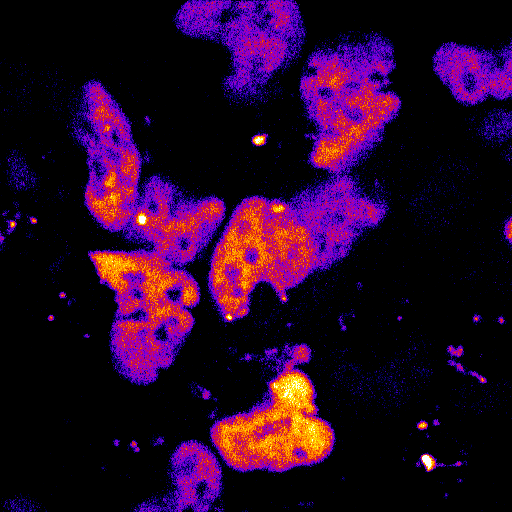

Supplement: Supplementary file 5 — Dataset S4 [file 41467_2024_55089_MOESM5_ESM.zip › Dataset_S4_representative_images/FigureS10_Representative Images/PolD3/11.tif]

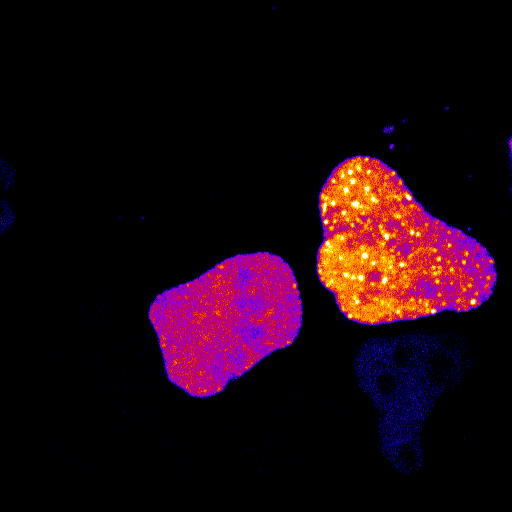

Supplement: Supplementary file 5 — Dataset S4 [file 41467_2024_55089_MOESM5_ESM.zip › Dataset_S4_representative_images/FigureS10_Representative Images/PolD3/9.tif]

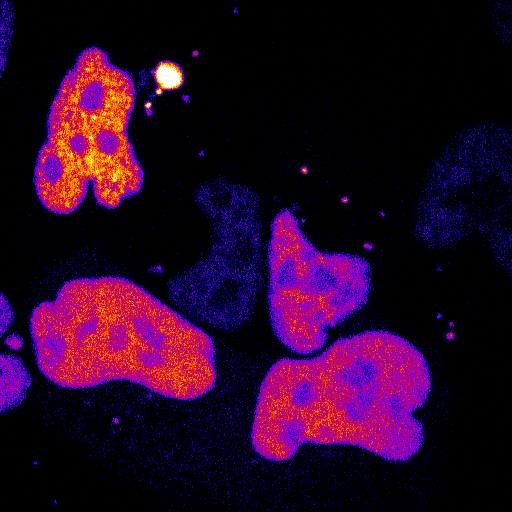

Supplement: Supplementary file 5 — Dataset S4 [file 41467_2024_55089_MOESM5_ESM.zip › Dataset_S4_representative_images/FigureS10_Representative Images/PolD3/8.tif]

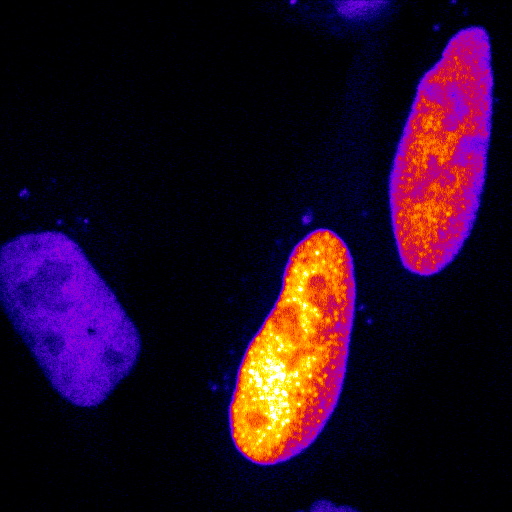

Supplement: Supplementary file 5 — Dataset S4 [file 41467_2024_55089_MOESM5_ESM.zip › Dataset_S4_representative_images/FigureS10_Representative Images/PolD3/3.tif]

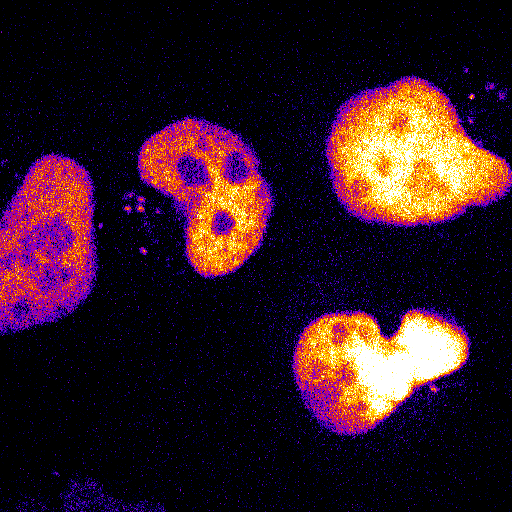

Supplement: Supplementary file 5 — Dataset S4 [file 41467_2024_55089_MOESM5_ESM.zip › Dataset_S4_representative_images/FigureS10_Representative Images/PolD3/2.tif]

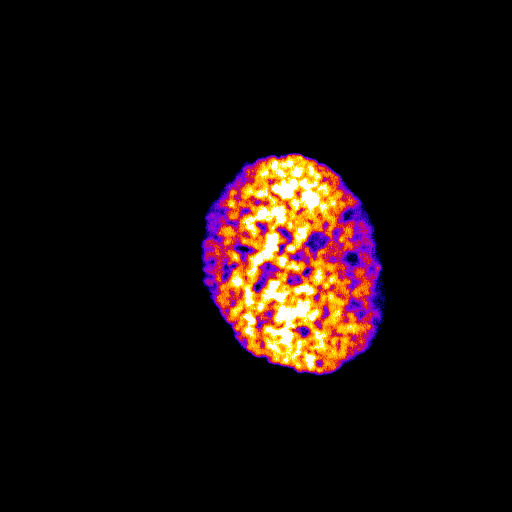

Supplement: Supplementary file 5 — Dataset S4 [file 41467_2024_55089_MOESM5_ESM.zip › Dataset_S4_representative_images/FigureS10_Representative Images/PolD3/1.tif]

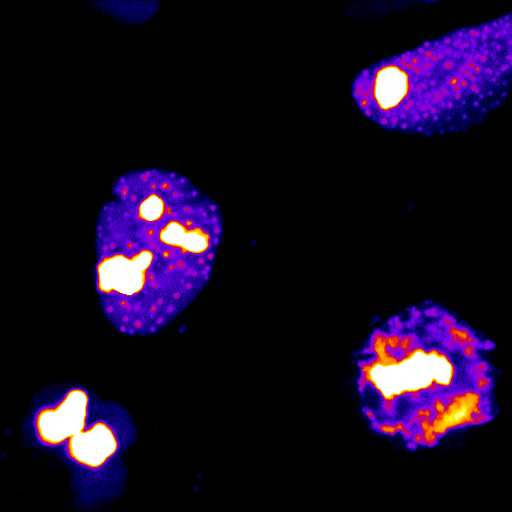

Supplement: Supplementary file 5 — Dataset S4 [file 41467_2024_55089_MOESM5_ESM.zip › Dataset_S4_representative_images/FigureS10_Representative Images/PolD3/5.tif]

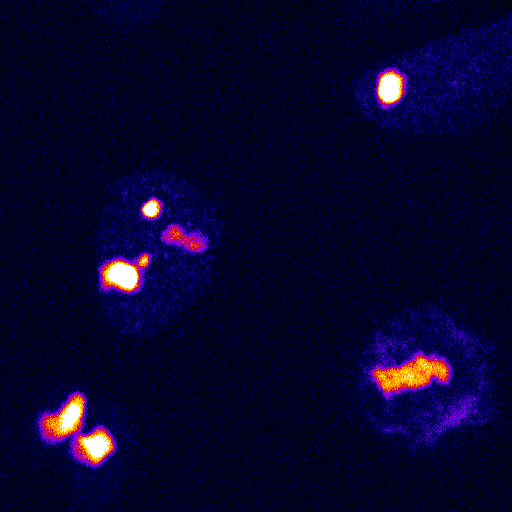

Supplement: Supplementary file 5 — Dataset S4 [file 41467_2024_55089_MOESM5_ESM.zip › Dataset_S4_representative_images/FigureS10_Representative Images/PolD3/4.tif]

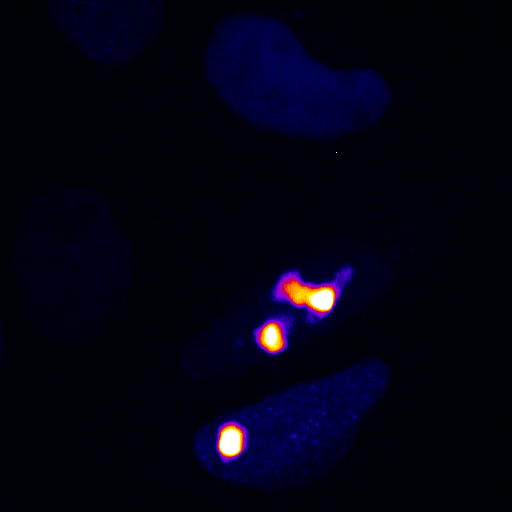

Supplement: Supplementary file 5 — Dataset S4 [file 41467_2024_55089_MOESM5_ESM.zip › Dataset_S4_representative_images/FigureS10_Representative Images/PolD3/6.tif]

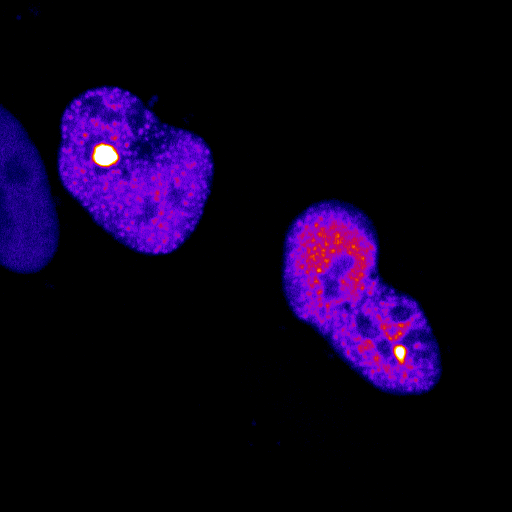

Supplement: Supplementary file 5 — Dataset S4 [file 41467_2024_55089_MOESM5_ESM.zip › Dataset_S4_representative_images/FigureS10_Representative Images/PolD3/7.tif]

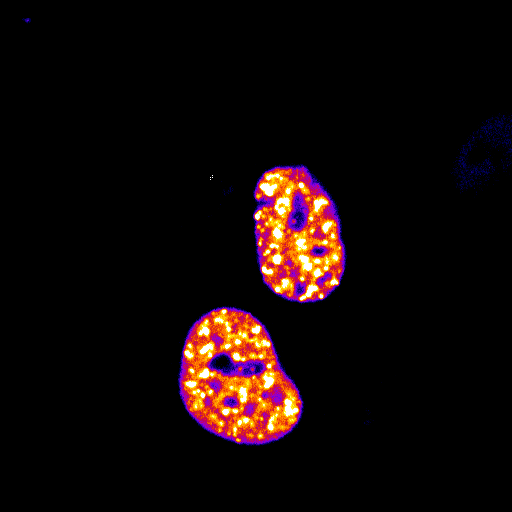

Supplement: Supplementary file 5 — Dataset S4 [file 41467_2024_55089_MOESM5_ESM.zip › Dataset_S4_representative_images/FigureS10_Representative Images/RAMAC/13.tif]

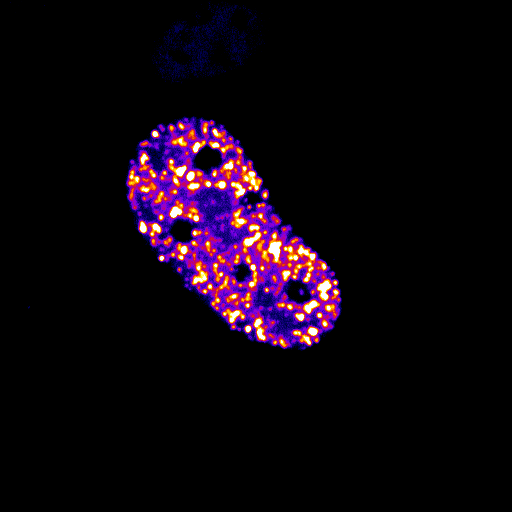

Supplement: Supplementary file 5 — Dataset S4 [file 41467_2024_55089_MOESM5_ESM.zip › Dataset_S4_representative_images/FigureS10_Representative Images/RAMAC/12.tif]

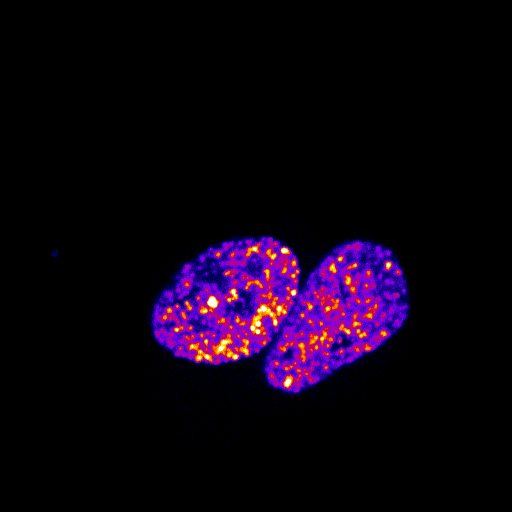

Supplement: Supplementary file 5 — Dataset S4 [file 41467_2024_55089_MOESM5_ESM.zip › Dataset_S4_representative_images/FigureS10_Representative Images/RAMAC/10.tif]

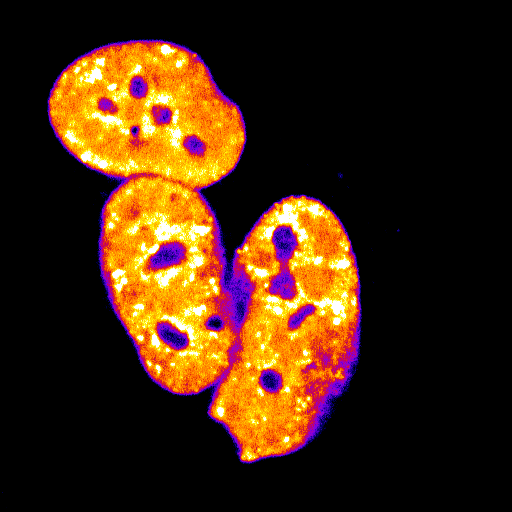

Supplement: Supplementary file 5 — Dataset S4 [file 41467_2024_55089_MOESM5_ESM.zip › Dataset_S4_representative_images/FigureS10_Representative Images/RAMAC/11.tif]

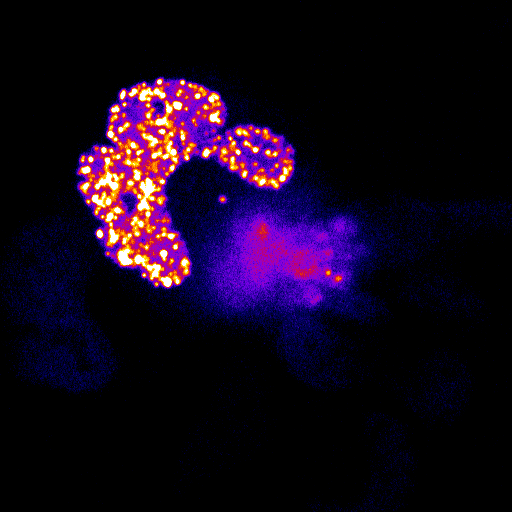

Supplement: Supplementary file 5 — Dataset S4 [file 41467_2024_55089_MOESM5_ESM.zip › Dataset_S4_representative_images/FigureS10_Representative Images/RAMAC/14.tif]

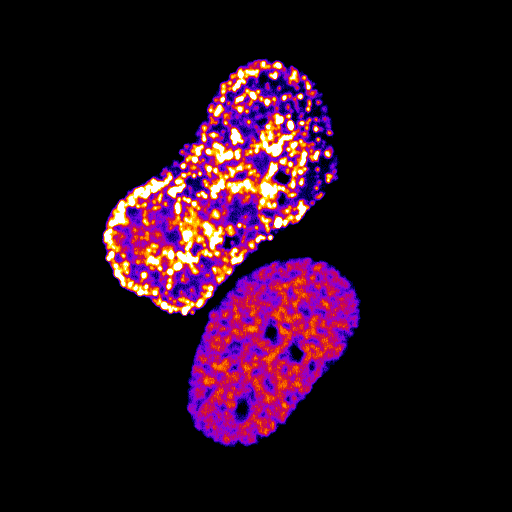

Supplement: Supplementary file 5 — Dataset S4 [file 41467_2024_55089_MOESM5_ESM.zip › Dataset_S4_representative_images/FigureS10_Representative Images/RAMAC/9.tif]

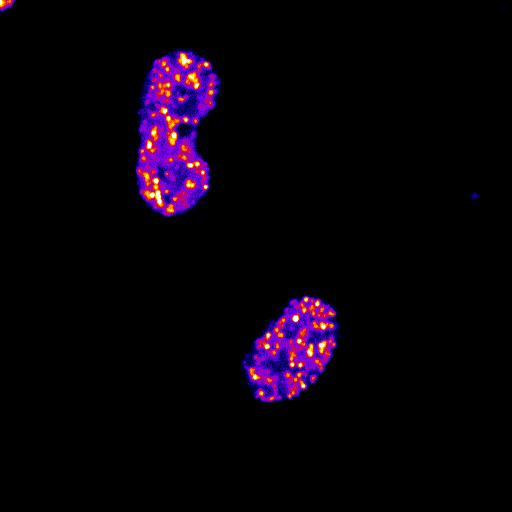

Supplement: Supplementary file 5 — Dataset S4 [file 41467_2024_55089_MOESM5_ESM.zip › Dataset_S4_representative_images/FigureS10_Representative Images/RAMAC/8.tif]

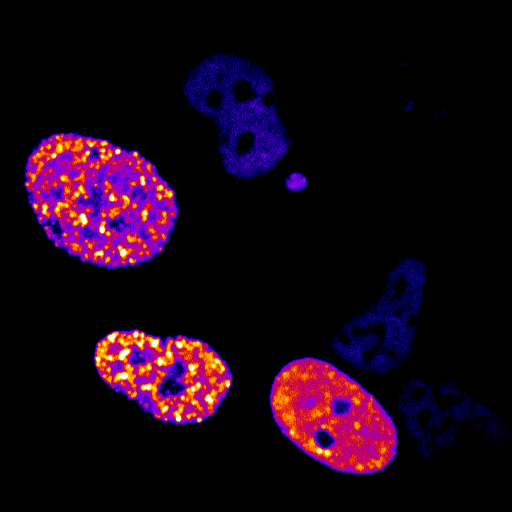

Supplement: Supplementary file 5 — Dataset S4 [file 41467_2024_55089_MOESM5_ESM.zip › Dataset_S4_representative_images/FigureS10_Representative Images/RAMAC/3.tif]

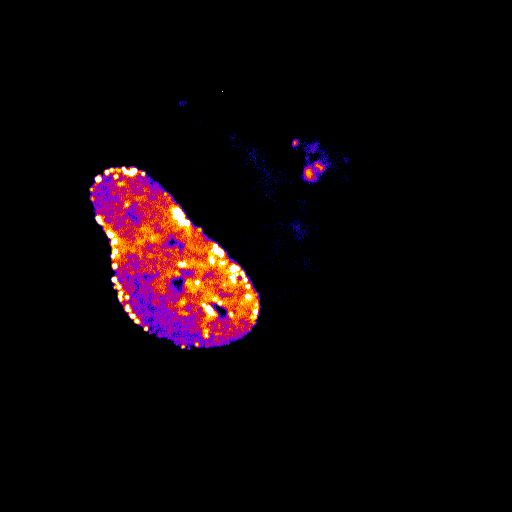

Supplement: Supplementary file 5 — Dataset S4 [file 41467_2024_55089_MOESM5_ESM.zip › Dataset_S4_representative_images/FigureS10_Representative Images/RAMAC/2.tif]

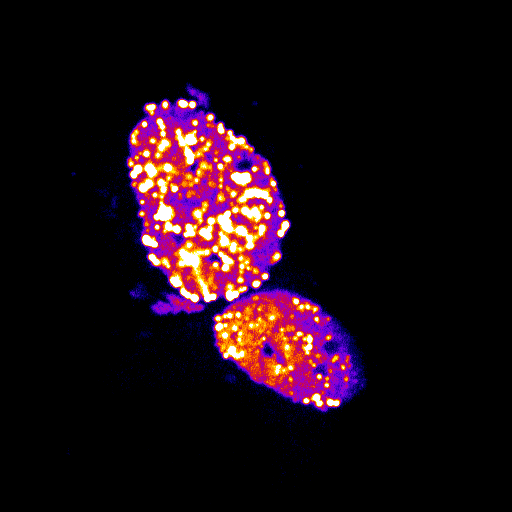

Supplement: Supplementary file 5 — Dataset S4 [file 41467_2024_55089_MOESM5_ESM.zip › Dataset_S4_representative_images/FigureS10_Representative Images/RAMAC/1.tif]

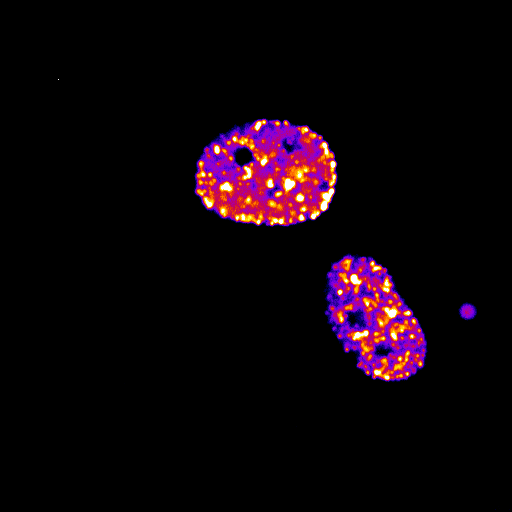

Supplement: Supplementary file 5 — Dataset S4 [file 41467_2024_55089_MOESM5_ESM.zip › Dataset_S4_representative_images/FigureS10_Representative Images/RAMAC/5.tif]

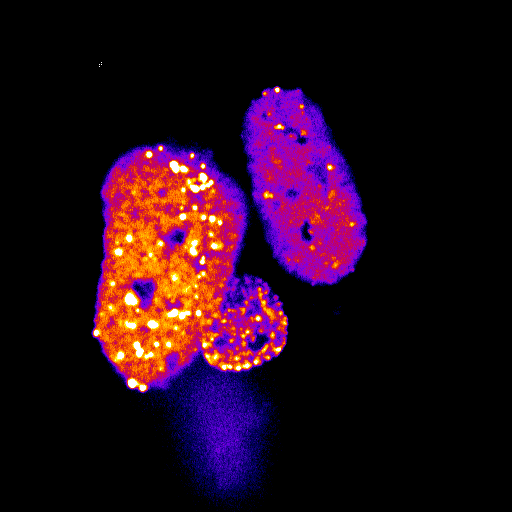

Supplement: Supplementary file 5 — Dataset S4 [file 41467_2024_55089_MOESM5_ESM.zip › Dataset_S4_representative_images/FigureS10_Representative Images/RAMAC/4.tif]
