# Supplementary material for: PICNIC accurately predicts condensate-forming proteins regardless of their structural disorder across organisms
Source: Nat Commun. 2024 Dec 11;15:10668. doi: 10.1038/s41467-024-55089-x (PMC11634905; doi:10.1038/s41467-024-55089-x)
Supplement: Supplementary file 8 — Reporting Summary [file 41467_2024_55089_MOESM8_ESM.pdf]

nature portfolio

Corresponding author(s): Agnes Toth-Petroczy

Last updated by author(s): Nov 18, 2024

Reporting Summary

Nature Portfolio wishes to improve the reproducibility of the work that we publish. This form provides structure for consistency and transparency in reporting. For further information on Nature Portfolio policies, see our Editorial Policies and the Editorial Policy Checklist.

Statistics

For all statistical analyses, confirm that the following items are present in the figure legend, table legend, main text, or Methods section.

n/a	Confirmed	
☐	☒	The exact sample size (*n*) for each experimental group/condition, given as a discrete number and unit of measurement
☒	☐	A statement on whether measurements were taken from distinct samples or whether the same sample was measured repeatedly
☒	☐	The statistical test(s) used AND whether they are one- or two-sided<br>*Only common tests should be described solely by name; describe more complex techniques in the Methods section.*
☐	☒	A description of all covariates tested
☒	☐	A description of any assumptions or corrections, such as tests of normality and adjustment for multiple comparisons
☐	☒	A full description of the statistical parameters including central tendency (e.g. means) or other basic estimates (e.g. regression coefficient) AND variation (e.g. standard deviation) or associated estimates of uncertainty (e.g. confidence intervals)
☒	☐	For null hypothesis testing, the test statistic (e.g. *F*, *t*, *r*) with confidence intervals, effect sizes, degrees of freedom and *P* value noted<br>*Give P values as exact values whenever suitable.*
☒	☐	For Bayesian analysis, information on the choice of priors and Markov chain Monte Carlo settings
☒	☐	For hierarchical and complex designs, identification of the appropriate level for tests and full reporting of outcomes
☐	☒	Estimates of effect sizes (e.g. Cohen's *d*, Pearson's *r*), indicating how they were calculated

Our web collection on statistics for biologists contains articles on many of the points above.

Software and code

Policy information about availability of computer code

Data collection	*Provide a description of all commercial, open source and custom code used to collect the data in this study, specifying the version used OR state that no software was used.*
Data analysis	IUPRED2a (https://iupred2a.elte.hu/); Python (version 3.8.8) with packages Catboost (0.26.1), Numpy (1.20.1), Biopython (1.78), Pandas (1.2.4), Matplotlib (3.3.4), Pickle (4.0), Xgboost (1.4.2), Stride (0.1.6), Scipy (1.6.2), Scikit-learn (0.24.1), ProDy (2.0.1), Lightgbm (3.3.0). PyMOL software (https://pymol.org/2/) was used for protein structure visualization and analysis (version 2.4.2). Fiji_2021, GraphPad Prism10

For manuscripts utilizing custom algorithms or software that are central to the research but not yet described in published literature, software must be made available to editors and reviewers. We strongly encourage code deposition in a community repository (e.g. GitHub). See the Nature Portfolio guidelines for submitting code & software for further information.

Data

Policy information about availability of data

All manuscripts must include a data availability statement. This statement should provide the following information, where applicable:

- Accession codes, unique identifiers, or web links for publicly available datasets
- A description of any restrictions on data availability
- For clinical datasets or third party data, please ensure that the statement adheres to our policy

Alphafold database (https://alphafold.ebi.ac.uk/), second release, date of access: January, 2022 - for protein structures predicted by Alphafold2 algorithm

InWeb database (version v3) - for protein-protein interaction network to build negative dataset
Uniprot (https://www.uniprot.org/), release 2021_04 - for retrieval of protein sequences and Gene Ontology annotation for human, release 2022_03 - for other species
CD-CODE database of biomolecular condensates (https://cd-code.org/about), v1.0 - for annotation of condensate-forming proteins.
TThe training, validation and test datasets are available as Dataset S1. The list and properties of the 39 proteins selected for experimental validation is provided as Dataset S2. Plasmid vector maps and representative images are available as Dataset S3 and S4. AlphaFold2 structures and images are provided as Dataset S5. The dataset of tested mutations is provided as Dataset S6. All datasets are deposited in the public Edmond repository of the MPG under the link https://doi.org/10.17617/3.0Y9Q8N. Source data are provided with this paper.
All code generated during this study is available at https://git.mpi-cbg.de/tothpetroczylab/picnic. Web-server is available at https://picnic.cd-code.org/. Python package is available at https://pypi.org/project/picnic-bio/1.0.0b1/.

Research involving human participants, their data, or biological material

Policy information about studies with human participants or human data. See also policy information about sex, gender (identity/presentation), and sexual orientation and race, ethnicity and racism.

Reporting on sex and gender	NA
Reporting on race, ethnicity, or other socially relevant groupings	NA
Population characteristics	NA
Recruitment	NA
Ethics oversight	NA

Note that full information on the approval of the study protocol must also be provided in the manuscript.

Field-specific reporting

Please select the one below that is the best fit for your research. If you are not sure, read the appropriate sections before making your selection.

[x] Life sciences [ ] Behavioural & social sciences [ ] Ecological, evolutionary & environmental sciences

For a reference copy of the document with all sections, see nature.com/documents/nr-reporting-summary-flat.pdf

Life sciences study design

All studies must disclose on these points even when the disclosure is negative.

Sample size	In this study we tested if a given fluorescently tagged protein (39 proteins in total) localises to a clearly visible mesoscale foci in cells or not when recorded on a spinning disk microscope. We transfected the relevant plasmid DNA's and observed and/or measured in at least 25 cells before concluding that such a protein actually forms a mesoscale foci.
Data exclusions	All the cells with clearly visible fluorescence expression were analyzed.
Replication	Experiments were repeated at least twice (N>=2) to ascertain the reproducibility with respect to a protein forming mesoscale foci in cells as well as the number of cells to determine the confidence in the observed foci.
Randomization	NA
Blinding	NA

Reporting for specific materials, systems and methods

We require information from authors about some types of materials, experimental systems and methods used in many studies. Here, indicate whether each material, system or method listed is relevant to your study. If you are not sure if a list item applies to your research, read the appropriate section before selecting a response.

Materials & experimental systems

n/a	Involved in the study
☒	☐ Antibodies
☐	☒ Eukaryotic cell lines
☒	☐ Palaeontology and archaeology
☒	☐ Animals and other organisms
☒	☐ Clinical data
☒	☐ Dual use research of concern
☒	☐ Plants

Methods

n/a	Involved in the study
☒	☐ ChIP-seq
☒	☐ Flow cytometry
☒	☐ MRI-based neuroimaging

Eukaryotic cell lines

Policy information about cell lines and Sex and Gender in Research

Cell line source(s)	U2OS cell line from MPI-CBG Central Facility
Authentication	The last authentication was done 2 years ago by Eurofins Genomics using STR profiling
Mycoplasma contamination	The cell lines were tested regularly, last time in March 2024
Commonly misidentified lines (See ICLAC register)	NA
