# Supplementary material for: PICNIC accurately predicts condensate-forming proteins regardless of their structural disorder across organisms
Source: Nat Commun. 2024 Dec 11;15:10668. doi: 10.1038/s41467-024-55089-x (PMC11634905; doi:10.1038/s41467-024-55089-x)
Supplement: Supplementary file 9 — Description of Additional Supplementary Files [file 41467_2024_55089_MOESM9_ESM.pdf]

- **Supplementary Dataset 1 (Dataset_S1_datasets.xlsx).** List of proteins in the training, validation and test datasets.

- **Supplementary Dataset 2 (Dataset_S2_tested_proteins.xlsx).** List of 24 positive and 15 negative predicted proteins that were experimentally tested and their characteristics.

- **Supplementary Dataset 3 (Dataset_S3_plasmid_vector_maps.zip).** Plasmid vector maps of the 39 constructs used in this study.

- **Supplementary Dataset 4 (Dataset_S4_representative_images.zip).** Raw images of the 21 positive (Figure4_Positive_predictions and FigureS10_Representative Images) and 15 negative proteins (Figure S11_Negative_predictions) imaged in this study and the images of the FRAP experiments (Figure5_FRAP and Figure6_FRAP).

- **Supplementary Dataset 5 (Dataset_S6_sequences_with_mutations.xlsx).** Protein sequences that contain mutations from Table S1.

- **Supplementary Dataset 6 (Dataset_S5_AlphaFold_structures.zip).** AlphaFold2 models of the 39 proteins in PDB format.
