# Supplementary figures and images for: PICNIC accurately predicts condensate-forming proteins regardless of their structural disorder across organisms (part 3 of 3)
Source: Nat Commun. 2024 Dec 11;15:10668. doi: 10.1038/s41467-024-55089-x (PMC11634905; doi:10.1038/s41467-024-55089-x)

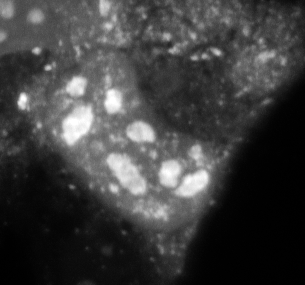

Supplement: Supplementary file 11 — Source Data files [file 41467_2024_55089_MOESM11_ESM.zip › Source_Data_file/main/Figure_5/Fibrillarin + R51A1_colocalization/4_GFP-4.tif]

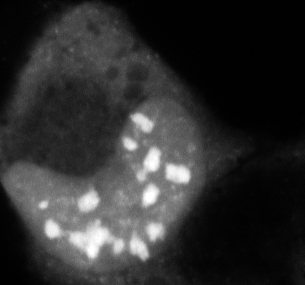

Supplement: Supplementary file 11 — Source Data files [file 41467_2024_55089_MOESM11_ESM.zip › Source_Data_file/main/Figure_5/Fibrillarin + R51A1_colocalization/3_GFP_3.tif]

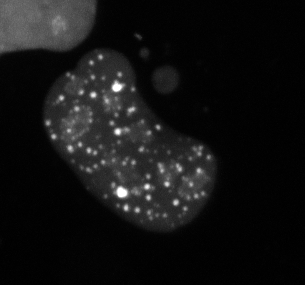

Supplement: Supplementary file 11 — Source Data files [file 41467_2024_55089_MOESM11_ESM.zip › Source_Data_file/main/Figure_5/Fibrillarin + R51A1_colocalization/4_iRFP-4.tif]

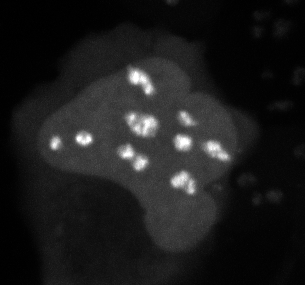

Supplement: Supplementary file 11 — Source Data files [file 41467_2024_55089_MOESM11_ESM.zip › Source_Data_file/main/Figure_5/Fibrillarin + R51A1_colocalization/2_GFP_2.tif]

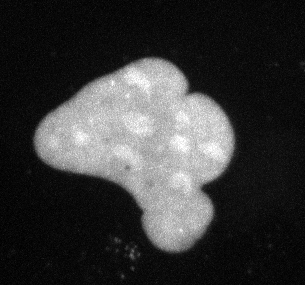

Supplement: Supplementary file 11 — Source Data files [file 41467_2024_55089_MOESM11_ESM.zip › Source_Data_file/main/Figure_5/Fibrillarin + R51A1_colocalization/2_iRFP_2.tif]

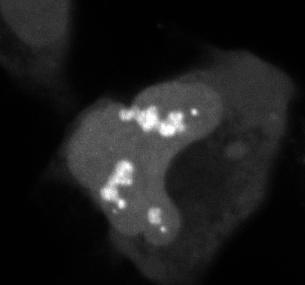

Supplement: Supplementary file 11 — Source Data files [file 41467_2024_55089_MOESM11_ESM.zip › Source_Data_file/main/Figure_5/Fibrillarin + R51A1_colocalization/1_GFP.tif]

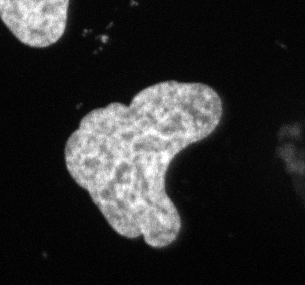

Supplement: Supplementary file 11 — Source Data files [file 41467_2024_55089_MOESM11_ESM.zip › Source_Data_file/main/Figure_5/Fibrillarin + R51A1_colocalization/1_iRFP.tif]

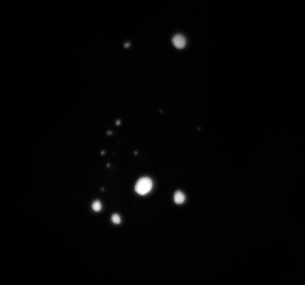

Supplement: Supplementary file 11 — Source Data files [file 41467_2024_55089_MOESM11_ESM.zip › Source_Data_file/main/Figure_5/DCP1a + PHP14_colocalization/GFP1.tif]

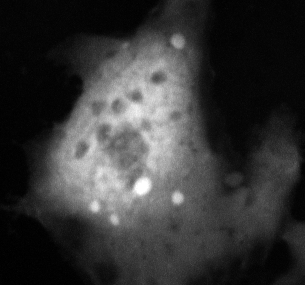

Supplement: Supplementary file 11 — Source Data files [file 41467_2024_55089_MOESM11_ESM.zip › Source_Data_file/main/Figure_5/DCP1a + PHP14_colocalization/iRFP1.tif]

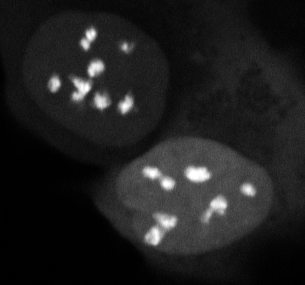

Supplement: Supplementary file 11 — Source Data files [file 41467_2024_55089_MOESM11_ESM.zip › Source_Data_file/main/Figure_5/Fibrillarin + H2A1H_colocalization/1_GFP.tif]

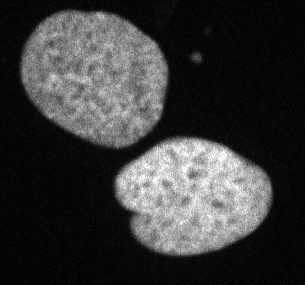

Supplement: Supplementary file 11 — Source Data files [file 41467_2024_55089_MOESM11_ESM.zip › Source_Data_file/main/Figure_5/Fibrillarin + H2A1H_colocalization/1_iRFP.tif]

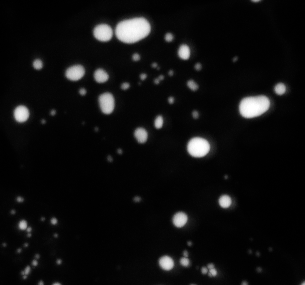

Supplement: Supplementary file 11 — Source Data files [file 41467_2024_55089_MOESM11_ESM.zip › Source_Data_file/main/Figure_5/DCP1a + HBS1L_colozalization/GFP.tif]

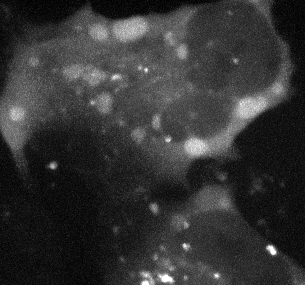

Supplement: Supplementary file 11 — Source Data files [file 41467_2024_55089_MOESM11_ESM.zip › Source_Data_file/main/Figure_5/DCP1a + HBS1L_colozalization/iRFP.tif]
